# Multimaterial Thermoset Synthesis: Switching Polymerization Mechanism with Light Dosage

**DOI:** 10.1021/acscentsci.4c01507

**Published:** 2024-11-12

**Authors:** Yuting Ma, Reagan J. Dreiling, Elizabeth A. Recker, Ji-Won Kim, Shelby L. Shankel, Jenny Hu, Alexandra D. Easley, Zachariah A. Page, Tristan H. Lambert, Brett P. Fors

**Affiliations:** † Department of Chemistry, Cornell University, Ithaca, New York 14853, United States; ‡ Department of Chemical Engineering, The University of Texas at Austin, Austin, Texas 78712, United States; § Department of Chemistry, The University of Texas at Austin, Austin, Texas 78712, United States

## Abstract

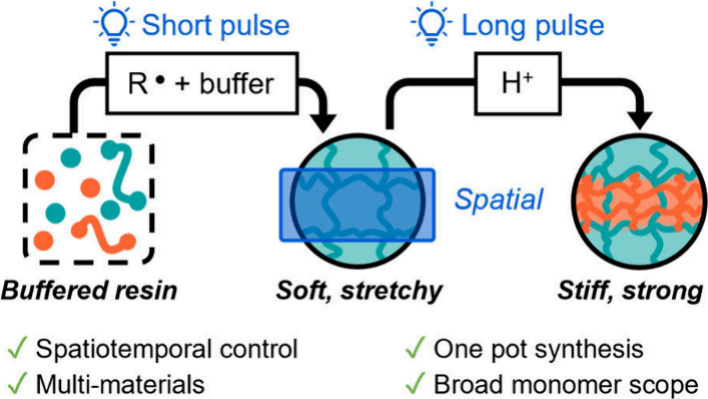

The synthesis of polymeric thermoset materials with spatially controlled physical properties using readily available resins is a grand challenge. To address this challenge, we developed a photoinitiated polymerization method that enables the spatial switching of radical and cationic polymerizations by controlling the dosage of monochromatic light. This method, which we call Switching Polymerizations by Light Titration (SPLiT), leverages the use of substoichiometric amounts of a photobuffer in combination with traditional photoacid generators. Upon exposure to a low dose of light, the photobuffer inhibits the cationic polymerization, while radical polymerization is initiated. With an increased light dosage, the buffer system saturates, leading to the formation of a strong acid that initiates a cationic polymerization of the dormant monomer. Applying this strategy, patterning is achieved by spatially varying light dosage via irradiation time or intensity allowing for simple construction of multimaterial thermosets. Importantly, by the addition of an inexpensive photobuffer, such as tetrabutylammonium chloride, commercially available resins can be implemented in grayscale vat photopolymerization 3D printing to prepare sophisticated multimodulus constructs.

## Abstract 2

We employ weakly basic anionic photobuffers to decouple two photopolymerizations in one pot. The photobuffer enables tuning of multimaterials by adjusting the dosage of a single wavelength of light.

## Introduction

Photopolymerization facilitates rapid and controllable polymer synthesis, and its application in additive manufacturing (e.g., 3D printing) has enabled the fabrication of sophisticated structures with precision in both form and function. Materials with spatially tuned properties are highly desirable in areas such as electronics and soft robotics.^1−8^ However, typical multimaterial synthesis requires multiple individual resins, leading to material waste, lengthy manufacturing, and often weak interfaces, limiting their production.^9−11^

Wavelength-orthogonal processes have recently been developed to selectively cure dual materials from a single resin, but these strategies necessitate wavelength-selective photocatalysts and customized manufacturing systems due to the lack of commercially available multiwavelength 3D printers.^12−25^ Additionally, Page and co-workers have demonstrated a dual-catalyst-initiated ring-opening metathesis polymerization to control the stereochemistry and mechanical properties of polyoctenamers with monochromatic light,^26^ and more recently wavelength-selective thiol–ene resins for multimodulus 3D printing.^27^ Still, the materials scope is limited, and the properties are controlled by either a combination of thermal and photomediated processes or the need for two light sources. On this basis, a general synthetic strategy that can produce materials with spatially controlled physical properties in one pot using a single wavelength of light at room temperature remains a highly desirable goal.

**Figure 1:**
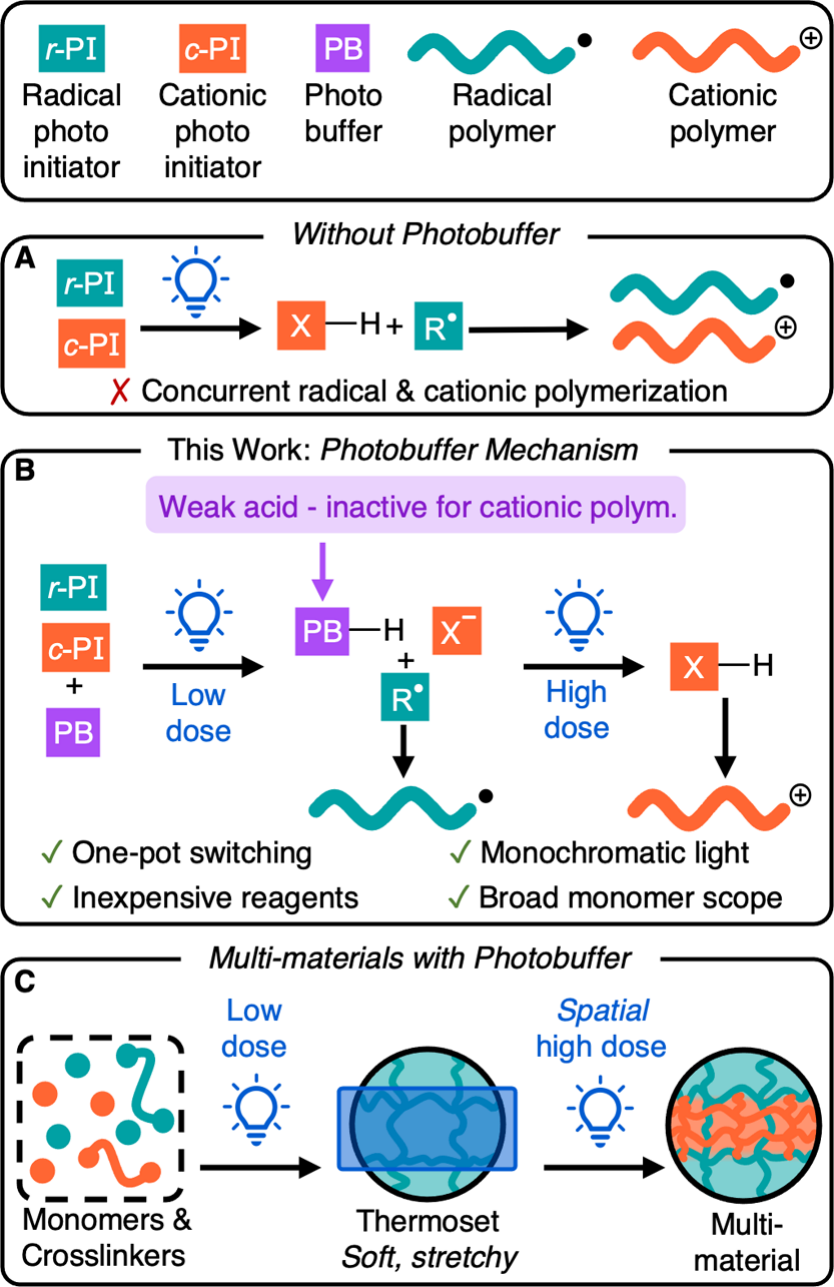
Switching polymerization mechanisms using a photobuffer. (A) Nonselective photoinitiated radical and cationic polymerizations without a photobuffer. (B) One pot switching radical and cationic polymerizations with a photobuffer. (C) Spatial control of multimaterial properties with monochromatic light, where the blue rectangle represents the area of higher light exposure.

Photoinitiated free radical and cationic polymerizations are extensively used in photolithography, additive manufacturing, and coating industries.^28^ Type I and II photoinitiators promote rapid radical polymerizations;^29−32^ meanwhile, photoacid generators (PAGs) such as “onium” salts, generate strong Brønsted acids for curing monomers that polymerize through a cationic mechanism.^33−35^ Using a combination of a photoacid generator and radical photoinitiator, cationic and radical polymerizations can be performed simultaneously to produce thermosets that contain dual networks (Figure 1A). The ability to temporally control each polymerization would allow for the synthesis of materials with spatially controlled physical properties; however, decoupling the two photoinitiated polymerizations in one pot with one wavelength of light remains a challenge.

To develop a general one-pot method using a single wavelength of light, we envisioned a system where the polymerization mechanism could be switched in response to the amount of light added (light dosage). Here, we report the realization of such a strategy, which we term the Switching Polymerization Mechanism by Light Titration (SPLiT). To achieve this goal in a system that contains a PAG, we reasoned that the addition of a substoichiometric amount of a weakly basic anion would act as a photobuffer to enable spatial control over the radical and cationic polymerizations. Specifically, upon irradiation with a low dose of light (i.e., a short irradiation time at a constant intensity), the strong acid generated from the PAG would be sequestered by the weakly basic anion and, thus, buffer the solution to inhibit cationic polymerization. Meanwhile, the radical photoinitiator would not be impacted by the photobuffer and could still initiate the radical polymerization. With continued irradiation to increase light dosage (i.e., a long irradiation time at a constant intensity), the photobuffer would be completely consumed, and strong acid generated from the PAG would switch on cationic polymerization (Figure 1B). Integral to SPLiT, a strong acid is formed only in areas where the light dosage overcomes the amount of photobuffer added to the resin, leading to spatial control over cationic polymerization (Figure 1C). In this way, SPLiT provides a general strategy for producing polymeric multimaterials in one-pot with a single wavelength of light that could simplify the photoprinting of multimaterials from a single resin.

## Results and Discussion

**Figure 2:**
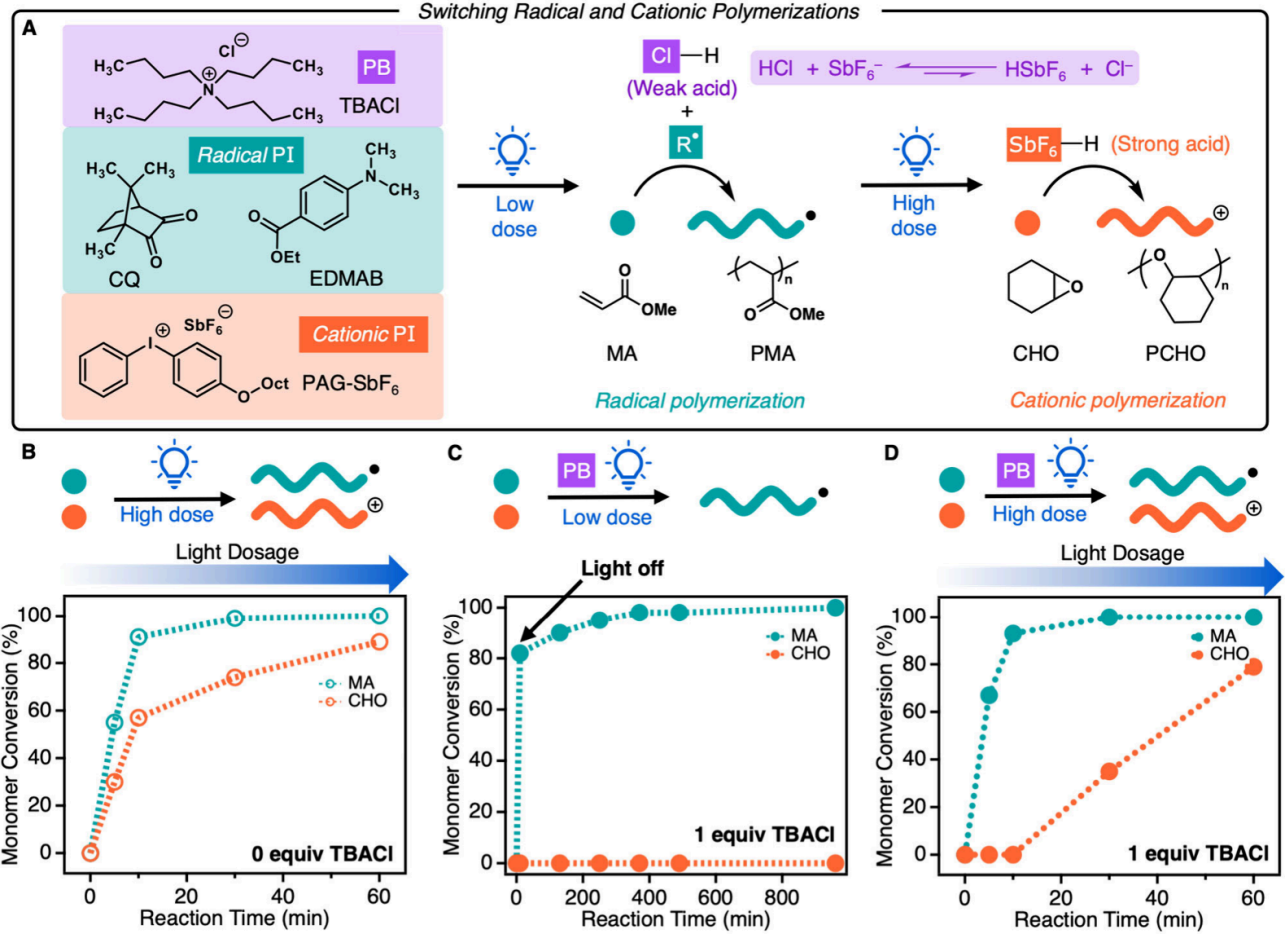
Photobuffer mediated selective radical and cationic polymerizations. (A) Photobuffer (TBACl) delays HSbF_6_ generation from a low light dose (10 min exposure), allowing radical polymerization to proceed before cationic polymerization. (B) Without a photobuffer, both MA radical polymerization and CHO cationic polymerization were initiated with a high light dose (60 min exposure). (C) With TBACl and a low light dose, only MA was consumed. (D) With TBACl and a high light dose, MA and CHO were sequentially polymerized. Experimental conditions for (B) were 2 equiv of PAG-SbF_6_, 1 equiv of CQ, 1 equiv of EDMAB, 100 equiv of MA, and 100 equiv of CHO at rt under N_2_ with 455 nm light irradiation (0.14 mW/cm^2^). Experimental conditions for (C) and (D) were 1 equiv of TBACl, 2 equiv of PAG-SbF_6_, 1 equiv of CQ, 1 equiv of EDMAB, 100 equiv of MA, and 100 equiv of CHO at rt under N_2_ with 455 nm light irradiation (0.14 mW/cm^2^).

We began by investigating the addition of tetrabutylammonium chloride (TBACl) as a photobuffer to a photoinitiating system composed of a photosensitizer (camphorquinone, CQ), a hydrogen atom donor (ethyl 4-(dimethylamino)benzoate, EDMAB), and a photoacid generator ([4-[octyloxy]phenyl]phenyliodonium hexafluoroantimonate, PAG-SbF_6_) (Figure 2A). When excited by visible light, CQ is reduced by EDMAB, producing a ketyl radical and R^•^, which can initiate radical polymerization. The resultant CQ ketyl radical is oxidized back to its ground state by the PAG-SbF_6_ salt, producing H^+^ (Scheme S1).^36^ Under low light dosage, any H^+^ generated will be sequestered by TBACl to form the thermodynamically more favorable, weaker acid HCl. In the presence of radically and cationically polymerizable monomers, R^•^ will initiate free radical polymerization exclusively while cationic polymerization remains dormant.^37,38^ Upon increased light dosage, the TBACl photobuffer will be completely consumed, forming strong acid HSbF_6_, which will initiate cationic polymerization.

The temporal control of SPLiT was investigated using NMR spectroscopy by adding methyl acrylate (MA), which polymerizes radically, and cyclohexene oxide (CHO), which polymerizes cationically, to our initiating system. Without TBACl, consumption of both monomers was observed (86% MA and 53% CHO) after 10 min of blue light irradiation (455 nm LED, 0.14 mW/cm^2^), indicating simultaneous radical and cationic polymerizations (Figure 2B).^39^ In contrast, upon the addition of 0.5 equiv of TBACl relative to PAG-SbF_6_, radical polymerization of MA was exclusively observed, and no CHO conversion was seen (80% MA and 0% CHO) after 10 min of irradiation (low dose) (Figure 2C). Increasing the light exposure to 60 min (high dose) resulted in both radical and cationic polymerizations (100% MA and 79% CHO) (Figure 2D). We concluded that the addition of TBACl delays cationic initiation by buffering the generation of strong acid, thus facilitating the selective polymerization of acrylate and epoxide monomers through light dosage.

SPLiT was also demonstrated for other monomer classes. To expand the monomer scope of the radical polymerization, methyl methacrylate (MMA) was mixed with CHO. With TBACl present, low light dosage (1 h exposure) led only to polymerization of MMA, while increased light dosage (2 h exposure) polymerized both MMA and CHO (Figures S8–S9). To illustrate the extent of the cationic ring-opening polymerization, caprolactone (CL) and 1,3-dioxolane (DXL) were also studied in combination with MA. Rapid MA polymerization was observed in both the MA-CL and MA-DXL systems under a low light dose, while no cationic polymerization was detected in either reaction with TBACl present (Figures S11, S14). When the light exposure was extended to 16 h, the cationic polymerizations ran to high conversions (46% CL and 84% DXL) (Figures S12, S15). As supported by these studies, SPLiT is a general strategy to enable polymerization switching for a variety of monomers.

**Figure 3:**
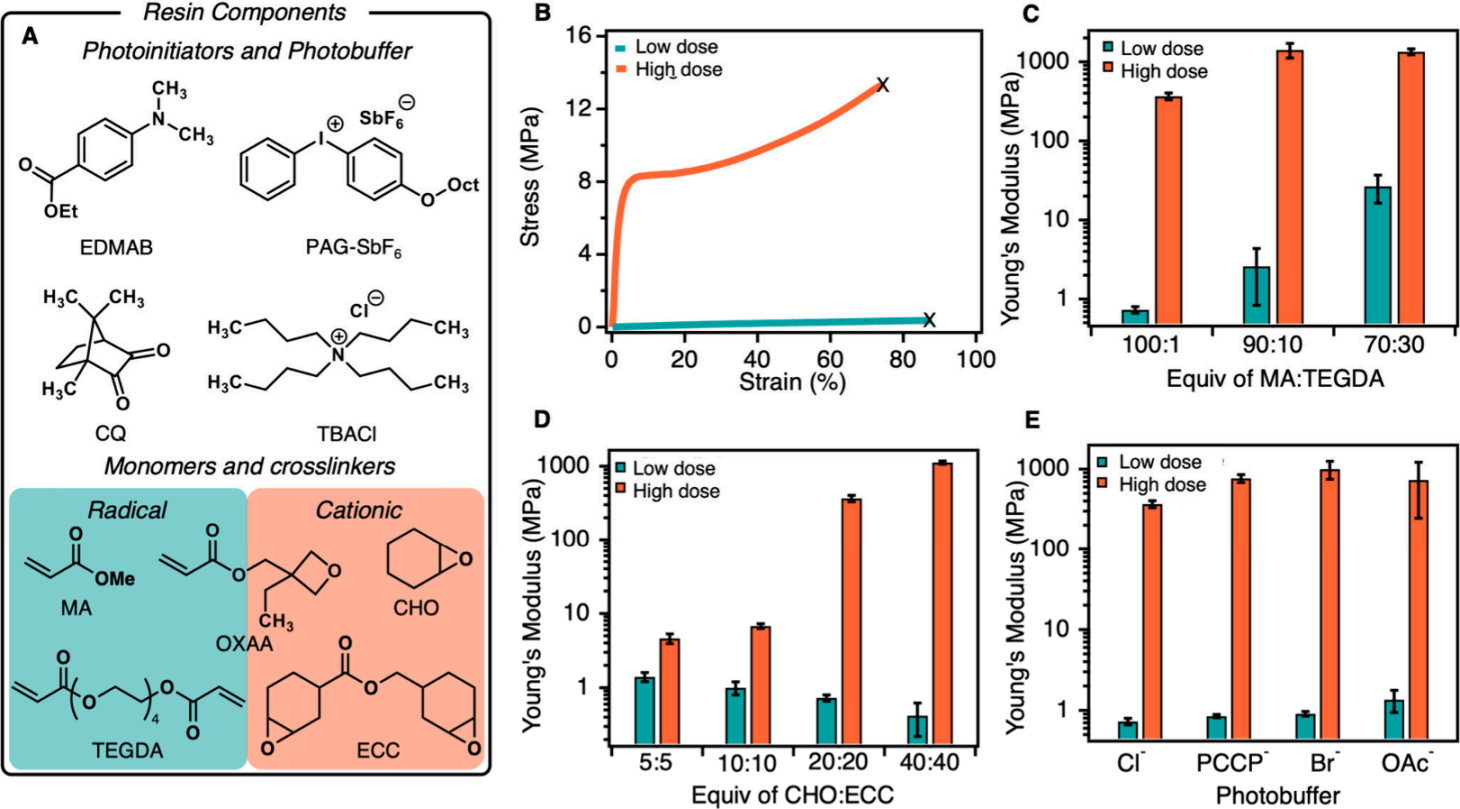
Multimaterial synthesis using SPLiT. (A) Acrylate-epoxide resin components. (B) Stress–strain curves of cross-linked acrylate-epoxide films made with a low dose (30 s) and high dose (1 h) of blue light (456 nm, 23 mW/cm^2^). (C) Varying MA:TEGDA ratios altered acrylate-epoxide thermoset Young’s moduli (*E*) under low light dosage. (D) Varying epoxide equivalents (both monomers and cross-linkers) tuned thermoset *E* under high light dosage. (E) *E* for thermosets produced from acrylate-epoxide resins with a variety of photobuffers (TBACl, TBAPCCP, TBAOAc, TBABr) under low and high light dosage. Photoinitiator conditions for (A)–(D) were 0.5 equiv of photobuffer, 1 equiv of PAG-SbF_6_, 1 equiv of CQ, and 1 equiv of EDMAB. Soft (low dose) films were swelled in 1:1 isopropanol:acetone overnight and dried in a vacuum oven at 50 °C overnight to remove unreacted residue prior to tensile testing. Tensile testing specimen dimensions were ca. 1.0 mm (T) × 5.0 mm (W) × 20 mm (L).

Next, we sought to apply SPLiT to multimaterial thermoset synthesis. Due to limited characterization methods for insoluble materials, monomers with drastic differences in thermomechanical properties were utilized to monitor thermoset synthesis. For example, alkyl acrylate polymers, such as poly(MA), exhibit low glass transition temperatures (*T*_g_’s) yielding soft and deformable materials, while polymers of cyclic epoxides, like poly(CHO), exhibit higher *T*_g_’s affording hard and strong materials.^12,40,41^ To this end, we added a diacrylate (tetra(ethylene glycol) diacrylate, TEGDA) and a diepoxide (3,4-epoxycyclohexylmethyl-3′,4′-epoxycyclohexane carboxylate, ECC) to cross-link the two polymers in the MA/CHO system. Additionally, acrylate oxirane ((3-ethyloxetan-3-yl)methyl acrylate, OXAA) was added to covalently link the two materials and prevent macrophase separation, as demonstrated by Hawker and co-workers (Figure 3A).^12^ With low light dosage (30 s, short exposure) followed by solvent washing and drying, a film with a Young’s modulus (*E*) of 0.7 MPa was formed, suggesting that acrylates were primarily incorporated (Figure 3B). Additional light dosage (1 h, long exposure) increased the *E* to 367 MPa, indicating a significant incorporation of the cyclic epoxides into the film. The consumption of CHO was also monitored by gas chromatography, which supports the above conclusion (Figures S23–S24). A high dose thermoset synthesized without photobuffer displayed a similar *E* of 564 MPa, further confirming the epoxide incorporation. (Figure S22, Table S4). These results demonstrate that materials with different physical properties can be fabricated by changing the light dosage.

The mechanical properties of acrylate-epoxide multimaterials can be systematically tuned by altering the ratio of monomers to cross-linkers. Under a low light dose, changing the ratio of MA:TEGDA from 100:1 to 70:30 increased *E* from 0.73 to 26.63 MPa, demonstrating the tunability of poly(methyl acrylate) cross-link density under low light dosage (Figure 3C). Increasing the light dosage resulted in similar *E* values regardless of the MA:TEGDA ratio, suggesting that under high light dosage mechanical properties converge to that of the cyclic epoxide thermosets. These results demonstrate that the soft phase properties can be independently tuned through the acrylate cross-linker composition while maintaining the hard phase stiffness. With a constant ratio of MA:TEGDA, increasing the epoxide equivalents (both monomer and cross-linker) from 5 to 40 under high light dosage increased *E* from 4.6 to 367 MPa (Figure 3D). Notably with a low light dosage, no significant change in the acrylate film stiffness was observed with altered epoxide equivalents. Therefore, under a given light dosage, the soft phase stiffness can be tuned via composition without effecting the hard phase and vice versa.

The initial system used TBACl as the photobuffer; however, we reasoned that any anion that produces a weak acid unable to initiate epoxide polymerization should give similar results. To investigate the photobuffer scope, tetrabutylammonium (TBA^+^) salts with a variety of coordinating anions were examined in the production of acrylate-epoxide thermosets. Bromide (Br^–^), pentacarbomethoxycyclopentadiene anion (PCCP^–^), and acetate (OAc^–^) salts all afforded elastomeric cross-linked films under low light dosage with *E* of 0.91, 0.85, and 1.36 MPa, respectively. High light dosage drastically increased the *E* to 998, 767, and 732 MPa, respectively (Figure 3E). These results demonstrate that SPLiT is not limited to the use of chloride, as other anions are competent photobuffers when their conjugate acid p*K*_a_’s are below the value needed to initiate cationic polymerization.

**Figure 4:**
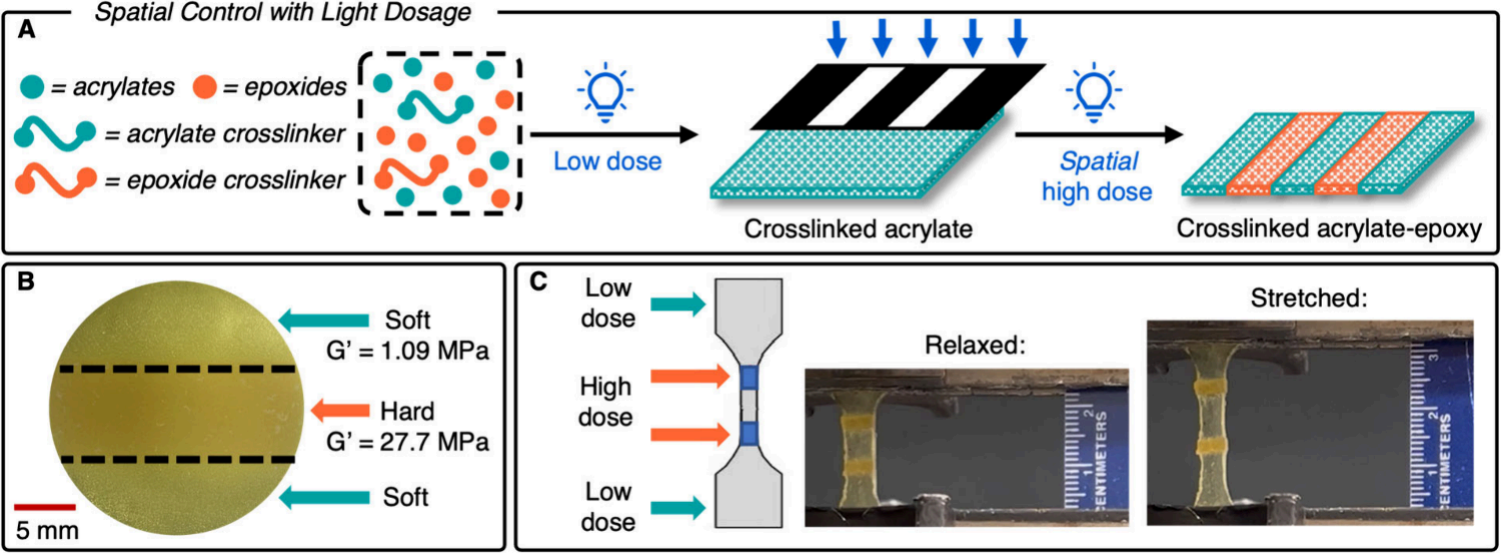
Spatial control of multimaterial properties using SPLiT. (A) A soft acrylate thermoset was formed upon low light dosage (short exposure time, 456 nm, 23 mW/cm^2^). Applying a photomask during a high light dose (455 nm, 86 mW/cm^2^) yielded an acrylate-epoxide thermoset with spatially controlled hard and soft domains. (B) The middle region of an acrylate-epoxide resin irradiated with high light dosage had a storage modulus that was 27× higher than that of the low dose irradiated outer regions. (C) A photopatterned film was subjected to tensile testing, where domains exposed to a low light dose underwent deformation. Experimental conditions for (A)-(C) were 0.5 equiv of photobuffer, 1 equiv of PAG-SbF_6_, 1 equiv of CQ, 1 equiv of EDMAB, 160 equiv of MA, 1.6 equiv of TEGDA, 20 equiv of CHO, 20 equiv of ECC, and 7 equiv of OXAA.

Having demonstrated temporal control in the initiation of the radical and cationic polymerizations using light dosage, we aimed to leverage this selectivity in spatial control of the thermoset physical properties. Photomasks were applied to spatially select areas of high light dosage where TBACl was consumed and led to stiffer epoxide thermoset formation; consequently, areas of low light dosage contained primarily soft cross-linked acrylates (Figure 4A).^18,42−45^ Irradiation with a low light dose (10 s) created a soft cross-linked film with a storage modulus (*G*′) of 1.09 MPa measured using oscillatory rheology (Figure 4B). Subjecting the midsection of the film to a high light dose (1 h) yielded a domain with an increased *G*′ of 27.7 MPa (Figure S26). This difference in *G*′ magnitude confirms that the photobuffer, an integral part of the SPLiT system, enables synthesis of multimaterials using a single wavelength of light.

To exemplify the ease and utility of this spatiotemporal control using SPLiT, a dogbone was produced that contained two small hard sections distributed within the soft material of the gauge length (Figure 4C).^12^ When the dogbone was stretched, the soft domains elongated to a much greater extent than did the hard domains (Figure S25). Overall, these results demonstrate the capability of SPLiT to make spatially distinct domains that impact the mechanical behavior of the resulting multimaterials from a single resin.

Next, we sought to demonstrate that SPLiT could be used by single-wavelength 3D printers to produce multimaterial objects without needing to change the light source or resin. While the light dosage for film syntheses was controlled by changing the exposure time at a constant light intensity, the light dosage in 3D printing was controlled by changing the light intensity at a constant exposure time per layer (i.e., grayscale projection).

**Figure 5:**
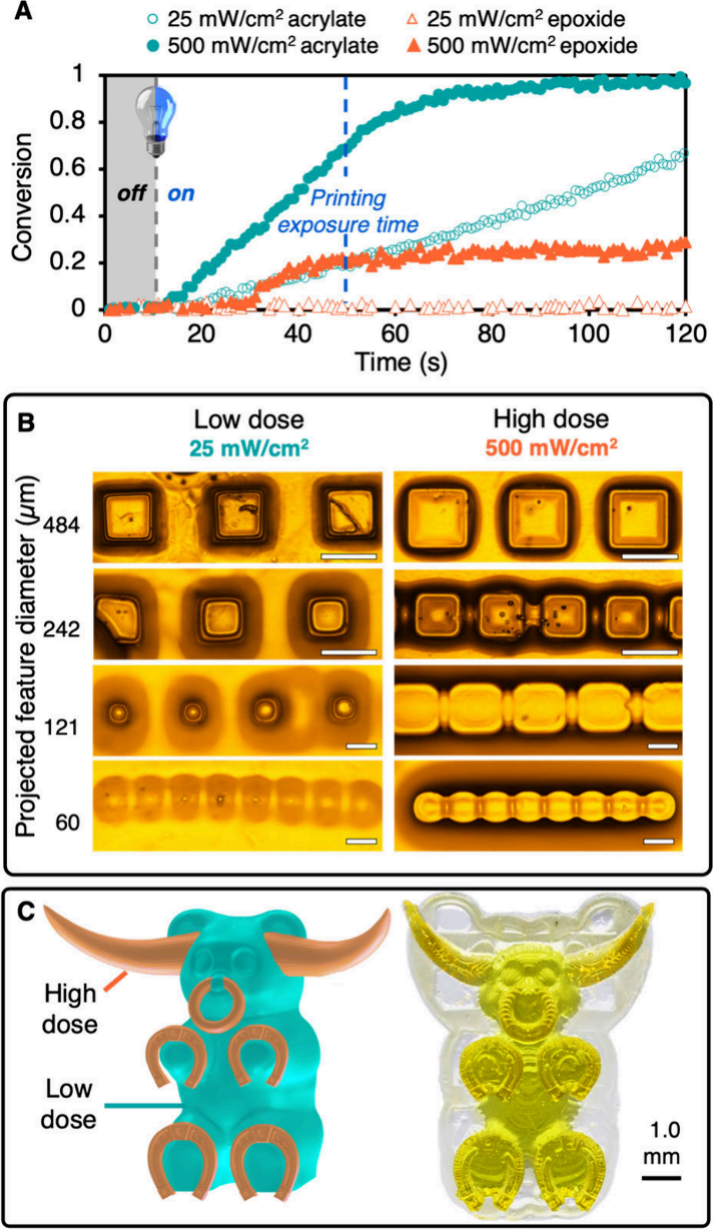
3D printing using SPLiT resins. (A) Under low light dosage (25 mW/cm^2^), only radical polymerization proceeds, while both radical and cationic polymerizations occur under a high light dosage (500 mW/cm^2^). Irradiation began at *t* = 10 s for all FTIR experiments. (B) Good resolution is maintained while printing soft and hard materials under low and high light doses. Scale bars for 64 and 128 pixel feature images are 500 μm, while scale bars for 16 and 32 pixel feature images are 100 μm. (C) Digital design and 3D printing of a multimaterial longhorn-bear hybrid. Exposure time was 40 s per 50 μm slice for all printed objects.

To identify the light dosage required to separate radical and cationic polymerizations on time scales relevant to 3D printing, the original SPLiT resin was irradiated with different intensities from a blue LED (460 nm peak) and monomer conversion was monitored in situ using real-time Fourier transform infrared spectroscopy (RT-FTIR) (Figures S30–31). At low light intensity, we observed only radical polymerization, which reached 49% conversion at 5 mW/cm^2^ and 70% conversion at 25 mW/cm^2^ after 2 min (Figures 5A, S30). At higher light intensities (60 mW/cm^2^ to 500 mW/cm^2^), the rate of acrylate polymerization increased and we observed initiation of epoxide polymerization due to sufficient acid generation (Figures 5A, S30–31). Furthermore, the light intensity could be adjusted to tune the “lag time” between the initial light exposure and cationic initiation. An intensity of 60 mW/cm^2^ resulted in a lag time of 80 s, whereas an intensity of 500 mW/cm^2^ shortened the lag time to 18 s (Figures S31–33). Notably, the thickness of the resin layer (50 μm vs 100 μm) had no measurable effect on conversion rate (Figures S34–S35). We concluded that a low light dosage of 25 mW/cm^2^ for 40 s/layer would be used to print soft domains, as this dose yielded high acrylate conversion without cationic initiation, whereas a high light dose of 500 mW/cm^2^ for 40 s/layer would be used for hard domains since this dose resulted in the fastest cationic polymerization.

Finally, we generated several 3D prints to highlight mechanical properties and spatial resolution achievable with SPLiT resins. Using a digital light processing (DLP) 3D printer equipped with a blue LED (458 nm peak) and an optimized SPLiT resin, we fabricated a multimaterial rectangle with one-half exposed to the low light dosage (25 mW/cm^2^ for 40 s/layer) and the other half exposed to high light dosage (500 mW/cm^2^ for 40 s/layer) (Figures S41, Table S6, for a full discussion of resin optimization, see the Supporting Information). Nanoindentation confirmed the difference in domain composition, as the low and high dose domains possessed *E* of 0.42 and 175.6 MPa, respectively (Figure S41). Next, an array of small cubes (16–128 pixels or 60–484 μm diameter) atop a square base was printed under both low and high doses to assess resolution (Figures S43–46). Microscopy images of both prints show high resolution down to 32 pixels (121 μm), making SPLiT resins comparable to other multimaterial resins and adaptable to commercial printers (Figure 5B).^12,46^ To showcase the ability of SPLiT to create complex multimaterial objects from a single resin and light source, we produced a custom file and print of a hybrid animal (longhorn-bear) having a soft body and hard horns, hooves, and nose ring (Figures 5C, S47–50). Nanoindentation of the bear body (*E* = 0.48 MPa) and cow horns (*E* = 154 MPa) revealed that the difference in mechanical properties from the simple rectangular print were conserved in this more sophisticated object (Figure S51).

While these prints were a successful proof-of-concept, SPLiT resins can be further optimized by investigating alternative photoinitiators (PIs), altering PI:PB ratios and including opaquing agents to speed print times and increase resolution. For instance, a “UV resin” formulated with 2-isopropylthioxanthone (ITX) as a radical 405 nm photoinitiator,^47^ rather than the relatively slow curing 460 nm CQ/EDMAB pair showed a dramatically decreased lag time from RT-FTIR analysis (Table S7, Figures S36–S37). A multimaterial tensile bar was successfully printed with this UV resin, demonstrating the generality of SPLiT for different wavelengths and photosystems (Figure S42) (a video of the dual properties can be found in the Supporting Information). Looking forward, adapting SPLiT to decouple other polymerization types, beyond radical and cationic, could facilitate 3D printing of polymeric multimaterials.^48^

## Conclusion

In conclusion, SPLiT is a simple, general method to switch polymerization mechanisms in one pot by tuning only the dosage of a single wavelength of light. A variety of weakly basic photobuffers were incorporated to sequester protons generated from the photolysis of PAGs and delay production of strong acid until full photobuffer consumption. This SPLiT strategy permitted radical polymerization of (meth)acrylates to proceed before the cationic polymerization of epoxides, lactones, and acetals. We extended the SPLiT strategy to achieve multimaterial thermoset synthesis with one wavelength of light. By employing photomasks during the second polymerization, we created spatially distinct domains with tunable mechanical properties within a single material. Finally, we demonstrated that SPLiT methodology enabled 3D printing of complex multimaterial objects without the need for multiple light sources, specialized photoresins, and lengthy fabrication processes.

## Supporting Information Available

Additional experimental details, materials, and methods, including photographs of experimental setup (PDF)
Video showing the dual properties of a multimaterial tensile bar (MOV)

## Supplementary Material

oc4c01507_si_001.pdf

oc4c01507_si_002.mov
