# Supplementary material for: Multimaterial Thermoset Synthesis: Switching Polymerization Mechanism with Light Dosage
Source: ACS Cent Sci. 2024 Nov 12;10(11):2125–31. doi: 10.1021/acscentsci.4c01507 (PMC11613345; doi:10.1021/acscentsci.4c01507)
Supplement: Supplementary file 1 — oc4c01507_si_001.pdf [file oc4c01507_si_001.pdf]

Supporting Information

# Multi-material Thermoset Synthesis: Switching Polymerization Mechanism with Light Dosage

**Yuting Ma,[†,||] Reagan J. Dreiling,[†,||] Elizabeth A. Recker,[‡] Ji-Won Kim,[§] Shelby L. Shankel,[†] Jenny Hu,[†] Alexandra D. Easley,[†] Zachariah A. Page,*[,§] Tristan H. Lambert,*[,†] Brett P. Fors*[,†]**

[†] Department of Chemistry, Cornell University, Ithaca, New York 14853, United States

[‡] Department of Chemical Engineering, The University of Texas at Austin, Austin, Texas 78712, USA

[§] Department of Chemistry, The University of Texas at Austin, Austin, Texas 78712, USA

Corresponding to: *Email: bpf46@cornell.edu, tristan.lambert@cornell.edu, zpage@utexas.edu

[||] Y.M. and R.J.D. contributed equally to this publication

General Reagent Information ........ 3
General Instrumentation Details ........ 3
Synthesis of AgPCCP ........ 6
Procedures for Control MA-CHO Polymerizations without Photobuffer ........ 10
Procedures for Switching MA-CHO Polymerizations with Photobuffer TBACl ........ 11
Procedures for Control MMA-CHO Polymerizations without Photobuffer ........ 13
Procedures for Switching MMA-CHO Polymerizations with Photobuffer TBACl ........ 14
Procedures for Control MA-CL Polymerizations without Photobuffer ........ 16
Procedures for Switching MA-CL Polymerizations with Photobuffer TBACl ........ 17
Procedures for Control MA-DXL Polymerizations without Photobuffer ........ 19
Procedures for Switching MA-DXL Polymerizations with Photobuffer TBACl ........ 20
Unsuccessful Switching MA-CHO Polymerizations with Pyridine Photobuffer ........ 22
General Procedure for Making Acrylate-Epoxide Thermoset with Low Light Dose ........ 24
General Procedure for Making Acrylate-Epoxide Thermoset with High Light Dose ........ 24
General Procedure for Spatial Control of Thermoset Properties ........ 24
**Table S1.** Resin Components in Figure 3C ........ 26
**Table S2.** Resin Components in Figure 3D ........ 27
**Table S3.** Resin Components in Figure 3E ........ 29

**Table S4.** Young's Modulus Values in Fig 3 B-E, Fig S22. 35
Gel Fraction Measurements 36
**Table S5**. Gel fractions of thermosets in Fig 3D. 36
GC Experiments and Results 37
Procedure for Cyclic Tensile Testing of Spatially Patterned Films 39
DMA Experiments and Results 40
FTIR and Printer LED Spectra 41
Resin Formulation Optimization 42
**Table S6.** Blue light resin formulation for 460 nm DLP 3D printing 42
**Table S7.** Violet light resin formulation for 405 nm DLP 3D printing 42
Kinetic Analysis via RT-FTIR Spectroscopy 43
Grayscale File Generation 48
Complex Grayscale Projections for 3D Printing 49
Multi-Material Bar Printing 50
Resolution Prints 52
Multi-Material Hybrid Longhorn-Bear Print 54
References. 57

***General Reagent Information***

No unexpected or unusually high safety hazards were encountered. Monomers methyl acrylate (MA, 99%, Sigma Aldrich, contains ⩽100 ppm monomethyl ether hydroquinone as inhibitor), methyl methacrylate (MMA, 99%, Sigma Aldrich, contains ⩽ 30 ppm monomethyl ether hydroquinone as inhibitor), cyclohexene oxide (CHO, 98%, Sigma Aldrich), ε-caprolactone (CL, 99%, Oakwood Chemical), and 1,3-dioxolane (DXL, Oakwood Chemicals, 99%) were dried over calcium hydride ($CaH_2$, ACROS organics, 93% extra pure, 0-2 mm grain size) overnight, distilled under nitrogen followed by 3 freeze-pump thaw cycles and then stored in the glove box freezer (−35 ˚C). Tetra (ethylene glycol) diacrylate (TEGDA, >90.0% stabilized with MEHQ, TCI), 3,4-epoxycyclohexylmethyl 3,4-epoxycyclohexanecarboxylate (ECC, Sigma Aldrich), (±)-Camphorquinone (CQ, 98%, TCI), ethyl 4-(dimethylamino)benzoate (EDMAB, 99%, Sigma Aldrich), tetrabutylammonium chloride (TBACl, Sigma Aldrich), [4-[octyloxy]phenyl]phenyliodonium hexafluoroantimonate (PAG-$SbF_6$, 95%, AstaTech) were dried under vacuum and used in glovebox. Triethylamine (TEA, EMD Millipore), acryloyl chloride (⩾ 97%, Lancaster Synthesis), methanol (MeOH, Fischer Chemical), silver acetate (AgOAc, Millipore Sigma), diphenyliodonium chloride (PAG-Cl, TCI, >98%), anhydrous pyridine (>99.8%, Millipore Sigma), 2,2,6,6-tetramethyl-1-piperidinyloxy (TEMPO, Sigma Aldrich, >99%), and 2-isopropyl thioxanthone (ITX, Combi-Blocks, 98%) were used as received. Dichloromethane (DCM) was purchased from J.T. Baker and purified by vigorous purging with argon for 2 h, followed by passing through two packed columns of neutral alumina under argon gas on the JC Meyer solvent system. Silicone molds were purchased on Sophie & Toffee and used as received. (3-ethyloxetan-3-yl)methyl acrylate (OXAA) was synthesized according to literature procedures.[1,2] 1,2,3,4,5- Pentacarbomethoxycyclopentadiene (PCCPH) was synthesized according to a reported literature procedure.[3,4]

***General Instrumentation Details***

All polymerizations to form linear polymers for NMR analysis were set up in a Unilab MBraun glovebox with a nitrogen atmosphere and irradiated with blue LEDs (450 nm, 0.14 mW/cm$^2$). All thermosets syntheses were set up in a Unilab MBraun glovebox with a nitrogen atmosphere and irradiated with a blue Kessil lamp (PR160L 456 nm, 23 mW/cm$^2$ at reaction surface), and spatially patterned thermosets were irradiated with a blue Thorlabs collimated LED (455 nm, 500 mW, 86 mW/cm$^2$ at reaction surface) under a nitrogen atmosphere outside the glovebox.

Uniaxial tensile testing studies were performed using a Shimadzu Autograph AGS-X tensile tester with pneumatic grips and 500 N load cell. ASTM D-1708 standard dogbone-shaped samples (*ca.* 1.0 mm (T) × 5.0 mm (W) × 20 mm (L) with gauge lengths measured from grip to grip) were elongated at 22 mm/min until break. Data analysis was performed using TrapeziumX v. 1.5.1 software. Young's modulus ($E$) was calculated using the slope of linear elastic region at low strain. Cyclic tensile testing was performed on a Zwick/Roell Z010 testing system equipped with screw grips. A cyclic loading of 25% strain at 25 mm/min was applied for 5 cycles. Curves were smoothed in Igor Pro 9 (binomial smoothing with 50 passes) due to noisy data.

Nuclear magnetic resonance (NMR) spectra were recorded on a Bruker 500 MHz instrument.

GC-FID chromatograms were recorded on a Shimadzu GC-2010 with an Equity-1701 column (0.25 µm film thickness, 0.25 mm I.D., 3 m length). The temperature was programmed as follows: injection at 150 °C, oven ramping from 140 to 150 °C at a rate of 2 °C/min, and FID at 200°C. Helium was used as a carrier gas at a flow rate of 50 mL/min. Chromatographic data were processed using OriginPro software and normalized to the internal standard.

DMA tests were performed on a TA Instruments DHR-20 rheometer using a tension fixture (rectangle). Strain sweeps (0.01–100%) at 25 °C were first performed at 1 Hz to determine the linear viscoelastic region. 1% strain was selected as it was within the linear viscoelastic region. Storage modulus (G') profiles were obtained at 25 °C and 1% strain with frequency oscillation from 0.1 to 10 Hz. Each sample was tested three times.

Real-Time Fourier Transform Infrared Spectroscopy (RT-FTIR) was conducted using an FTIR spectrometer (INVENIO-R, Bruker) to monitor radical and cationic polymerizations over time under a light irradiation. A horizontal transmission accessory (A043-N/Q, Bruker) was used to record real-time measurements, and a 460 nm LED (Type H, Mightex) or 405 nm LED (Type B, Mightex) was connected with an internal 3 mm liquid light guide (LLG-3-4H) to irradiate light after 10 s of measurement.

3D printing was performed using a custom-made, digital light processing (DLP) 3D printer (MONO3-VZ1, Monoprinter). The printer contains an LED projector from Visitech (LRS-WQ SL LC) with a blue LED having a central wavelength of 458 nm and a 17 nm full width at half maximum (FWHM) spectral width (for printing with PAG/CQ/EDMAB photosystem) or a violet LED having a central wavelength of 405 nm with a 13 nm FWHM spectral width (for printing with the PAG/ITX system). The projector resolution with an LRS-05 lens is 1600 × 2560 pixels, with each pixel being 3.78 µm × 3.78 µm at the image plane. Digital files for each print were generated by designing the 3D models using computer-aided design (CAD) software (SolidWorks) and exported into an STL format. Each STL file was imported into MonoWare software and supports were added as needed. Then, the 3D model was sliced into 2D MNF files with a slice thickness of 50 µm. The exposure times per slice were set to 40 seconds and light intensities were controlled with digital-to-analog converter (DAC) values that correspond to direct voltage applied to the LED. During a print, each image from the MNF file was projected onto the bottom of a resin vat with maximum image plane dimensions of 9.68 mm × 6.05 mm. A transparent fluorinated ethylene propylene (FEP) polymer film (Teflon FEP film, 127 µm thick, DuPont) was used as the base of the resin vat to provide a non-stick bottom surface.

Nanoindentation of patterned samples was performed using a Hysitron TI 950 TriboIndenter. Reduced modulus ($E_r$) was calculated using Hysitron software by fitting the unloading force-displacement curve using the Berkovich probe (TI-0039) tip area calibration. Hard samples were loaded to a maximum displacement of 2500 nm over a 5 second period, kept at that displacement for 5 seconds, and then unloaded over a 5 second period. Soft samples were loaded to a maximum displacement of 5000 nm over a 5 second period, kept at that displacement for 5 seconds, and then unloaded over a 5 second period. Equation 1 was used to convert all $E_r$ data to elastic modulus ($E$) using the following equation:

**Equation S1**: $$E = \frac{(1-\nu_s^2)}{\frac{1}{E_r} - \frac{(1-\nu_i^2)}{E_i}}$$

where $\nu_s$ is the sample poisson's ratio, $\nu_i$ is the indenter poisson's ratio, and $E_i$ is the indenter tip modulus. A $\nu_s$ of 0.4 was used as an estimate given that it represents a common value for acrylic polymers.[5,6] The berkovich indenter tip (Bruker, TI-0039 probe) has a reported $\nu_i$ of 0.07 and $E_i$ of 1140 GPa.

A digital microscope (VHX-7000, Keyence and Edge WF4915ZT, Dino-Lite) was utilized to image 3D printed structure. A 30× magnification lens was used to take images, and a transparent plate was used as a substrate. The camera was tilted to 20 degrees for the side view.

An optical microscope (Eclipse LV100ND, Nikon) was utilized to image hard and soft resolution prints and assess pixel resolution using 5-10× magnification. A halogen lamp (LV-LH50PC-CH, Nikon) was used to illuminate the top surface of prints for imaging.

***Synthesis of AgPCCP***

A 250 mL round bottom flask was charged with AgOAc (0.338 g, 2.02 mmol, 1 equiv) and 20 mL anhydrous MeOH. PCCPH (0.722 g, 2.02 mmol, 1 equiv) was dissolved in 44 mL of MeOH and added to the AgOAc solution. The reaction was stirred under air for 15 min before filtering out the solid impurity. The resultant solution was concentrated down on rotavap and dried under high vac overnight wrapped in aluminum foil to avoid light. This led to a yield of 838 mg of a light pink brown solid (90% yield). $^1$H NMR (400 MHz, $CD_3OD$): 3.72 ppm (s, 15H).
1Synthesis of TBA-PCCP

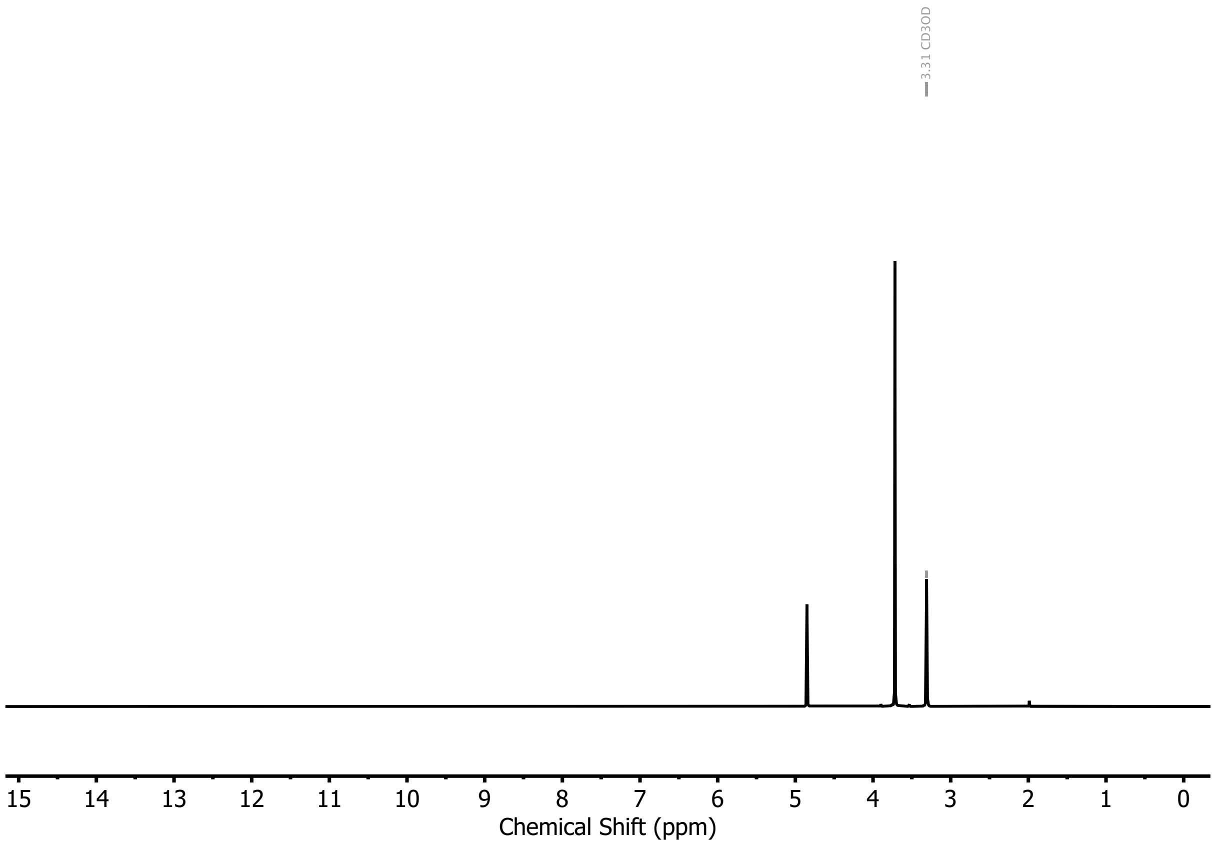


**Figure S1.** $^1$H NMR of AgPCCP in $CD_3OD$.

A round bottom flask was charged with AgPCCP (300 mg, 0.65 mmol, 1 equiv) and evacuated and backfilled with nitrogen 3 times. Following this, 5 mL of acetone were added under $N_2$. TBACl (190 mg, 0.68 mmol, 1.05 equiv) was quickly added to AgPCCP solution and stirred overnight. The reaction was filtered, concentrated on a rotavap, and dried under high vacuum overnight to obtain TBAPCCP (95% yield). $^1$H NMR (400 MHz, $CD_3OD$): 1.03 ppm (t, 12H), 1.41 ppm (sextet, 8H), 1.65 ppm (m, 8H), 3.22 ppm (m, 8H), 3.71 ppm (s, 15H). $^1$H NMR (400 MHz, $CD_3CN$): 0.97 ppm (t, 12H), 1.35 ppm (sextet, 8H), 1.60 ppm (m, 8H), 3.07 ppm (m, 8H), 3.63 ppm (s, 15H).

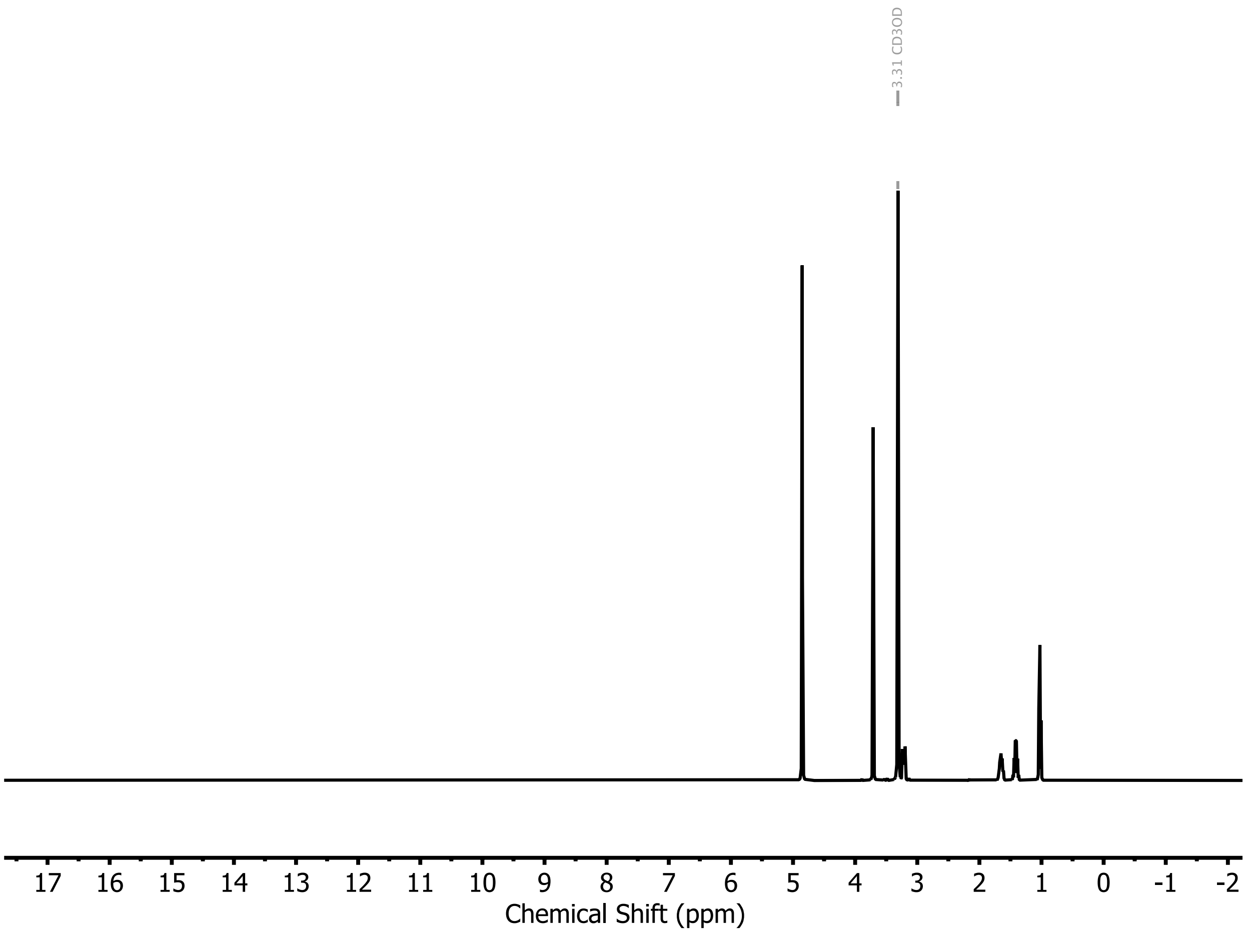


**Figure S2.** $^{1}H$ NMR of TBAPCCP in $CD_3OD$.

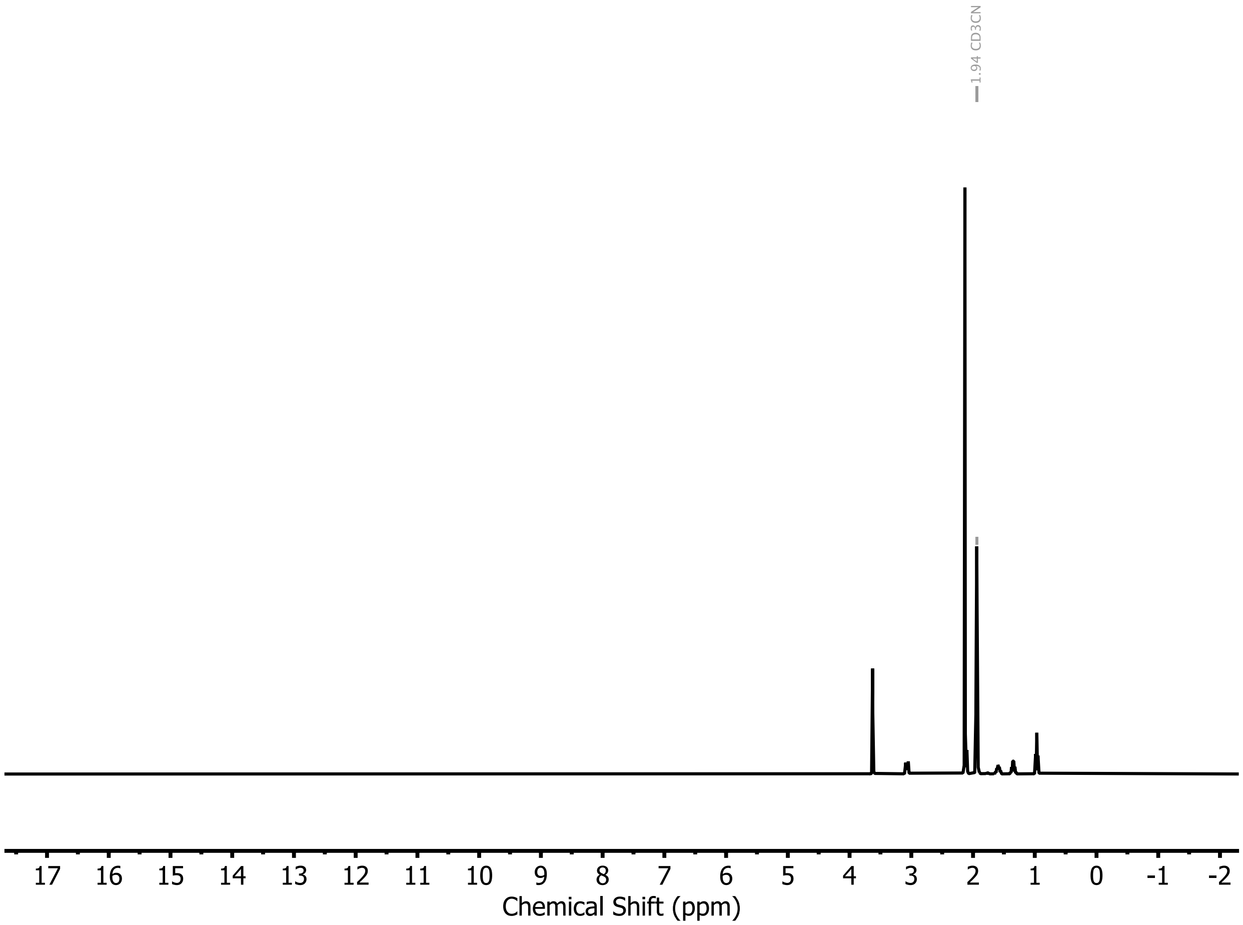


**Figure S3.** $^{1}$H NMR of TBAPCCP in $CD_3CN$.

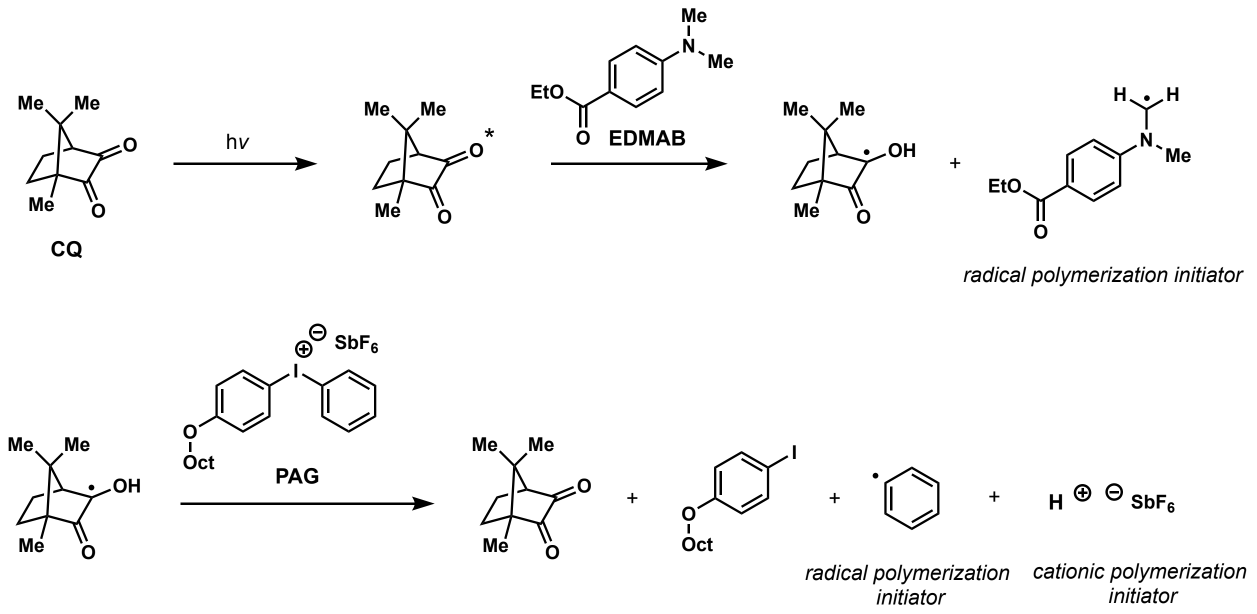


**Scheme S1.** Generation of polymerization initiators under blue light irradiation. Upon photoexcitation, CQ abstracts a hydrogen atom from EDMAB, generating an α-amino radical, which can initiate radical polymerization (top). The CQ ketyl radical is then oxidized and turned over by PAG. Rapid proton transfer and fragmentation produces an iodoarene, an aryl radical (another radical polymerization initiator), and H-$SbF_6$ (cationic polymerization initiator) (bottom).

***Procedures for Control MA-CHO Polymerizations without Photobuffer***

In a one-dram vial, CQ (1.65 mg, 0.01 mmol, 1 equiv), EDMAB (1.93 mg, 0.01 mmol, 1 equiv), and PAG-$SbF_6$ (12.9 mg, 0.02 mmol, 2 equiv) were added and dried under vacuum. After drying the vial was cycled between $N_2$ and vacuum three times. MA (0.1 ml, 1 mmol, 100 equiv) and CHO (0.1 ml, 1 mmol, 100 equiv) were added under an $N_2$ atmosphere and irradiated with blue LED strips (450 nm). Aliquots for $^1$H NMR analysis were taken at 5 min, 10 min, 30 min, and 60 min light irradiation and quenched with 0.1 mL of 5 vol% TEA in MeOH.

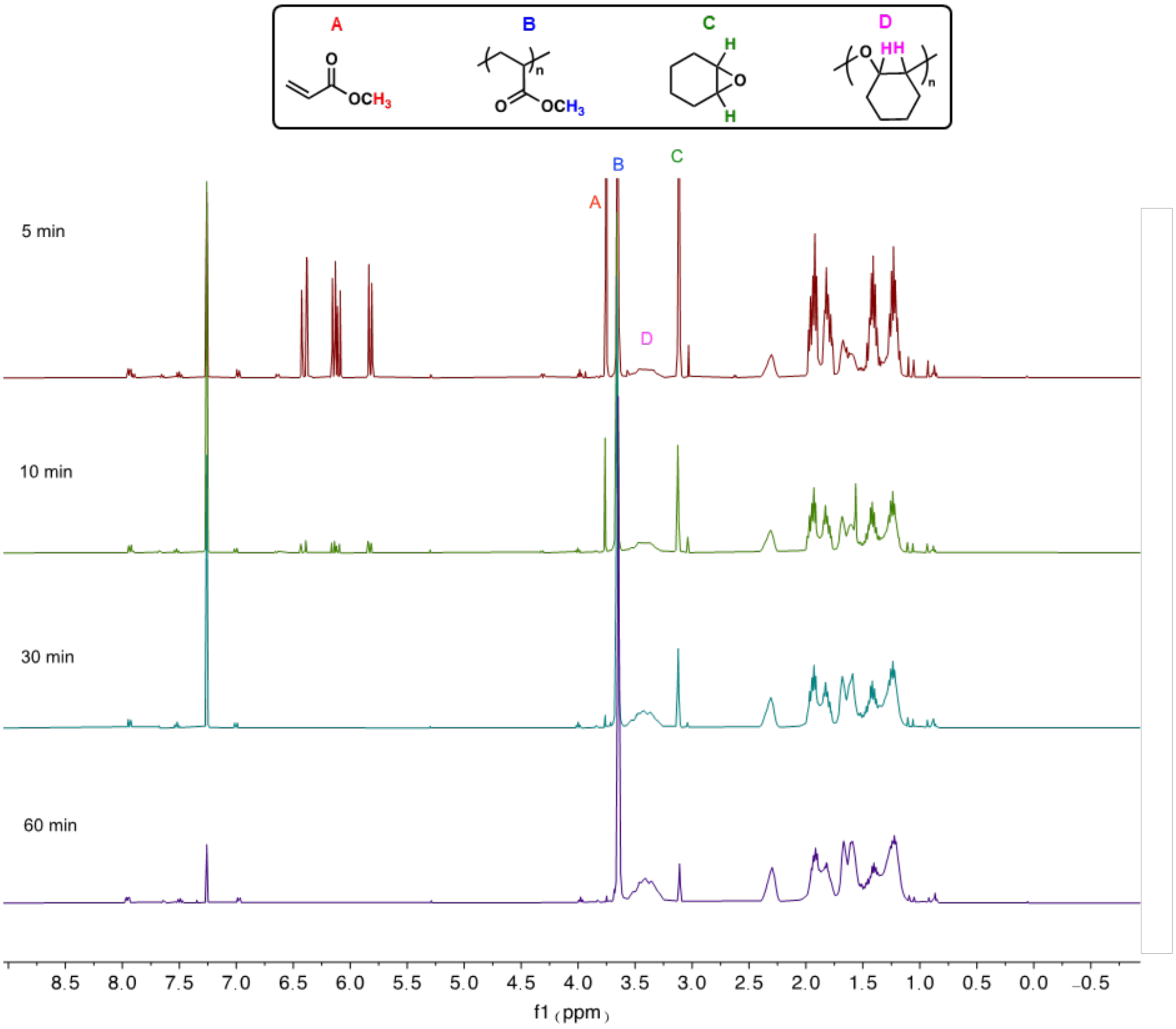


**Figure S4.** $^1$H NMR of MA-CHO polymerization conversions without photobuffer (450 nm, 0.14 mW/cm$^2$ irradiation).

***Procedures for Switching MA-CHO Polymerizations with Photobuffer TBACl***

**Low dose:** In a one-dram vial, CQ (1.65 mg, 0.01 mmol, 1 equiv), EDMAB (1.93 mg, 0.01 mmol, 1 equiv), TBACl (2.78 mg, 0.01 mmol, 1 equiv), and PAG-$SbF_6$ (12.9 mg, 0.02 mmol, 2 equiv) were added and dried under vacuum. After drying the vial was cycled between $N_2$ and vacuum three times. MA (0.1 ml, 1 mmol, 100 equiv) and CHO (0.1 ml, 1 mmol, 100 equiv) were added to the dram vial under $N_2$ atmosphere and irradiated with blue LED strips (450 nm) for 10 min and then kept in the dark. Aliquots for $^1$H NMR analysis were taken at 2 h, 4 h, 6 h, 8 h and 16 h and quenched with 0.1 mL of 5 vol% TEA in MeOH.

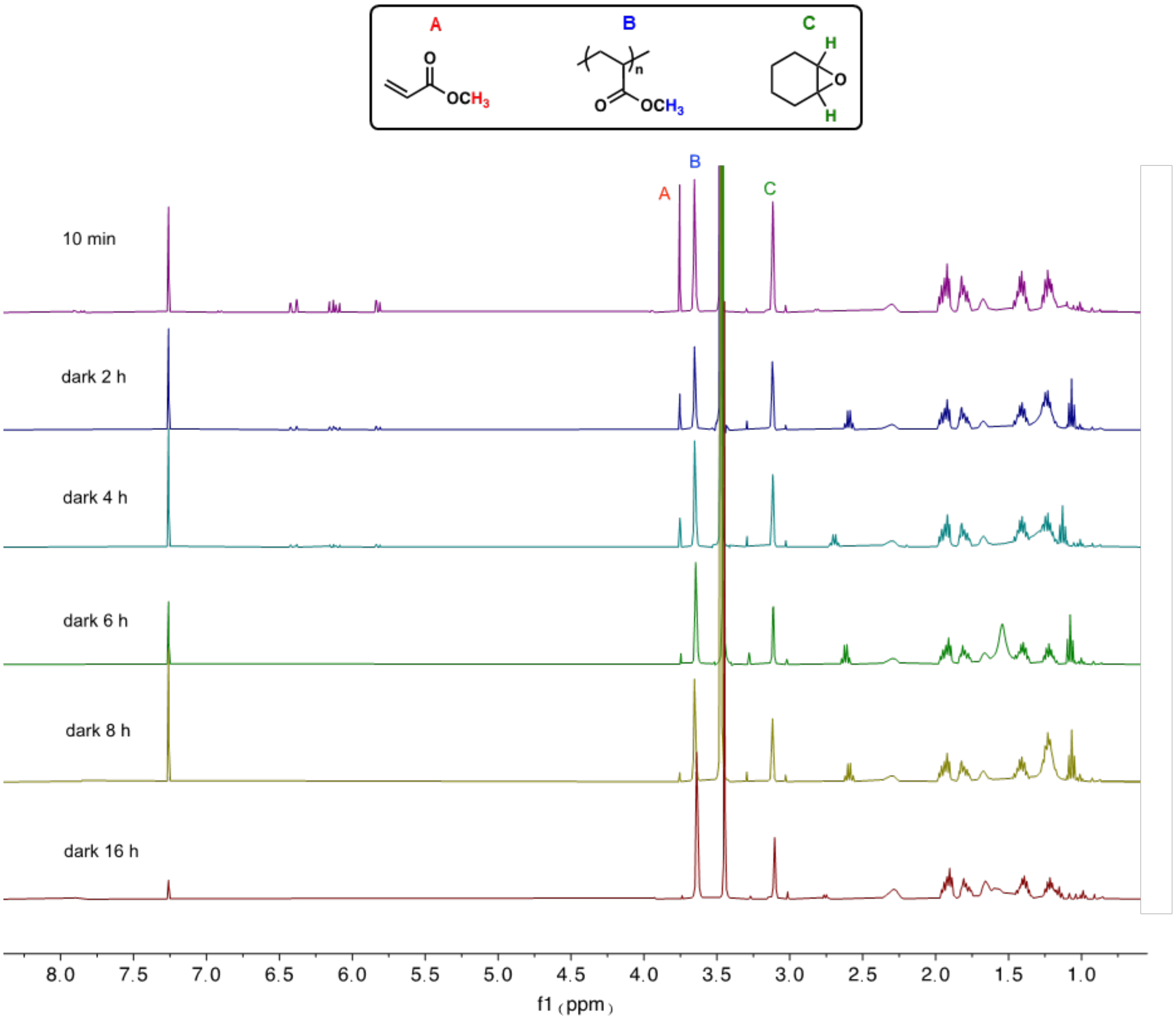


**Figure S5.** $^1$H NMR of MA-CHO polymerization conversions with 1 equiv photobuffer TBACl under a low light dose (10 mins irradiation, 450 nm, 0.14 mW/cm$^2$).

**High dose:** In a one-dram vial, CQ (1.65 mg, 0.01 mmol, 1 equiv), EDMAB (1.93 mg, 0.01 mmol, 1 equiv), TBACl (2.78 mg, 0.01 mmol, 1 equiv), and PAG-$SbF_6$ (12.9 mg, 0.02 mmol, 2 equiv) were added and dried under vacuum. After drying the vial was cycled between $N_2$ and vacuum three times. MA (0.1 ml, 1 mmol, 100 equiv) and CHO (0.1 ml, 1 mmol, 100 equiv) were added to the dram vial under $N_2$ atmosphere and irradiated with LED strips (450 nm) for an hour. Aliquots for $^1H$ NMR analysis were taken at 5 min, 10 min, 30 min, and 60 min of irradiation and quenched with 0.1 mL of 5 vol% TEA in MeOH.

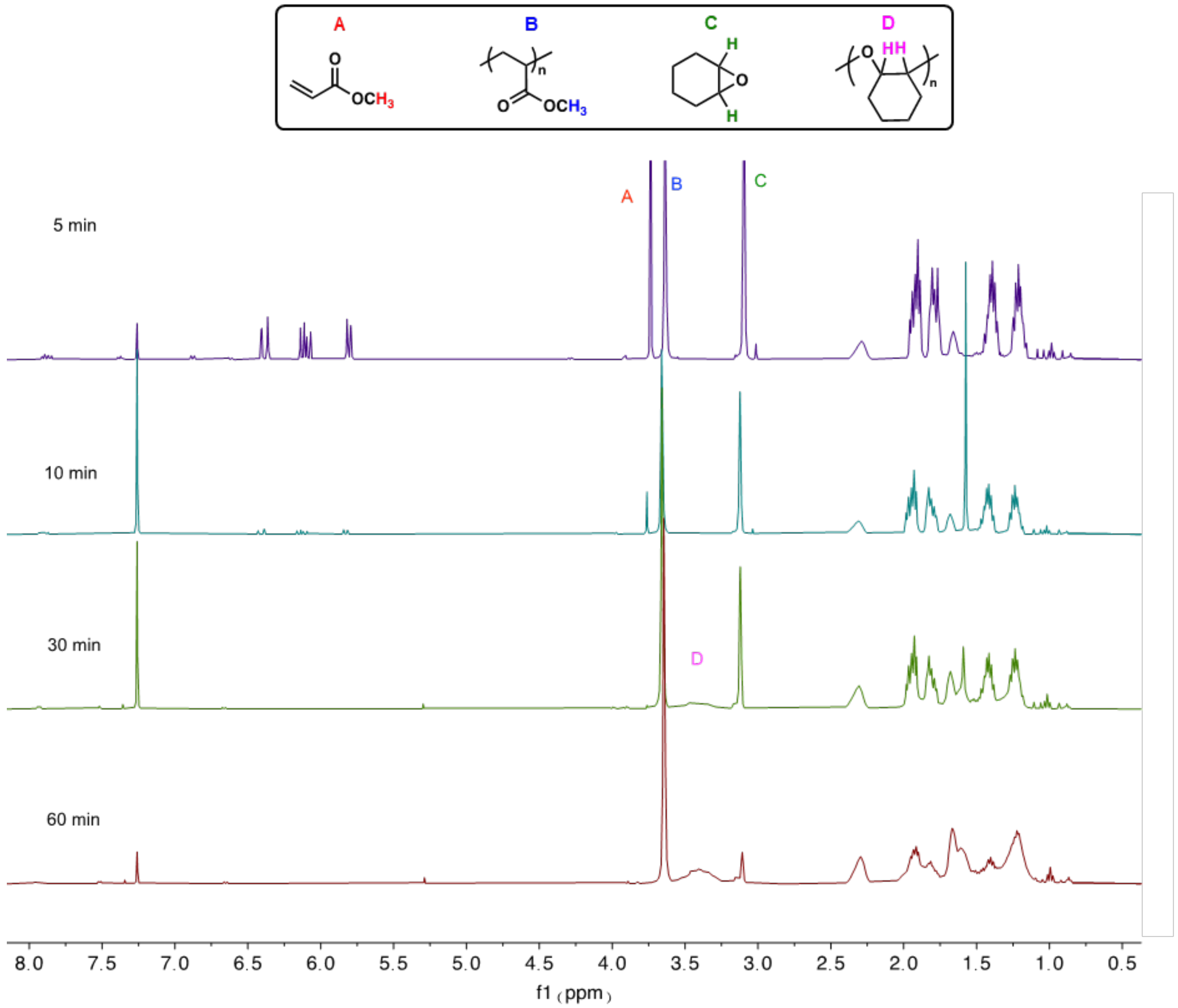


**Figure S6.** $^1H$ NMR of MA-CHO polymerization conversions with 1 equiv photobuffer TBACl under low light dose (60 mins irradiation, 450 nm, 0.14 mW/cm$^2$).

***Procedures for Control MMA-CHO Polymerizations without Photobuffer***

In a one-dram vial, CQ (1.65 mg, 0.01 mmol, 1 equiv), EDMAB (1.93 mg, 0.01 mmol, 1 equiv), and PAG-$SbF_6$ (12.9 mg, 0.02 mmol, 2 equiv) were added and dried under vacuum. After drying the vial was cycled between $N_2$ and vacuum three times. MMA (0.1 ml, 1 mmol, 100 equiv) and CHO (0.1 ml, 1 mmol, 100 equiv) were added to the dram vial under $N_2$ atmosphere and irradiated with blue LED strips (450 nm) for 30 minutes. Aliquots for $^1$H NMR analysis were taken at 5 min, 10 min, and 30 min light irradiation and quenched with 0.1 mL of 5 vol% TEA in MeOH.

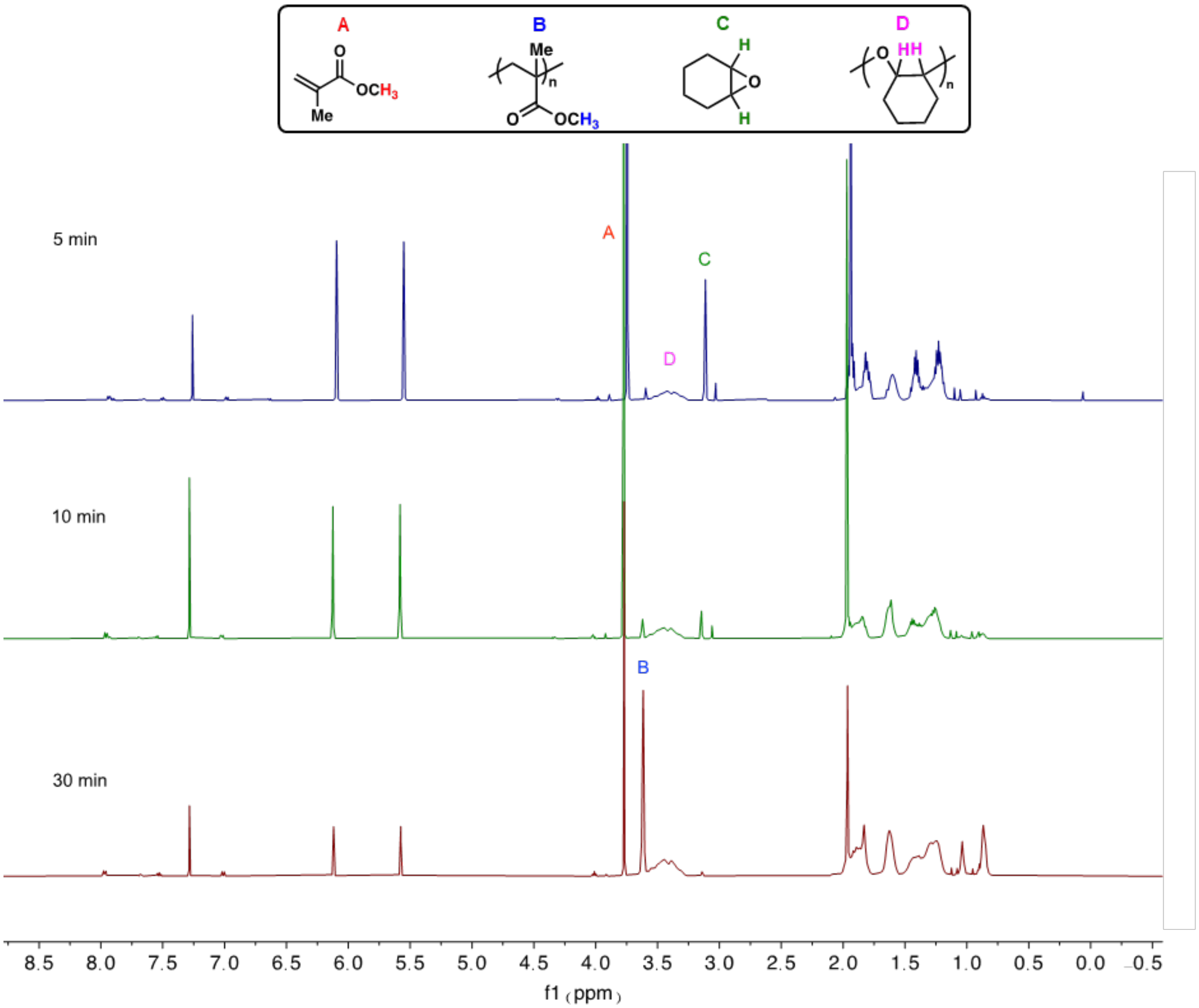


**Figure S7.** $^1$H NMR of MMA-CHO polymerization conversions without photobuffer (450 nm, 0.14 mW/cm$^2$ irradiation).

*Procedures for Switching MMA-CHO Polymerizations with Photobuffer TBACl*

**Low dose:** In a one-dram vial, CQ (1.65 mg, 0.01 mmol, 1 equiv), EDMAB (1.93 mg, 0.01 mmol, 1 equiv), TBACl (2.78 mg, 0.01 mmol, 1 equiv), and PAG-$SbF_6$ (12.9 mg, 0.02 mmol, 2 equiv) were added and dried under vacuum. After drying the vial was cycled between $N_2$ and vacuum three times. MMA (0.1 ml, 1 mmol, 100 equiv) and CHO (0.1 ml, 1 mmol, 100 equiv) were added to the dram vial under $N_2$ atmosphere and irradiated with blue LED strips (450 nm) for 60 min and then kept in the dark. Aliquots for $^1H$ NMR analysis were taken at 2 h, 4 h, 8 h and 16 h and quenched with 0.1 mL of 5 vol% TEA in MeOH.

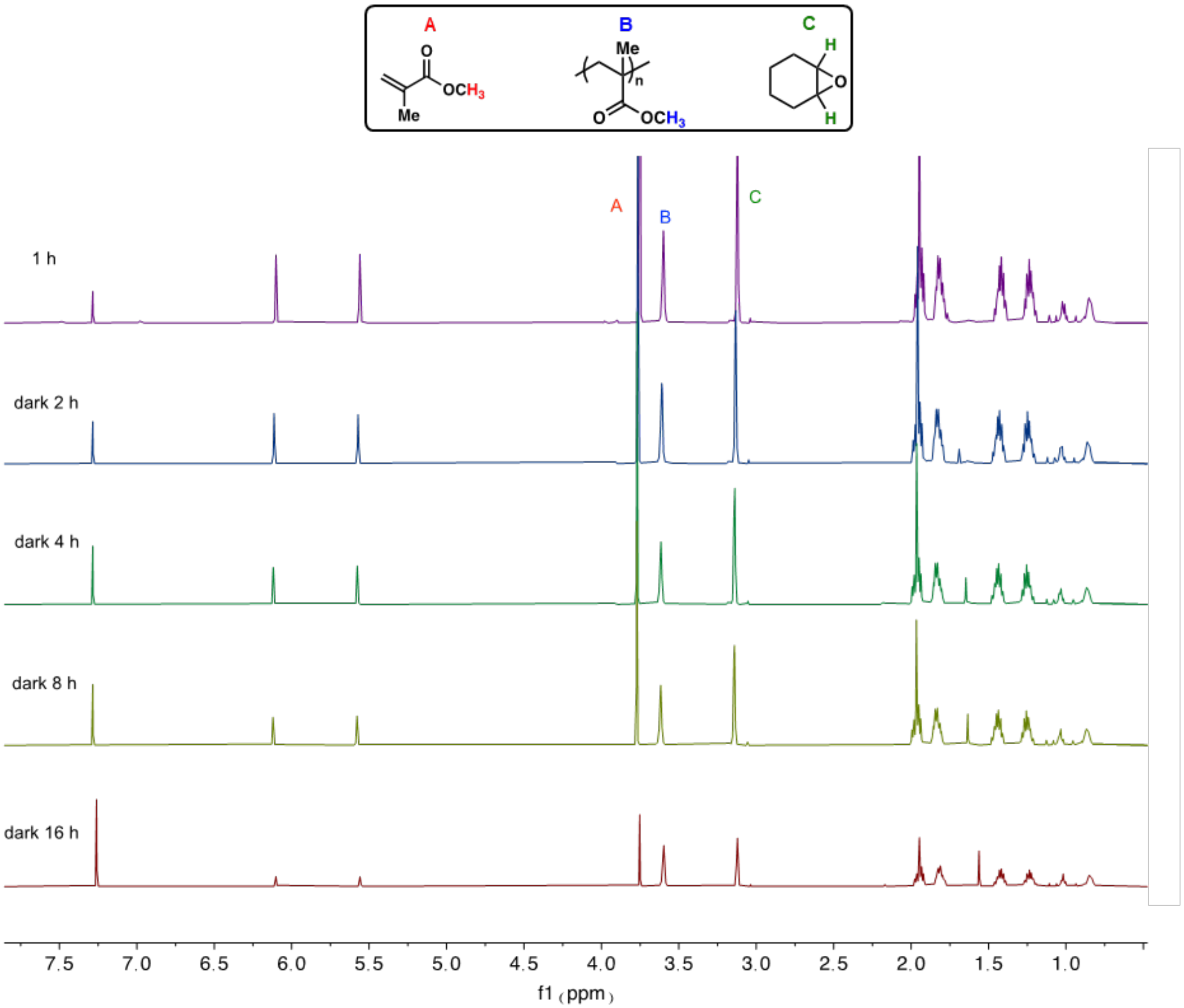


**Figure S8.** $^1H$ NMR of MMA-CHO polymerization conversions with 1 equiv photobuffer TBACl under low light dose (10 mins irradiation, 450 nm, 0.14 mW/cm$^2$).

**High dose:** In a one-dram vial, CQ (1.65 mg, 0.01 mmol, 1 equiv), EDMAB (1.93 mg, 0.01 mmol, 1 equiv), TBACl (2.78 mg, 0.01 mmol, 1 equiv), and PAG-$SbF_6$ (12.9 mg, 0.02 mmol, 2 equiv) were added and dried under vacuum. After drying the vial was cycled between $N_2$ and vacuum three times. MMA (0.1 ml, 1 mmol, 100 equiv) and CHO (0.1 ml, 1 mmol, 100 equiv) were added to the dram vial under $N_2$ atmosphere and irradiated with blue LED strips (450 nm) for two hours. Aliquots for $^1$H NMR analysis were taken at 5 min, 10 min, 30 min, 60 min, and 120 min light irradiation and quenched with 0.1 mL of 5 vol% TEA in MeOH.

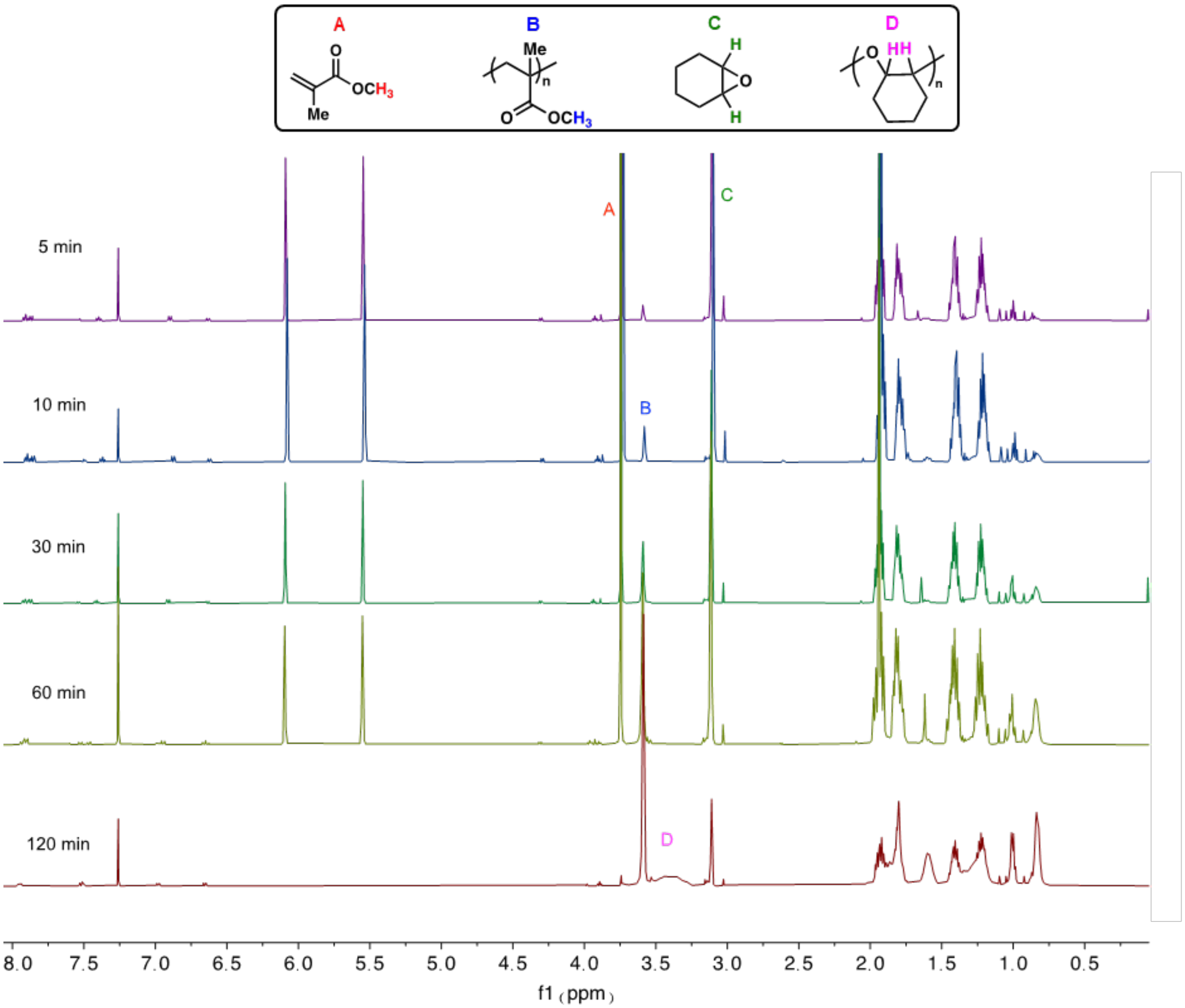


**Figure S9.** $^1$H NMR of MMA-CHO polymerization conversions with 1 equiv photobuffer TBACl under high light dose (120 mins irradiation, 450 nm, 0.14 mW/cm$^2$).

### *Procedures for Control MA-CL Polymerizations without Photobuffer*

In a one-dram vial, CQ (1.65 mg, 0.01 mmol, 1 equiv), EDMAB (1.93 mg, 0.01 mmol, 1 equiv), and PAG-$SbF_6$ (12.9 mg, 0.02 mmol, 2 equiv) were added and dried under vacuum. After drying the vial was cycled between $N_2$ and vacuum three times. MA (0.1 ml, 1 mmol, 100 equiv) and CL (0.11 ml, 1 mmol, 100 equiv) were added to the dram vial under $N_2$ atmosphere and irradiated with blue LED strips (450 nm) for 8 hours. Aliquots for $^1H$ NMR analysis were taken at 5 min, 10 min, 30 min, 60 min, 2 h, 4 h, and 8 h light irradiation and quenched with 0.1 mL of 5 vol% TEA in MeOH.

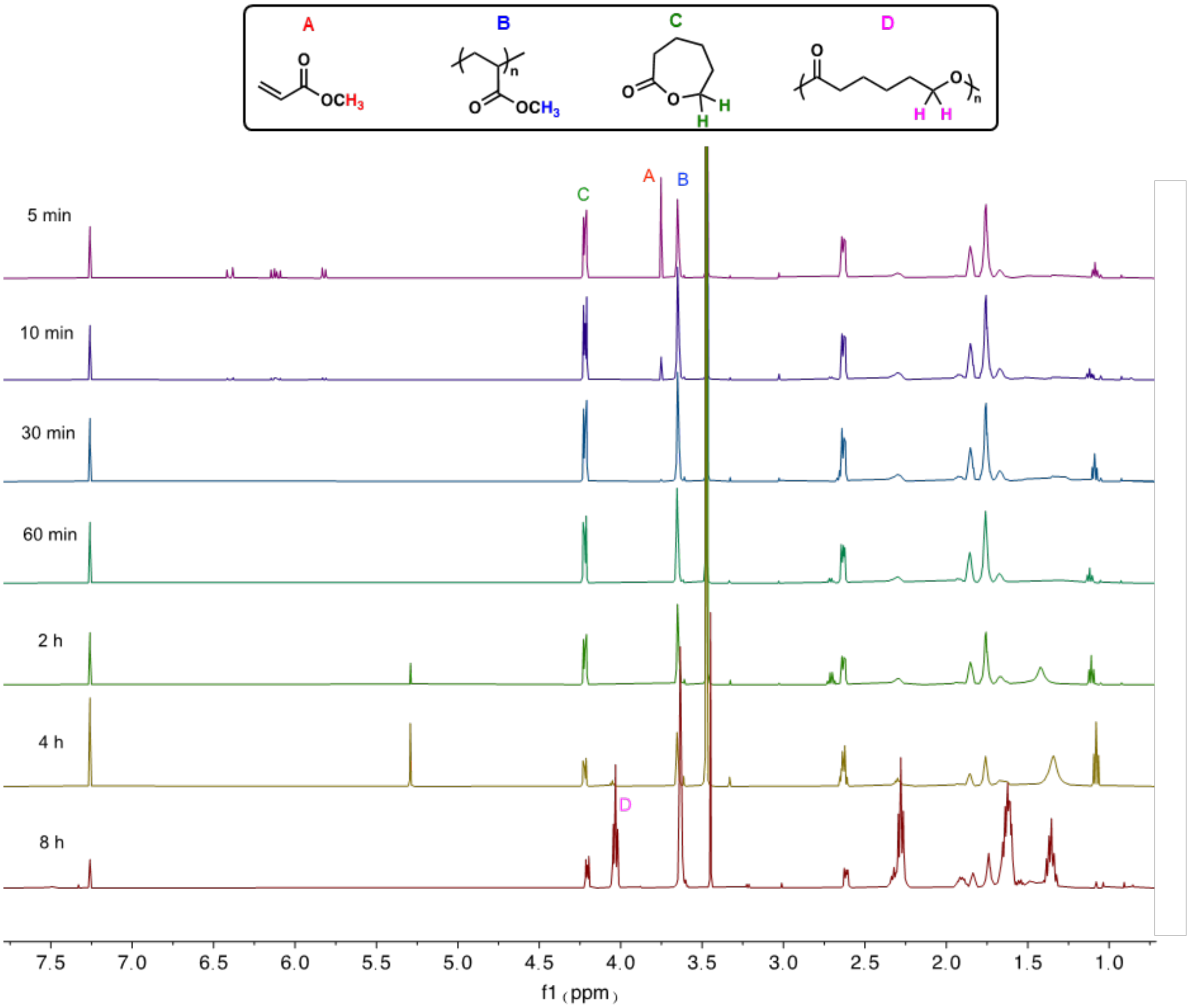


**Figure S10**. $^1H$ NMR of MA-CL polymerization conversions without photobuffer (450 nm, 0.14 $mW/cm^2$ irradiation).

*Procedures for Switching MA-CL Polymerizations with Photobuffer TBACl*

**Low dose:** In a one-dram vial, CQ (1.65 mg, 0.01 mmol, 1 equiv), EDMAB (1.93 mg, 0.01 mmol, 1 equiv), and PAG-$SbF_6$ (12.9 mg, 0.02 mmol, 2 equiv) were added and dried under vacuum. After drying the vial was cycled between $N_2$ and vacuum three times. MA (0.1 ml, 1 mmol, 100 equiv) and CL (0.11 ml, 1 mmol, 100 equiv) were added to the dram vial under $N_2$ atmosphere and irradiated with blue LED strips (450 nm) for 5 min and then kept in dark. Aliquots for $^1H$ NMR analysis were taken at 10 min, 30 min, 1 h, 2 h, 4 h, 8 h, and 16 h and quenched with 0.1 mL of 5 vol% TEA in MeOH.

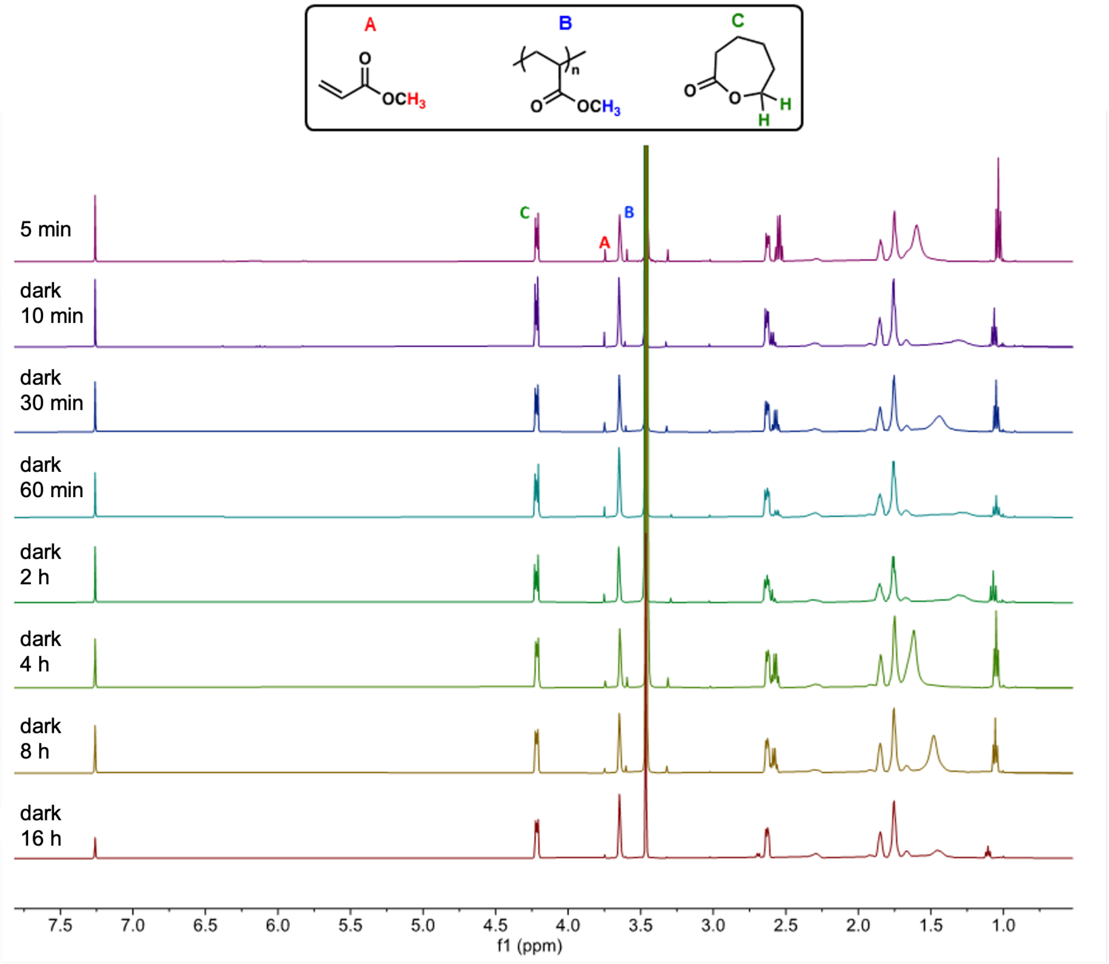


**Figure S11.** $^1H$ NMR of MA-CL polymerization conversions with 1 equiv photobuffer TBACl under low light dose (5 mins irradiation, 450 nm, 0.14 mW/cm$^2$).

**High dose:** In a one-dram vial, CQ (1.65 mg, 0.01 mmol, 1 equiv), EDMAB (1.93 mg, 0.01 mmol, 1 equiv), TBACl (2.78 mg, 0.01 mmol, 1 equiv), and PAG-$SbF_6$ (12.9 mg, 0.02 mmol, 2 equiv) were added and dried under vacuum. After drying the vial was cycled between $N_2$ and vacuum three times. MA (0.1 ml, 1 mmol, 100 equiv) and CL (0.1 ml, 1 mmol, 100 equiv) were added to the dram vial under $N_2$ atmosphere and irradiated with blue LED strips (450 nm) for 16 hours. Aliquots for $^1$H NMR analysis were taken at 5 min, 10 min, 30 min, 1 h, 6 h, 8 h, and 16 h light irradiation and quenched with 0.1 mL of 5 vol% TEA in MeOH.

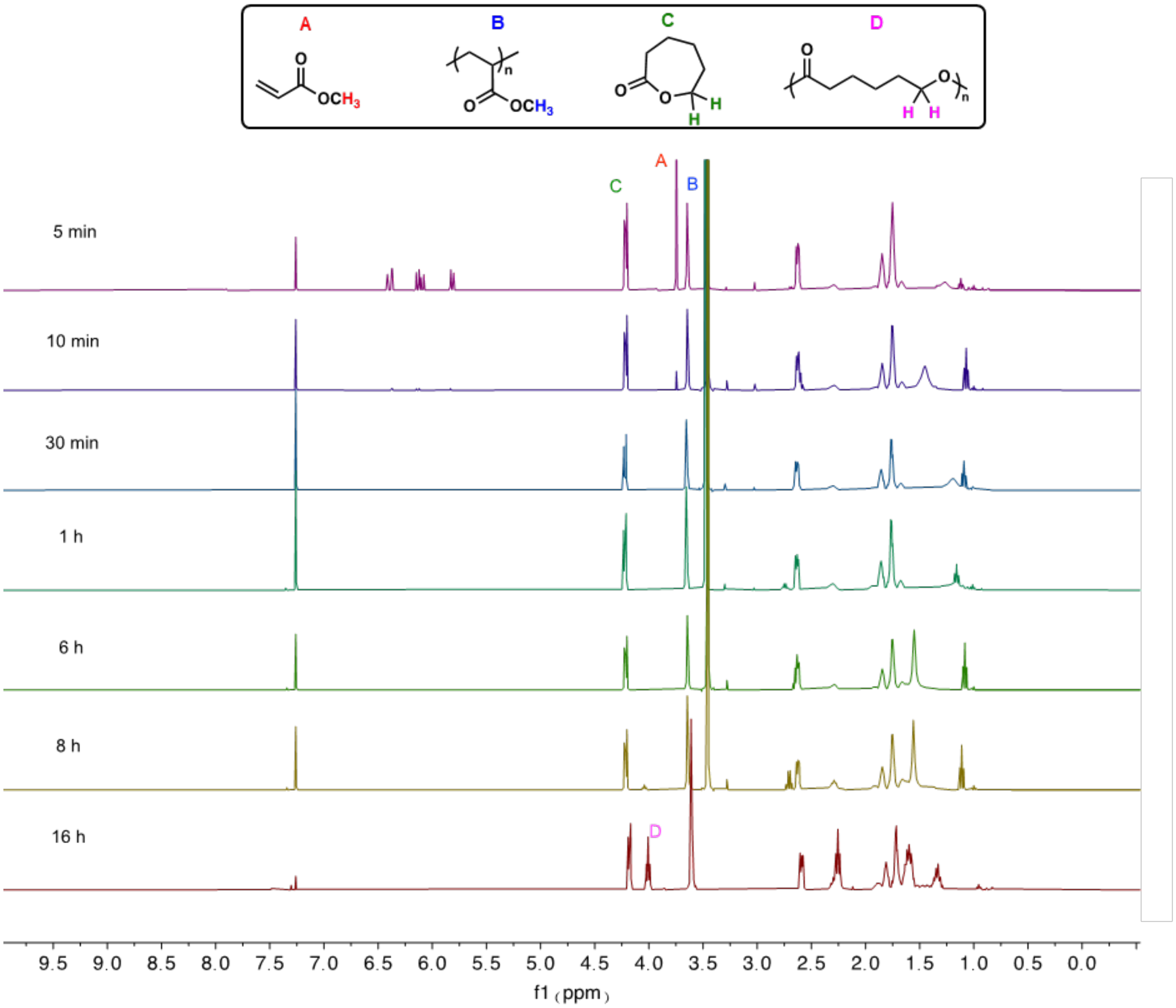


**Figure S12.** $^1$H NMR of MA-CL polymerization conversions with 1 equiv photobuffer TBACl under high light dose (16 h irradiation, 450 nm, 0.14 mW/cm$^2$).

### *Procedures for Control MA-DXL Polymerizations without Photobuffer*

In a one-dram vial, CQ (1.65 mg, 0.01 mmol, 1 equiv), EDMAB (1.93 mg, 0.01 mmol, 1 equiv), and PAG-$SbF_6$ (12.9 mg, 0.02 mmol, 2 equiv) were added and dried under vacuum. After drying the vial was cycled between $N_2$ and vacuum three times. MA (0.1 ml, 1 mmol, 100 equiv) and DXL (0.07 ml, 1 mmol, 100 equiv) were added to the dram vial under $N_2$ atmosphere and irradiated with blue LED strips (450 nm) for 8 hours. Aliquots for $^1$H NMR analysis were taken at 5 min, 10 min, 30 min, 1 h, 2 h, 4 h, and 8 h light irradiation and quenched with 0.1 mL of 5 vol% TEA in MeOH.

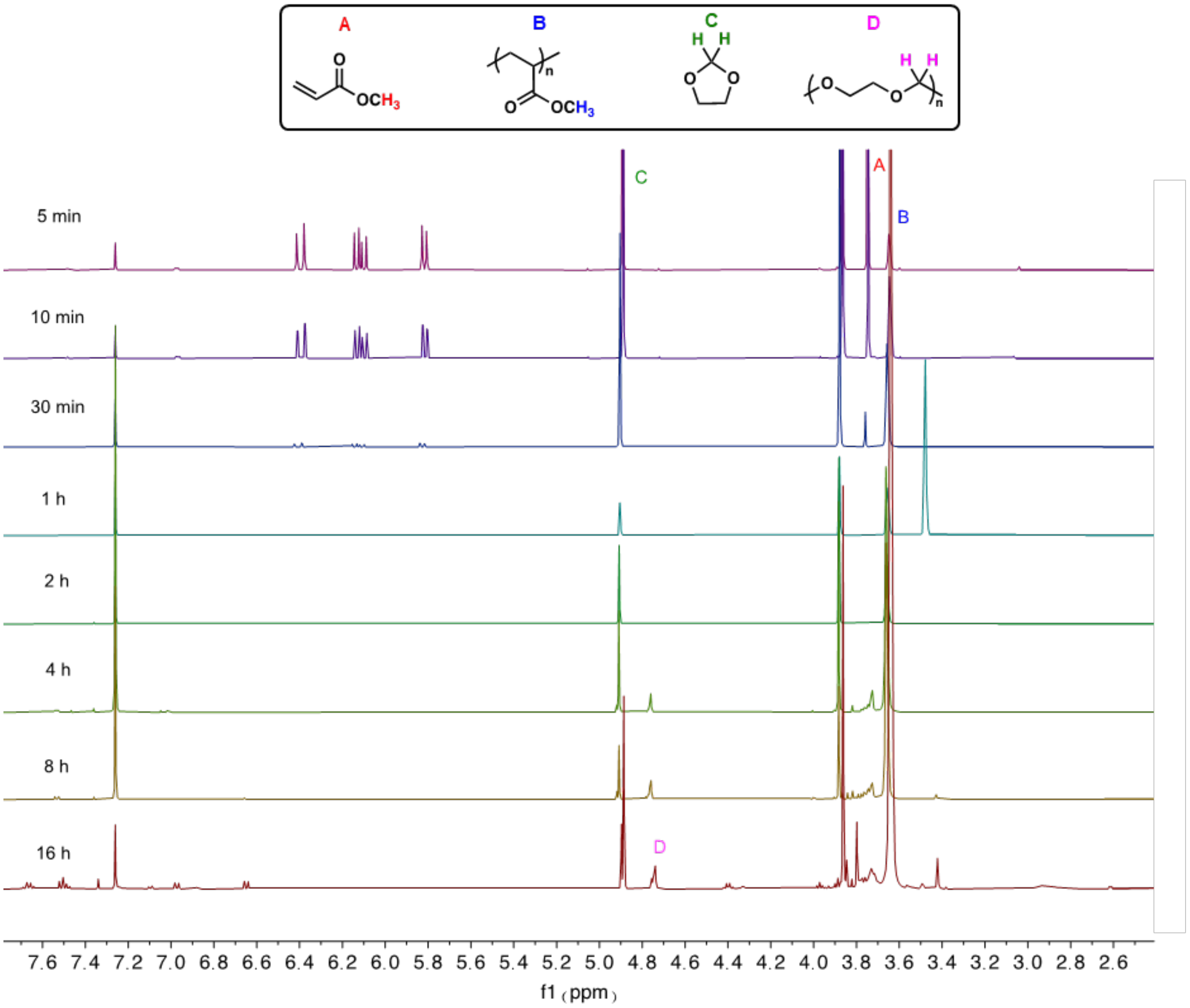


**Figure S13.** $^1$H NMR of MA-DXL polymerization conversions without photobuffer (450 nm, 0.14 mW/cm$^2$ irradiation).

*Procedures for Switching MA-DXL Polymerizations with Photobuffer TBACl*

**Low dose:** In a one-dram vial, CQ (1.65 mg, 0.01 mmol, 1 equiv), EDMAB (1.93 mg, 0.01 mmol, 1 equiv), TBACl (2.78 mg, 0.01 mmol, 1 equiv), and PAG-$SbF_6$ (12.9 mg, 0.02 mmol, 2 equiv) were added and dried under vacuum. After drying the vial was cycled between $N_2$ and vacuum three times. MA (0.1 ml, 1 mmol, 100 equiv) and DXL (0.07 ml, 1 mmol, 100 equiv) were added to the dram vial under $N_2$ atmosphere and irradiated with blue LED strips (450 nm) for 10 min and then kept in dark. Aliquots for $^1$H NMR analysis were taken at 30 min, 1 h, 2 h, 8 h and 16 h and quenched with 0.1 mL of 5 vol% TEA in MeOH.

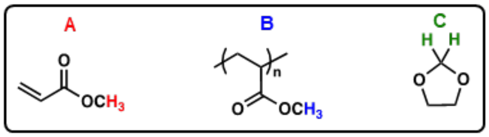


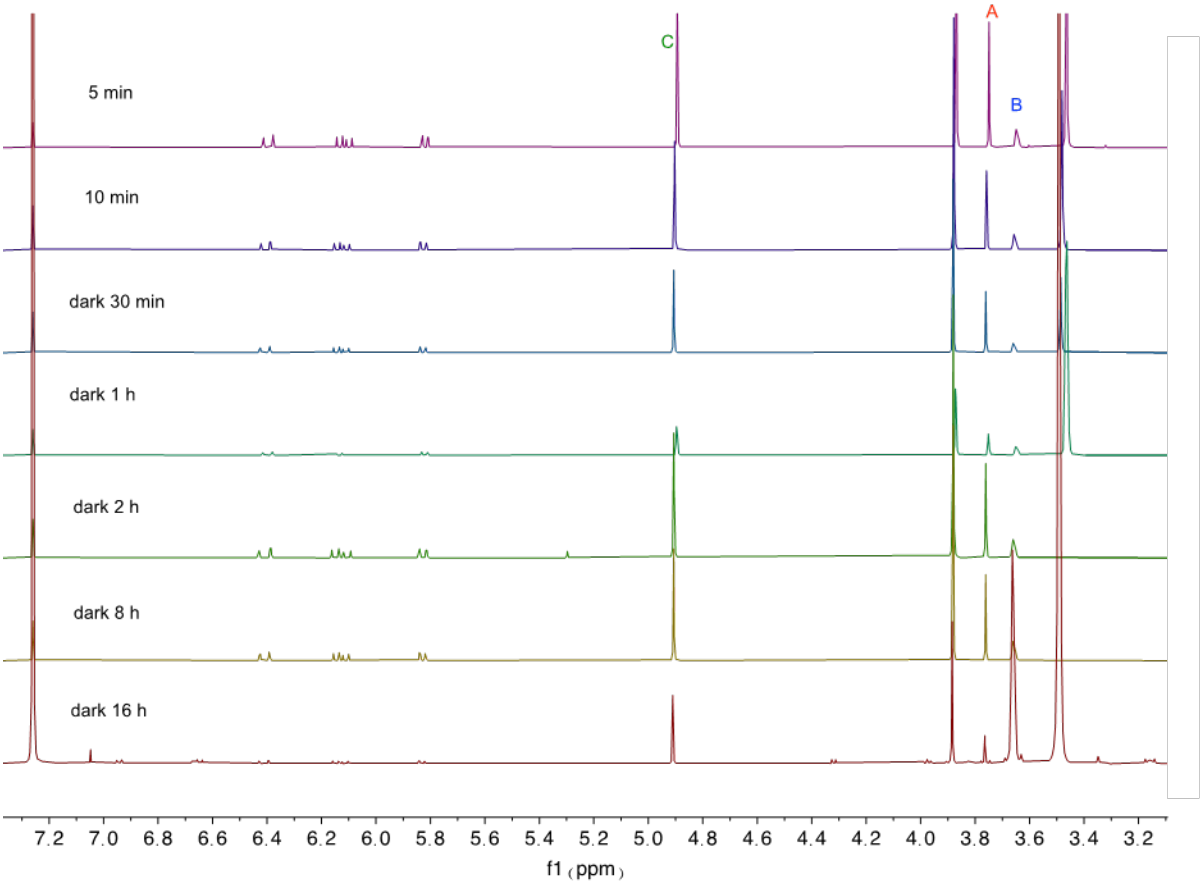


**Figure S14**. $^1$H NMR of MA-DXL polymerization conversions with 1 equiv photobuffer TBACl low light dose (10 mins irradiation, 450 nm, 0.14 mW/cm$^2$).

**High dose:** In a one-dram vial, CQ (1.65 mg, 0.01 mmol, 1 equiv), EDMAB (1.93 mg, 0.01 mmol, 1 equiv), TBACl (2.78 mg, 0.01 mmol, 1 equiv), and PAG-$SbF_6$ (12.9 mg, 0.02 mmol, 2 equiv) were added and dried under vacuum. After drying the vial was cycled between $N_2$ and vacuum three times. MA (0.1 ml, 1 mmol, 100 equiv) and DXL (0.07 ml, 1 mmol, 100 equiv) were added to the dram vial under $N_2$ atmosphere and moved to blue LED strips (450 nm) for 16 hours. Aliquots for $^1H$ NMR analysis were taken at 5 min, 10 min, 30 min, 1 h, 2 h, 4 h, 8 h, and 16 h light irradiation and quenched with 0.1 mL of 5 vol% TEA in MeOH.

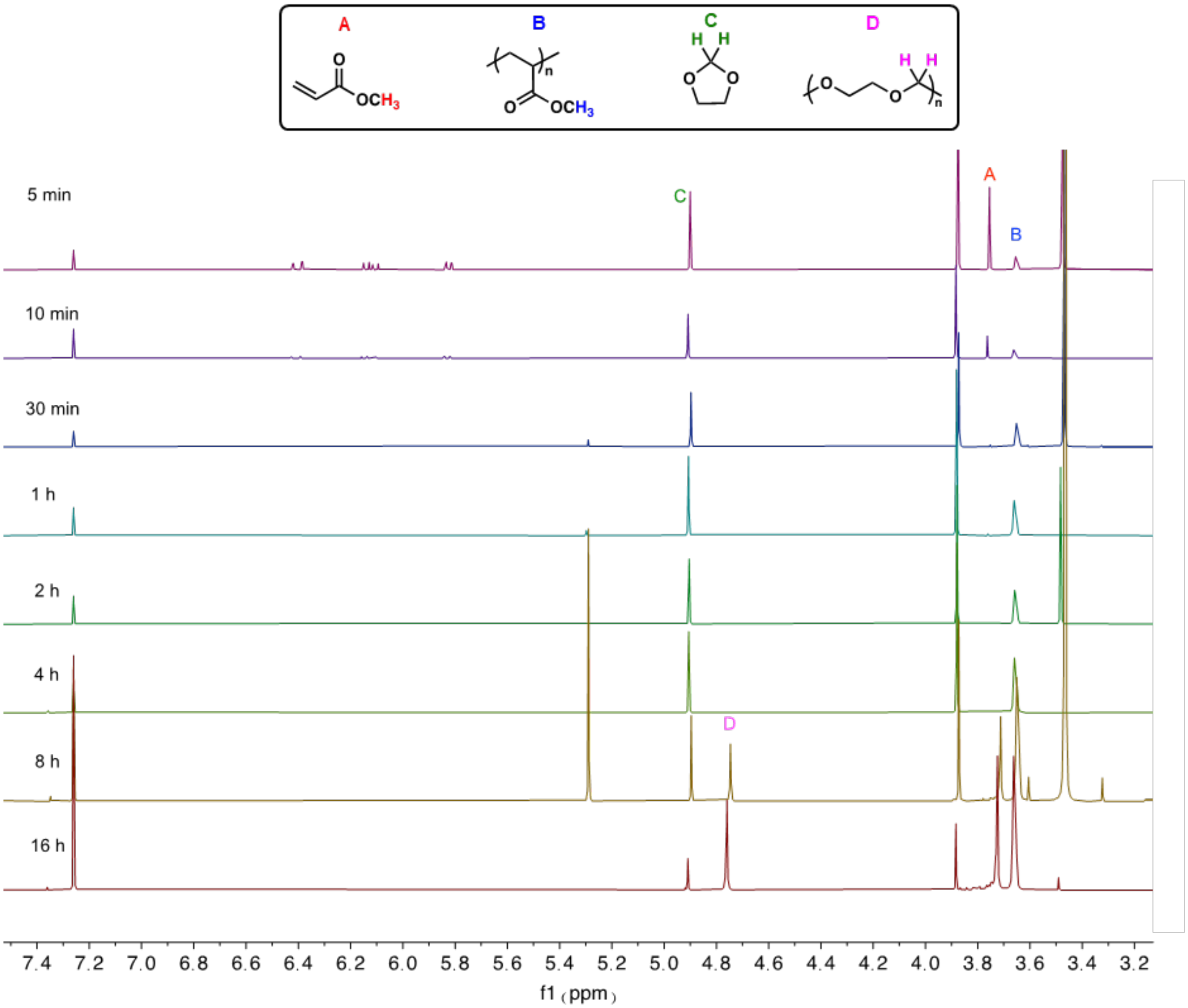


**Figure S15.** $^1H$ NMR of MA-DXL polymerization conversions with 1 equiv photobuffer TBACl under low light dose (16 h irradiation, 450 nm, 0.14 mW/cm$^2$).

***Unsuccessful Switching MA-CHO Polymerizations with Pyridine Photobuffer***

**Low dose:** In a one-dram vial, CQ (1.65 mg, 0.01 mmol, 1 equiv), EDMAB (1.93 mg, 0.01 mmol, 1 equiv), and PAG-$SbF_6$ (12.9 mg, 0.02 mmol, 2 equiv) were added and dried under vacuum. After drying the vial was cycled between $N_2$ and vacuum three times. MA (0.1 ml, 1 mmol, 100 equiv), CHO (0.1 ml, 1 mmol, 100 equiv), and pyridine (0.8 µL, 0.01 mmol, 1 equiv) were added to the dram vial under $N_2$ atmosphere and irradiated with blue LED strips (450 nm) for 10 min and then kept in the dark. Aliquots for $^1$H NMR analysis were taken at 2 h, 4 h, 6 h, 8 h and 16 h and quenched with 0.1 mL of 5 vol% TEA in MeOH.

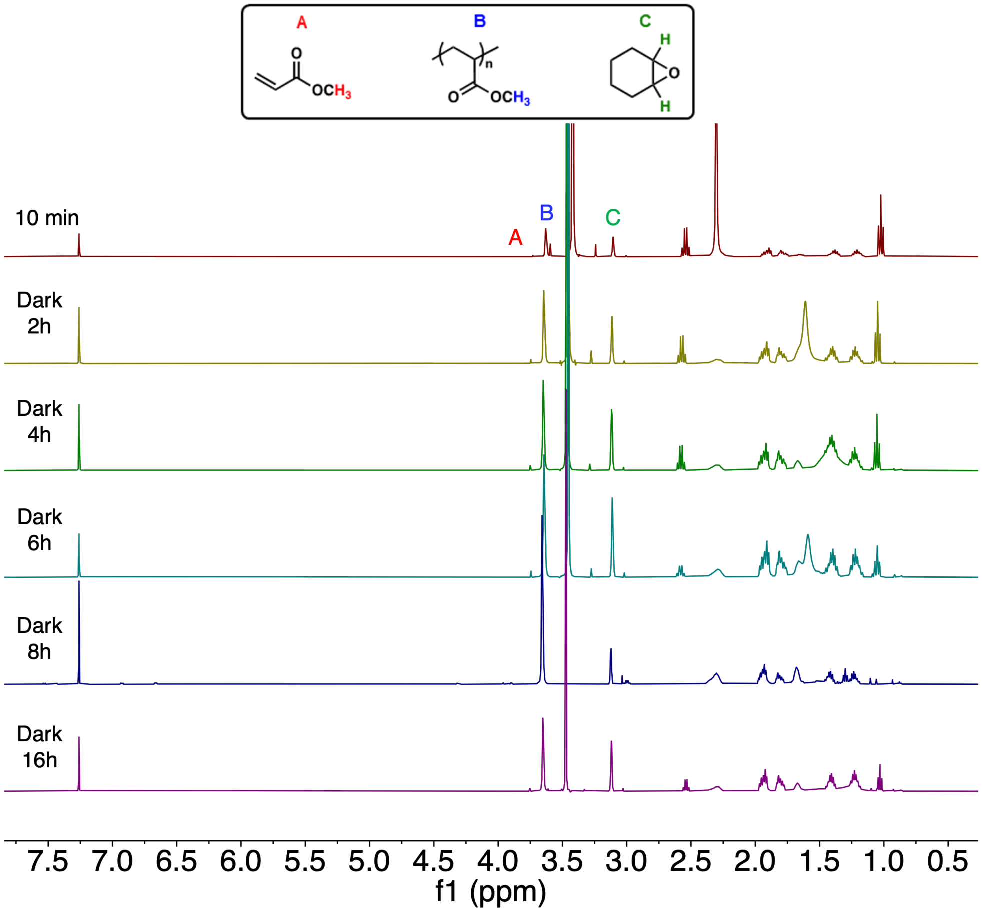


**Figure S16.** $^1$H NMR of MA-CHO polymerization conversions with 1 equiv photobuffer pyridine under low light dose (10 min irradiation, 450 nm, 0.14 mW/cm$^2$).

**High dose:** In a one-dram vial, CQ (1.65 mg, 0.01 mmol, 1 equiv), EDMAB (1.93 mg, 0.01 mmol, 1 equiv), and PAG-$SbF_6$ (12.9 mg, 0.02 mmol, 2 equiv) were added and dried under vacuum. After drying the vial was cycled between $N_2$ and vacuum three times. MA (0.1 ml, 1 mmol, 100 equiv), CHO (0.1 ml, 1 mmol, 100 equiv), and pyridine (0.8 µL, 0.01 mmol, 1 equiv) were added to the dram vial under $N_2$ atmosphere and irradiated with LED strips (450 nm) for an hour. Aliquots for $^1H$ NMR analysis were taken at 5 min, 10 min, 30 min, and 60 min of irradiation and quenched with 0.1 mL of 5 vol% TEA in MeOH.

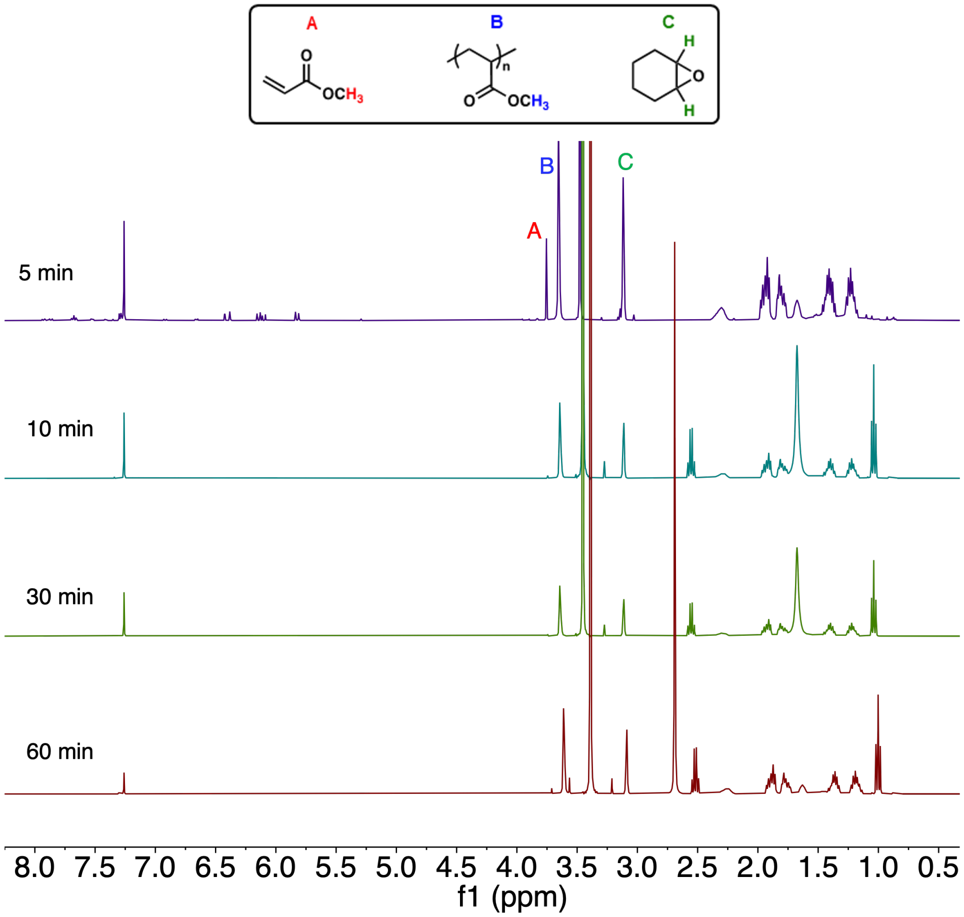


**Figure S17.** $^1H$ NMR of MA-CHO polymerization conversions with 1 equiv photobuffer pyridine under high light dose (60 min irradiation, 450 nm, 0.14 mW/cm$^2$). Only radical polymerization of MA proceeded, and no cationic polymerization of CHO was observed, even after 60 minutes of irradiation. When the less basic TBACl was used as photobuffer, CHO polymerization was observed after 30 mins (Fig S6). These results indicate that pyridine is too basic for cationic polymerization and is not a suitable photobuffer.

***General Procedure for Making Acrylate-Epoxide Thermoset with Low Light Dose***

In a nitrogen filled glovebox, a silicone mold was charged with CQ (23.2 mg, 0.14 mmol, 1 equiv), EDMAB (27 mg, 0.14 mmol, 1 equiv), TBACl (19.5 mg, 0.07 mmol, 0.5 equiv), PAG-$SbF_6$ (90.4 mg, 0.14 mmol, 1 equiv), MA (2.02 ml, 22.4 mmol, 160 equiv), TEGDA (0.06 ml, 0.224 mmol, 1.6 equiv), CHO (0.26 ml, 2.8 mmol, 20 equiv), ECC (0.62 ml, 2.8 mmol, 20 equiv), and OXAA (0.2 ml, 0.98 mmol, 7 equiv). The reaction solution was mixed until homogenous and capped with a Petri dish before removal from the glovebox. Then the reaction mixture was irradiated with a blue Kessil lamp (456 nm, 25% light intensity, 23 mW/cm$^2$) while cooling with blown compressed air. After irradiation for 30 s, the reaction was opened to air. The crosslinked films were swelled in 1:1 isopropanol: acetone overnight and dried in vacuum oven at 50 °C overnight to remove any residue.

***General Procedure for Making Acrylate-Epoxide Thermoset with High Light Dose***

In a nitrogen filled glovebox, a silicone mold was charged with CQ (23.2 mg, 0.14 mmol, 1 equiv), EDMAB (27 mg, 0.14 mmol, 1 equiv), TBACl (19.5 mg, 0.07 mmol, 0.5 equiv), PAG-$SbF_6$ (90.4 mg, 0.14 mmol, 1 equiv), MA (2.02 ml, 22.4 mmol, 160 equiv), TEGDA (0.06 ml, 0.224 mmol, 1.6 equiv), CHO (0.26 ml, 2.8 mmol, 20 equiv), ECC (0.62 ml, 2.8 mmol, 20 equiv), and OXAA (0.2 ml, 0.98 mmol, 7 equiv). The reaction solution was mixed until homogenous and capped with a Petri dish before removal from the glovebox. Then the reaction mixture was irradiated with a blue Kessil lamp (456 nm, 25% light intensity, 23 mW/cm$^2$) while cooling with blown compressed air. After irradiation for 1 hour, the reaction was opened to air. The crosslinked films were dried in air overnight before mechanical testing.

***General Procedure for Spatial Control of Thermoset Properties***

In a nitrogen filled glovebox, an oven-dried flat bottom, cylindrical reaction vessel was charged with CQ (25.7 mg, 0.16 mmol, 1 equiv), EDMAB (30 mg, 0.16 mmol, 1 equiv), TBACl (21.6 mg, 0.08 mmol, 0.5 equiv), PAG-$SbF_6$ (100.4 mg, 0.16 mmol, 1 equiv), MA (2.24 ml, 25.6 mmol, 160 equiv), TEGDA (0.067 ml, 0.256 mmol, 1.6 equiv), CHO (0.3 ml, 3.2 mmol, 20 equiv), ECC (0.68 ml, 3.2 mmol, 20 equiv), and OXAA (0.22 ml, 1.12 mmol, 7 equiv). The vessel was sealed with a clamp and removed from glovebox, then irradiated with a blue Kessil lamp (456 nm, 25% light intensity, 23 mW/cm$^2$) while cooling with blown compressed air. After 10 s, the vessel was further irradiated with a blue Thorlabs collimated LED (455 nm, 500 mW, 86 mW/cm$^2$) equipped with two convex lenses and a photomask to pattern the polymer film. After 1 hour irradiation, the vessel was opened to air. The crosslinked films were dried in air overnight before mechanical testing.

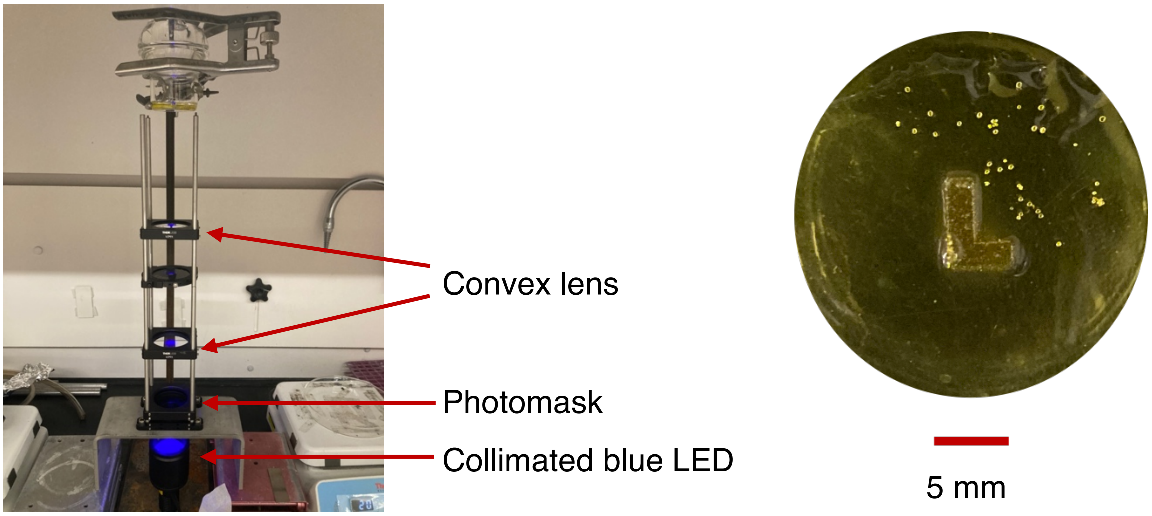


**Figure S18.** Left: reaction setup for spatial control of thermoset properties. Right: A photo patterned crosslinked acrylate-epoxide polymer film.

**Table S1.** Resin Components in Figure 3C

**100:1 MA: TEGDA**

| Reagents | Equivalents | mmol | Volume |
|---|---|---|---|
| CQ | 1 | 0.14 | 23.2 mg |
| EDMAB | 1 | 0.14 | 27 mg |
| TBACl | 0.5 | 0.07 | 19.5 mg |
| PAG-$SbF_6$ | 1 | 0.14 | 90.4 mg |
| MA | 160 | 22.4 | 2.02 ml |
| TEGDA | 1.6 | 0.224 | 0.06 ml |
| CHO | 20 | 2.8 | 0.26 ml |
| ECC | 20 | 2.8 | 0.62 ml |
| OXAA | 7 | 0.98 | 0.2 ml |

**90:10 MA: TEGDA**

| Reagents | Equivalents | mmol | Volume |
|---|---|---|---|
| CQ | 1 | 0.14 | 23.2 mg |
| EDMAB | 1 | 0.14 | 27 mg |
| TBACl | 0.5 | 0.07 | 19.5 mg |
| PAG-$SbF_6$ | 1 | 0.14 | 90.4 mg |
| MA | 90 | 12.6 | 1.14 ml |
| TEGDA | 10 | 1.4 | 0.38 ml |
| CHO | 20 | 2.8 | 0.26 ml |
| ECC | 20 | 2.8 | 0.62 ml |
| OXAA | 7 | 0.98 | 0.2 ml |

**70:30 MA: TEGDA**

| Reagents | Equivalents | mmol | Volume |
|---|---|---|---|
| CQ | 1 | 0.14 | 23.2 mg |
| EDMAB | 1 | 0.14 | 27 mg |
| TBACl | 0.5 | 0.07 | 19.5 mg |
| PAG-$SbF_6$ | 1 | 0.14 | 90.4 mg |
| MA | 70 | 26.1 | 0.88 ml |
| TEGDA | 30 | 4.2 | 1.13 ml |
| CHO | 20 | 2.8 | 0.26 ml |
| ECC | 20 | 2.8 | 0.62 ml |
| OXAA | 7 | 0.98 | 0.2 ml |

**Table S2.** Resin Components in Figure 3D

**5:5 CHO: ECC**

| Reagents | Equivalents | mmol | Volume |
|---|---|---|---|
| CQ | 1 | 0.14 | 23.2 mg |
| EDMAB | 1 | 0.14 | 27 mg |
| TBACl | 0.5 | 0.07 | 19.5 mg |
| PAG-$SbF_6$ | 1 | 0.14 | 90.4 mg |
| MA | 160 | 22.4 | 2.02 ml |
| TEGDA | 1.6 | 0.224 | 0.06 ml |
| CHO | 5 | 0.7 | 0.06 ml |
| ECC | 5 | 0.7 | 0.16 ml |
| OXAA | 7 | 0.98 | 0.2 ml |

**10:10 CHO: ECC**

| Reagents | Equivalents | mmol | Volume |
|---|---|---|---|
| CQ | 1 | 0.14 | 23.2 mg |
| EDMAB | 1 | 0.14 | 27 mg |
| TBACl | 0.5 | 0.07 | 19.5 mg |
| PAG-$SbF_6$ | 1 | 0.14 | 90.4 mg |
| MA | 160 | 22.4 | 2.02 ml |
| TEGDA | 1.6 | 0.224 | 0.06 ml |
| CHO | 10 | 1.4 | 0.13 ml |
| ECC | 10 | 1.4 | 0.31 ml |
| OXAA | 7 | 0.98 | 0.2 ml |

**20:20 CHO: ECC**

| Reagents | Equivalents | mmol | Volume |
|---|---|---|---|
| CQ | 1 | 0.14 | 23.2 mg |
| EDMAB | 1 | 0.14 | 27 mg |
| TBACl | 0.5 | 0.07 | 19.5 mg |
| PAG-$SbF_6$ | 1 | 0.14 | 90.4 mg |
| MA | 160 | 22.4 | 2.02 ml |
| TEGDA | 1.6 | 0.224 | 0.06 ml |
| CHO | 20 | 2.8 | 0.26 ml |
| ECC | 20 | 2.8 | 0.62 ml |
| OXAA | 7 | 0.98 | 0.2 ml |

**40:40 CHO: ECC**

| Reagents | Equivalents | mmol | Volume |
|---|---|---|---|
| CQ | 1 | 0.14 | 23.2 mg |
| EDMAB | 1 | 0.14 | 27 mg |
| TBACl | 0.5 | 0.07 | 19.5 mg |
| PAG-$SbF_6$ | 1 | 0.14 | 90.4 mg |
| MA | 160 | 22.4 | 2.02 ml |
| TEGDA | 1.6 | 0.224 | 0.06 ml |
| CHO | 40 | 5.6 | 0.52 ml |
| ECC | 40 | 5.6 | 1.24 ml |
| OXAA | 7 | 0.98 | 0.2 ml |

**Table S3.** Resin Components in Figure 3E

**Photobuffer TBACl**

| Reagents | Equivalents | mmol | Volume |
|---|---|---|---|
| CQ | 1 | 0.14 | 23.2 mg |
| EDMAB | 1 | 0.14 | 27 mg |
| TBACl | 0.5 | 0.07 | 19.5 mg |
| PAG-$SbF_6$ | 1 | 0.14 | 90.4 mg |
| MA | 160 | 22.4 | 2.02 ml |
| TEGDA | 1.6 | 0.224 | 0.06 ml |
| CHO | 20 | 2.8 | 0.26 ml |
| ECC | 20 | 2.8 | 0.62 ml |
| OXAA | 7 | 0.98 | 0.2 ml |

**Photobuffer TBAPCCP**

| Reagents | Equivalents | mmol | Volume |
|---|---|---|---|
| CQ | 1 | 0.14 | 23.2 mg |
| EDMAB | 1 | 0.14 | 27 mg |
| TBAPCCP | 0.5 | 0.07 | 41.8 mg |
| PAG-$SbF_6$ | 1 | 0.14 | 90.4 mg |
| MA | 160 | 22.4 | 2.02 ml |
| TEGDA | 1.6 | 0.224 | 0.06 ml |
| CHO | 20 | 2.8 | 0.26 ml |
| ECC | 20 | 2.8 | 0.62 ml |
| OXAA | 7 | 0.98 | 0.2 ml |

**Photobuffer TBABr**

| Reagents | Equivalents | mmol | Volume |
|---|---|---|---|
| CQ | 1 | 0.14 | 23.2 mg |
| EDMAB | 1 | 0.14 | 27 mg |
| TBABr | 0.5 | 0.07 | 22.6 mg |
| PAG-$SbF_6$ | 1 | 0.14 | 90.4 mg |
| MA | 160 | 22.4 | 2.02 ml |
| TEGDA | 1.6 | 0.224 | 0.06 ml |
| CHO | 20 | 2.8 | 0.26 ml |
| ECC | 20 | 2.8 | 0.62 ml |
| OXAA | 7 | 0.98 | 0.2 ml |

**Photobuffer TBAOAc**

| Reagents | Equivalents | mmol | Volume |
|---|---|---|---|
| CQ | 1 | 0.14 | 23.2 mg |
| EDMAB | 1 | 0.14 | 27 mg |
| TBAOAc | 0.5 | 0.07 | 21.1 mg |
| PAG-$SbF_6$ | 1 | 0.14 | 90.4 mg |
| MA | 160 | 22.4 | 2.02 ml |
| TEGDA | 1.6 | 0.224 | 0.06 ml |
| CHO | 20 | 2.8 | 0.26 ml |
| ECC | 20 | 2.8 | 0.62 ml |
| OXAA | 7 | 0.98 | 0.2 ml |

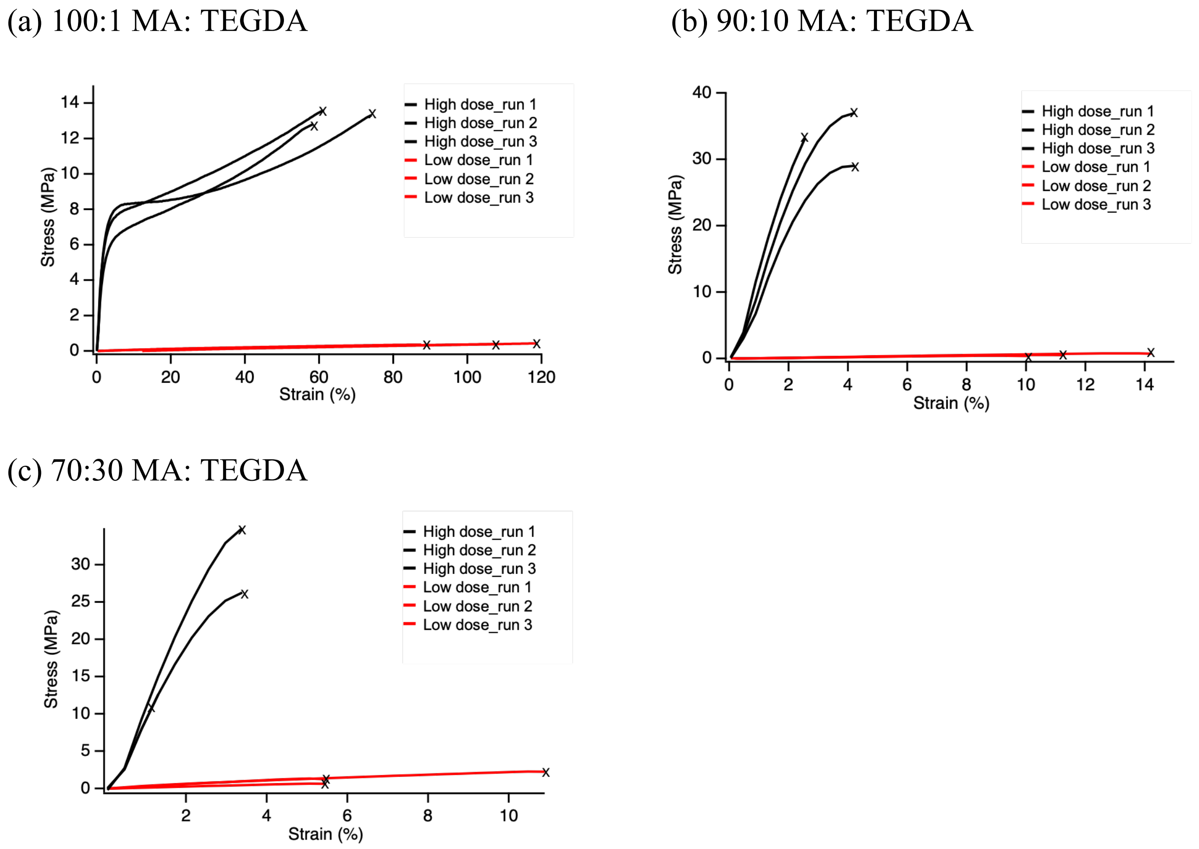


**Figure S19.** Tensile tests for Figure 3C. Low dose films received 30 s irradiation, and high dose films received 60 min irradiation (456 nm, 23 mW/cm$^2$).

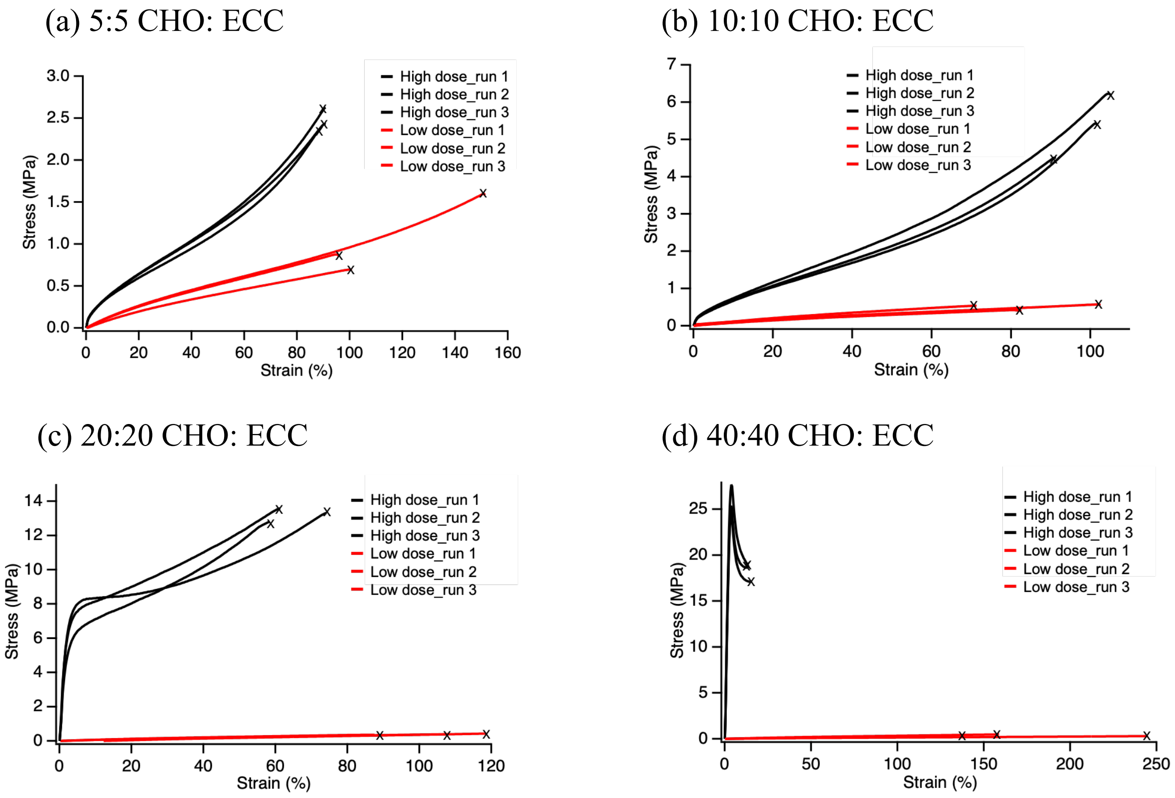


**Figure S20.** Tensile tests for Figure 3D. Low dose films received 30 s irradiation, and high dose films received 60 min irradiation (456 nm, 23 mW/cm$^2$).

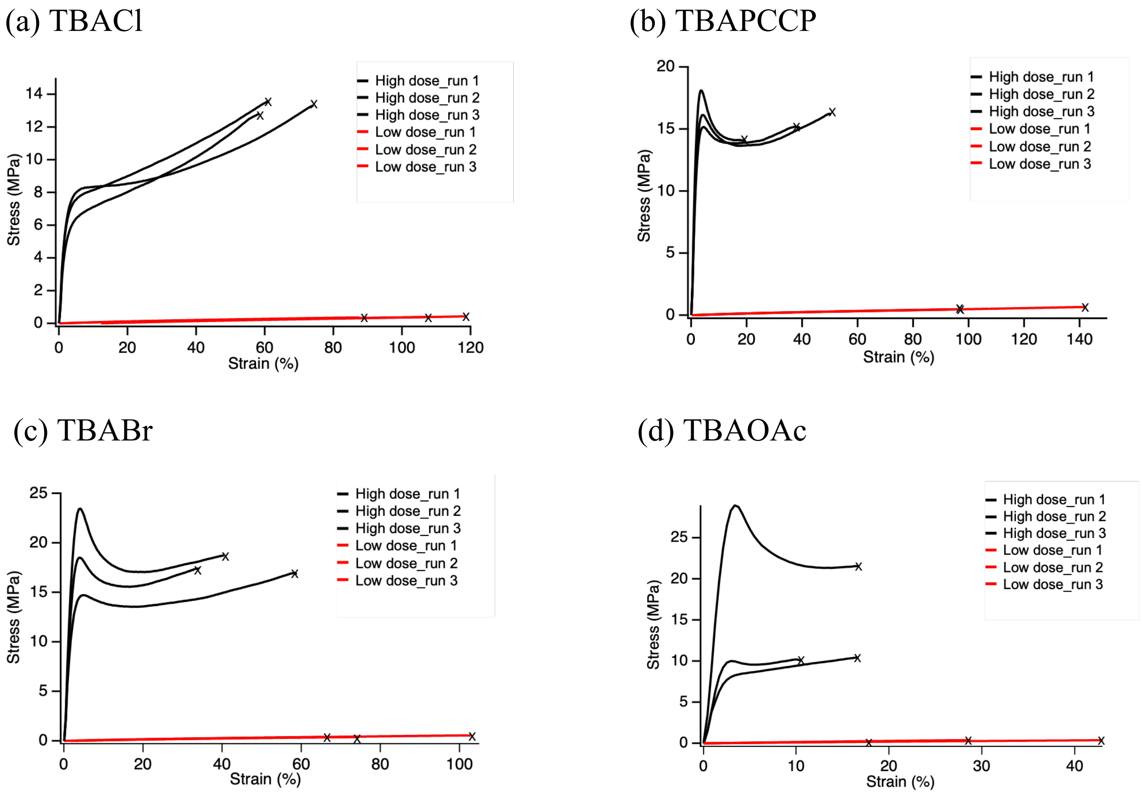


**Figure S21.** Tensile tests for Figure 3C. Low dose films received 30 s irradiation, and high dose films received 60 min irradiation (456 nm, 23 mW/cm$^2$).

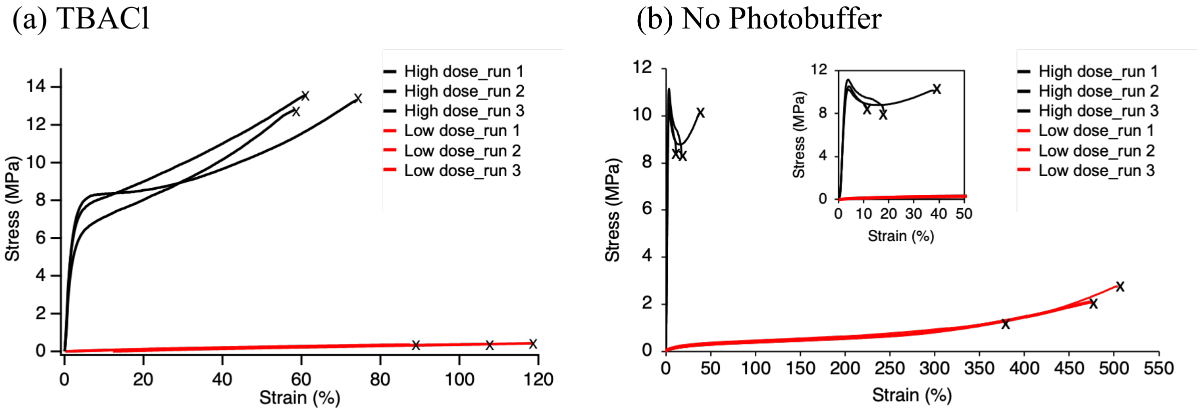


**Figure S22.** Tensile tests for films (same monomers as Fig 3B, 100:1 MA:TEGDA, 20:20 CHO:ECC) made with (A) and without (B) photobuffer. Low dose films received 30 s irradiation, and high dose films received 60 min irradiation (456 nm, 23 mW/cm$^2$). Samples produced with a low light dose and no photobuffer possess a Young's Modulus 4x greater than the analogous film with a photobuffer. Even with just 30 s irradiation, the absence of the photobuffer results in stiffer, stronger films than would otherwise be produced. The high dose film synthesized without photobuffer displays similar tensile properties to the analogous film with photobuffer.

**Table S4.** Young's Modulus Values in Fig 3 B-E, Fig S22.

| Figure | MA:TEGDA | CHO:ECC | Photobuffer | $YM_{low\ dose}$ (MPa) | $YM_{high\ dose}$ (MPa) |
|---|---|---|---|---|---|
| 3B | 100:1 | 20:20 | $Cl^-$ | 0.73 ± 0.07 | 367 ± 37 |
| 3C | 100:1 | 20:20 | $Cl^-$ | 0.73 ± 0.07 | 367 ± 37 |
| | 90:1 | 20:20 | $Cl^-$ | 2.59 ± 1.76 | 1410 ± 295 |
| | 70:30 | 20:20 | $Cl^-$ | 26.63 ± 10.27 | 1340 ± 112 |
| 3D | 100:1 | 5:5 | $Cl^-$ | 1.40 ± 0.20 | 4.61 ± 0.68 |
| | 100:1 | 10:10 | $Cl^-$ | 1.00 ± 0.20 | 6.80 ± 0.50 |
| | 100:1 | 40:40 | $Cl^-$ | 0.42 ± 0.20 | 1121 ± 56 |
| 3E | 100:1 | 20:20 | $PCCP^-$ | 0.85 ± 0.04 | 767 ± 86 |
| | 100:1 | 20:20 | $Br^-$ | 0.91 ± 0.06 | 998 ± 251 |
| | 100:1 | 20:20 | $OAc^-$ | 1.36 ± 0.42 | 732 ± 487 |
| S22 | 100:1 | 20:20 | none | 3.09 ± 1.67 | 564 ± 77 |

### *Gel Fraction Measurements*

Thermosets were synthesized, cut into pieces (~100 mg), massed ($M_o$), and swelled in toluene for 24 h. The supernatant was removed, and the thermosets were dried in a vacuum oven (70 °C) overnight and massed again ($M_f$).

**Equation S2.**

$$Gel\ Fraction = \frac{M_f}{M_o}$$

**Table S5**. Gel fractions of thermosets in Fig 3D.

| MA:TEGDA | CHO:ECC | Photobuffer | $GF_{low\ dose}$ (%) | $GF_{high\ dose}$ (%) |
|---|---|---|---|---|
| 100:1 | 5:5 | $Cl^-$ | 34.0 ± 0.2 | 87.3 ± 1.2 |
| 100:1 | 20:20 | $Cl^-$ | 19.1 ± 1.0 | 73.4 ± 14 |

*GC Experiments and Results*

**Sample preparation (0 s light irradiation):** To quantify the starting material at t = 0, three 0.03 mL aliquots of the reaction mixture (0.04× the volume of the total solution) were diluted into vials containing 1 mL of DCM. Then, 0.6 mL of the diluted solutions were combined with 0.15 mL DCM and 2.3 µL anisole as an external standard for GC-FID analysis.

**Sample preparation (30s, 60 min light irradiation:** In a nitrogen filled glovebox, CQ (5.8 mg, 35 µmol, 1 equiv), EDMAB (6.7 mg, 35 µmol, 1 equiv), TBACl (4.9 mg, 18 µmol, 0.5 equiv), PAG-$SbF_6$ (22.0 mg, 35 µmol, 1 equiv), MA (0.51 mL, 5.6 mmol, 160 equiv), TEGDA (15 µL, 56 µmol, 1.6 equiv), CHO (0.14 mL, 1.4 mmol, 40 equiv), ECC (0.15 mL, 0.70 mmol, 20 equiv), and OXAA (0.05 mL, 0.24 mmol, 7 equiv) were combined in a 2.5 cm x 2.5 cm silicone mold. The solution was mixed until homogenous and capped with a Petri dish before removal from the glovebox. The reaction mixture was irradiated with a blue Kessil lamp while cooling with compressed air. At two different timepoints (30 s, 60 m), the reaction mixture was removed from the light source, opened to air, and quenched with 0.25 mL 5% TEA in $^{i}$PrOH. Three 0.5 cm x 0.5 cm squares (0.04x the area of the total film) were cut from each film and swelled in 1 mL DCM. After swelling for 16h, 0.6 mL of each supernatant was combined with 0.15 mL DCM and 2.3 µL anisole as an internal standard for GC-FID analysis.

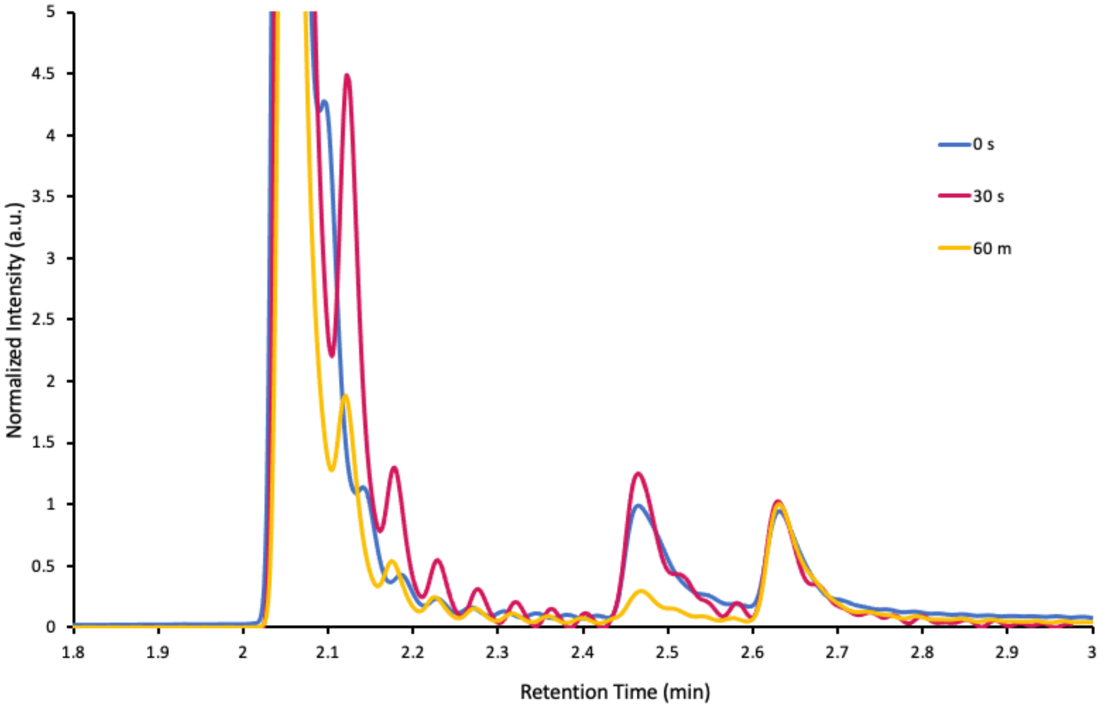


**Figure S23**. Representative GC-FID chromatograms of supernatants from films that were irradiated for 0 s (blue), 30 s (magenta), and 60 m (yellow). CHO elution time is 2.47 min, and anisole elution time is 2.64 min.

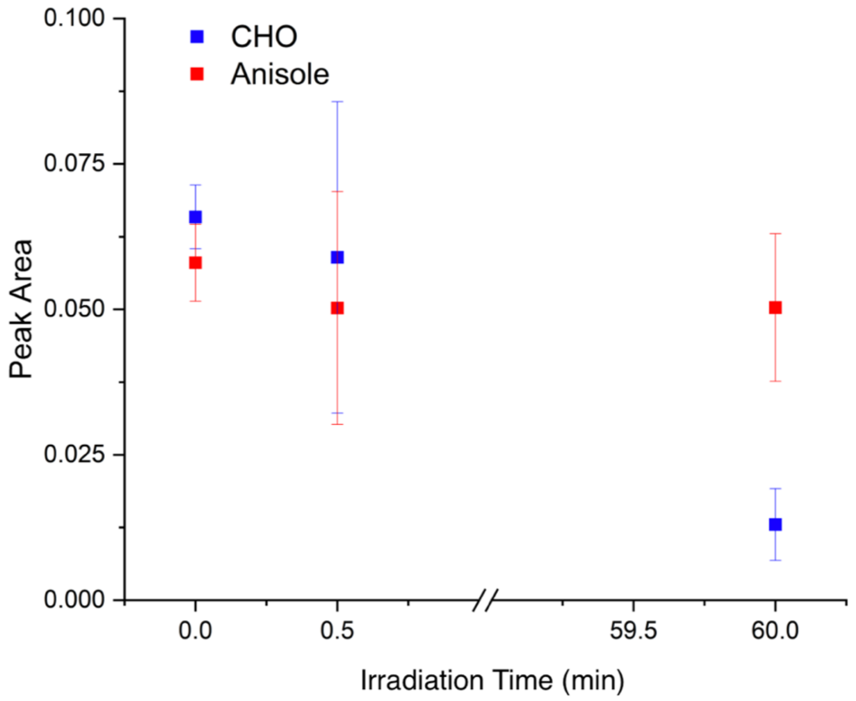


**Figure S24.** Average peak areas of CHO (blue) and anisole (red) vs. irradiation time

***Procedure for Cyclic Tensile Testing of Spatially Patterned Films***

In a nitrogen filled glovebox, an oven-dried reaction vessel was charged with CQ (25.7 mg, 0.16 mmol, 1 equiv), EDMAB (30 mg, 0.16 mmol, 1 equiv), TBACl (21.6 mg, 0.08 mmol, 0.5 equiv), PAG-$SbF_6$ (100.4 mg, 0.16 mmol, 1 equiv), MA (2.24 ml, 25.6 mmol, 160 equiv), TEGDA (0.067 ml, 0.256 mmol, 1.6 equiv), CHO (0.3 ml, 3.2 mmol, 20 equiv), ECC (0.68 ml, 3.2 mmol, 20 equiv), and OXAA (0.22 ml, 1.12 mmol, 7 equiv). The vessel was sealed with a clamp and removed from glovebox, then irradiated with a blue Kessil lamp (456 nm, 25% light intensity, 23 $mW/cm^2$) while cooling by blowing compressed air. After 10 s, the vessel was irradiated with a blue Thorlabs collimated LED (455 nm, 500 mW, 86 $mW/cm^2$) equipped with two convex lenses and a photomask to pattern the polymer film. After 1 hour of irradiation, the vessel was opened to air. The crosslinked film was dried overnight without prior swelling and cut into a dogbone shape before cyclic tensile testing.

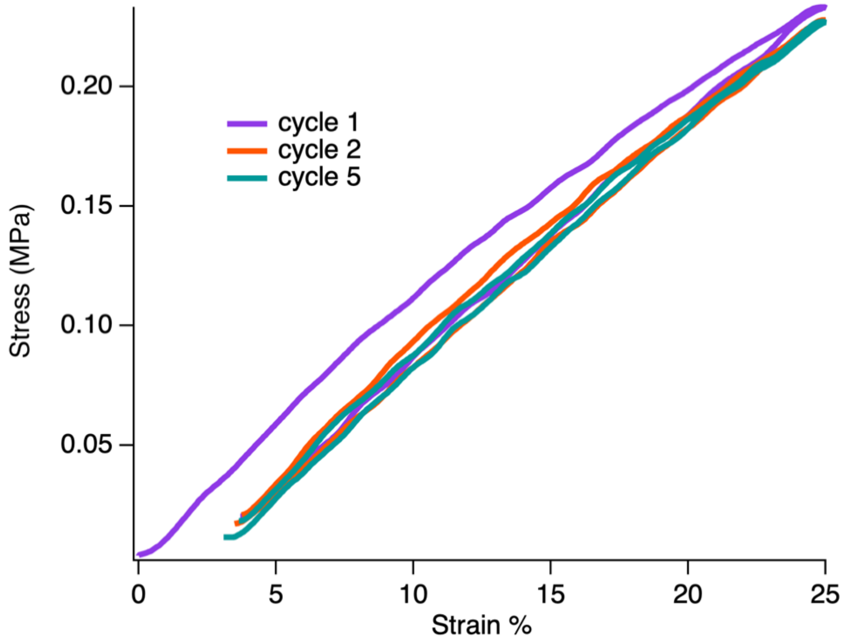


**Figure S25.** Stress vs. strain graph for cyclic testing at 25 mm/min up to 25% strain for 5 cycles

***DMA Experiments and Results***

In a nitrogen filled glovebox, an oven-dried reaction vessel was charged with CQ (25.7 mg, 0.16 mmol, 1 equiv), EDMAB (30 mg, 0.16 mmol, 1 equiv), TBACl (21.6 mg, 0.08 mmol, 0.5 equiv), PAG-$SbF_6$ (100.4 mg, 0.16 mmol, 1 equiv), MA (2.24 ml, 25.6 mmol, 160 equiv), TEGDA (0.067 ml, 0.256 mmol, 1.6 equiv), CHO (0.3 ml, 3.2 mmol, 20 equiv), ECC (0.68 ml, 3.2 mmol, 20 equiv), and OXAA (0.22 ml, 1.12 mmol, 7 equiv). The vessel was sealed with a clamp and removed from the glovebox, then irradiated with a blue Kessil lamp (456 nm, 25% light intensity, 23 mW/cm$^2$) while cooling by blowing compressed air. After 10 s, the vessel was further irradiated with a blue Thorlabs collimated LED (455 nm, 500 mW, 86 mW/cm2) equipped with two convex lenses and a photomask to expose only the middle region of the crosslinked film with light. After 1 hour irradiation, the vessel was opened to air and the crosslinked film was cut into 6.4mm x 2.7mm x 1.5mm (hard) and 6.4mm x 3.7mm x 1.5mm (soft) strips for DMA.

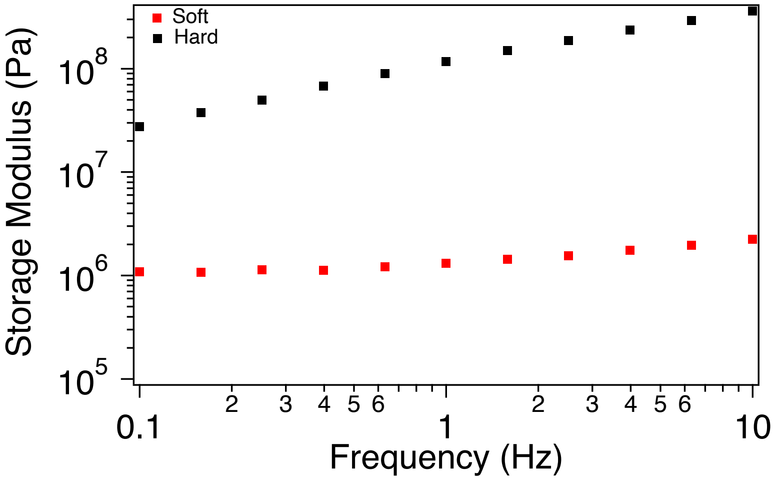


**Figure S26.** DMA results for spatial control over crosslinked acrylate-epoxide film in Fig. 4B.

*FTIR and Printer LED Spectra*

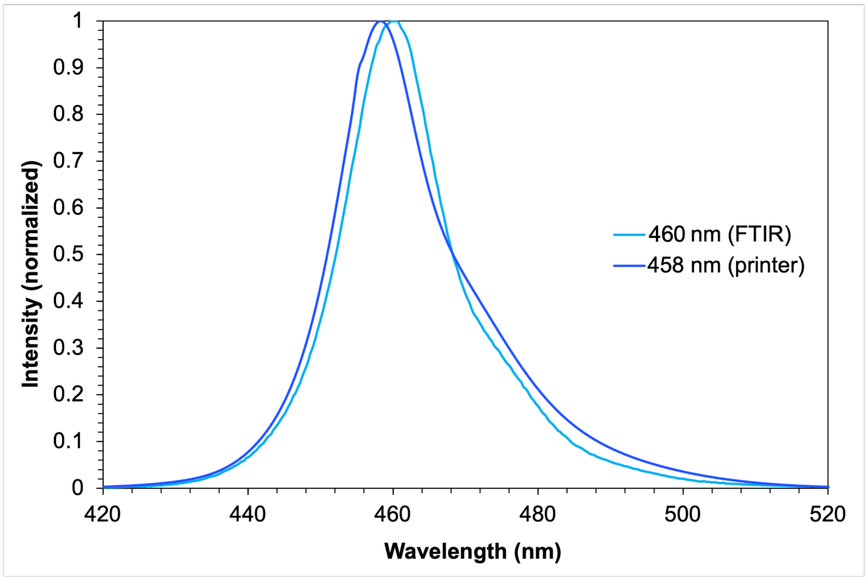


**Figure S27.** Spectral output of the blue LEDs used for FTIR (460 nm peak, 16 nm FWHM) and 3D printing (458 nm peak, 17 nm FWHM)

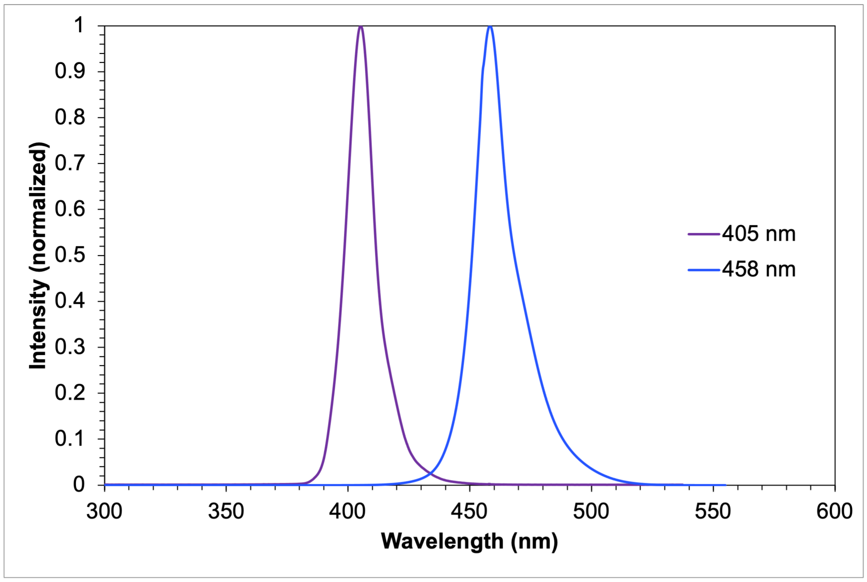


**Figure S28.** Spectral output of the LEDs used for 3D printing with blue light (458 nm, 17 nm FWHM) and violet light (405 nm, 13 nm FWHM).

***Resin Formulation Optimization***

Resin batches were prepared by varying concentration of the photosystem (CQ, EDMAB, PAG-$SbF_6$), buffering salt (TBACl), and radical scavenger (TEMPO), and assessed qualitatively after printing. Color, resolution, and stiffness were probed and noted before the next iteration of resin formulations. Initial prints using the optimized resin formulation described above led to insufficient curing of the epoxide domains and lateral overcuring of the acrylate domains. Notably, all printing was accomplished under an ambient atmosphere. The optimized 3D printing system ultimately resulted from a 2× increase in photosystem concentration, a 0.5× decrease in buffering salt, and the addition of 0.25 wt% TEMPO to improve lateral resolution. A range of intensities and exposure times were then tested, ultimately selecting 40 seconds per 50 μm layer and 25 mW/cm$^2$ (soft system) and 500 mW/cm$^2$ (hard system) light intensities. RT-FTIR analysis of this optimized resin revealed that cationic polymerization lag times decreased under all light intensities, illustrating that photobuffer concentration controls the light dosage necessary to distinguish radical vs. cationic polymerizations (Fig S30).

**Table S6.** Blue light resin formulation for 460 nm DLP 3D printing

| Reagents | Equivalents | mol % | wt% |
|---|---|---|---|
| CQ | 2 | 0.93 | 1.35 |
| EDMAB | 2 | 0.93 | 1.57 |
| TBACl | 0.25 | 0.12 | 0.28 |
| PAG-$SbF_6$ | 2 | 0.93 | 5.24 |
| MA | 160 | 74.33 | 55.91 |
| TEGDA | 1.6 | 0.74 | 1.96 |
| CHO | 20 | 9.29 | 7.97 |
| ECC | 20 | 9.29 | 20.48 |
| OXAA | 7 | 3.25 | 5.23 |
| TEMPO | 0.4 | 0.19 | 0.25 |

The following violet light resin was formulated for printing with a 405 nm light source, using ITX as a photoinitiator, rather than the EDMAB/CQ blue light photoinitiating pair.

**Table S7.** Violet light resin formulation for 405 nm DLP 3D printing

| Reagents | Equivalents | mol % | wt% |
|---|---|---|---|
| ITX | 1 | 0.47 | 1.08 |
| TBACl | 0.5 | 0.24 | 0.59 |
| PAG-$SbF_6$ | 1 | 0.47 | 2.73 |
| MA | 160 | 75.79 | 58.38 |
| TEGDA | 1.6 | 0.76 | 2.05 |
| CHO | 20 | 0.17 | 8.31 |
| ECC | 20 | 0.43 | 21.39 |
| OXAA | 7 | 3.32 | 5.46 |

*Kin*etic Analysis via RT-FTIR Spectroscopy

Each sample was placed in between two salt plates or two glass slides that were separated by spacers with 50 µm or 100 µm thicknesses before irradiation with a 460 nm LED (original or optimized blue light resin) (Type H, Mightex) or 405 nm LED (violet light resin) (Type B, Mightex). OPUS spectroscopy software was used to obtain spectra, monitoring peak area at 3100 $cm^{-1}$ (C=C stretch) to assess acrylate conversion over time.[7-9] To monitor epoxide conversion, resin was prepared with the same ratio, but excluding acrylate groups (MA, TEGDA, OXAA) to avoid a peak deconvolution, and either 909 (C–O–C asymmetric stretch) or 3915 $cm^{-1}$ (overtone of C–O–C asymmetric stretch) were used to determine epoxy conversion over time.[10-14] All trials were completed in duplicate. The original resin formulation mentioned in this section refers to 1-equivalent of each photosystem component, 0.5-equivalents of TBACl, and no TEMPO.

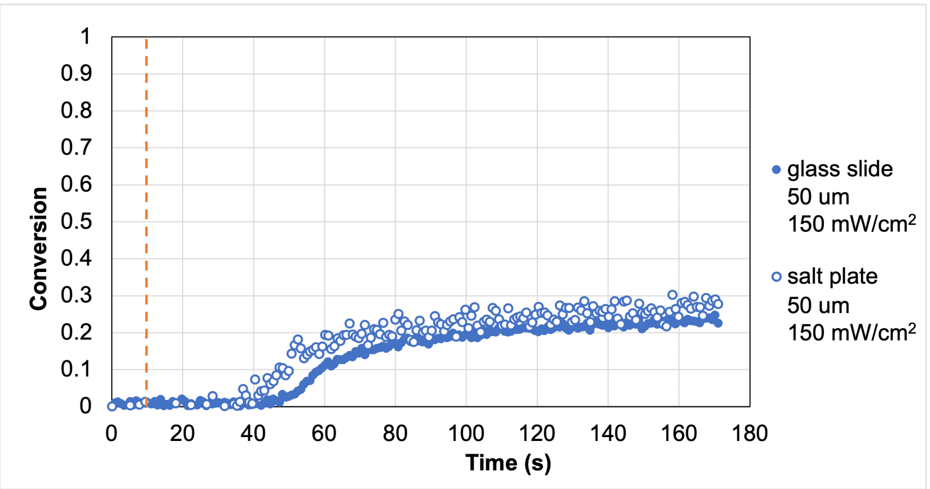


**Figure S29.** Epoxide conversion over time for the original blue light resin system with 50 µm spacers, 150 $mW/cm^2$ light, and different substrates to hold the resin (glass slides or salt plates). The dashed line indicates the time at which the 460 nm LED was turned on. The peak area at 3915 $cm^{-1}$ was tracked for glass slides, and the peak area at 909 $cm^{-1}$ was tracked for salt plates.

In typical FTIR analysis, epoxides are commonly identified by their asymmetric C-O-C stretch, which appears at 909 $cm^{-1}$ for CHO. However, RT-FTIR requires sandwiching the reactive resin between substrate panels (typically glass), and glass strongly absorbs in the fingerprint region of the IR spectrum, which obscures the evolution of the 909 $cm^{-1}$ stretch over time. To circumvent this, we used salt plates as a substrate, which do not absorb in the lower wavelength IR regions and were able to track the 909 $cm^{-1}$ peak. However, upon high dose curing the salt plates could not be recovered, and owing to their higher cost we switched to using glass slides for further experiments. CHO also displays a vibrational overtone at 3915 $cm^{-1}$, and we hypothesized that since glass does not absorb wavelengths in this region, we could track this peak for FTIR analysis. Indeed, the epoxide conversions obtained by tracking the 3915 $cm^{-1}$ peak with glass slides and the 909 $cm^{-1}$ peak in salt plates were similar, so glass slides were used as substrates moving forward.

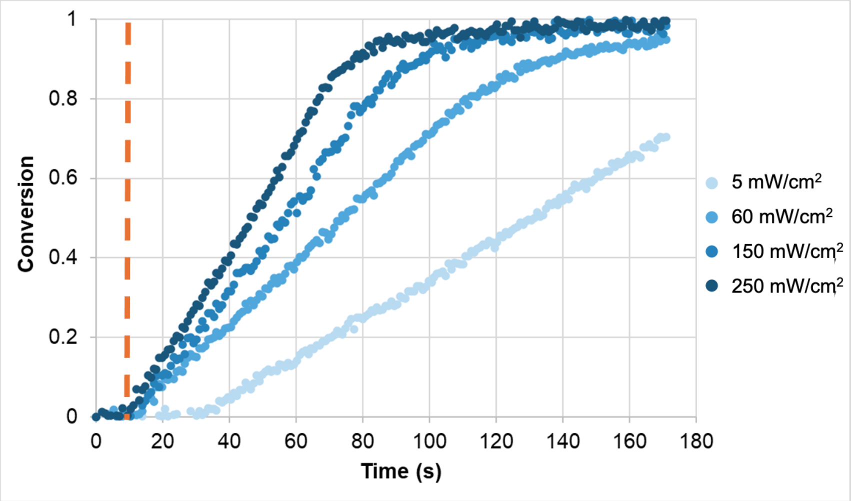


**Figure S30.** Acrylate conversion over time for the original blue light resin system with 50 µm spacers and different light intensities. The peak area at 3100 $cm^{-1}$ was tracked here. The dashed line indicates the time at which the 460 nm LED was turned on.

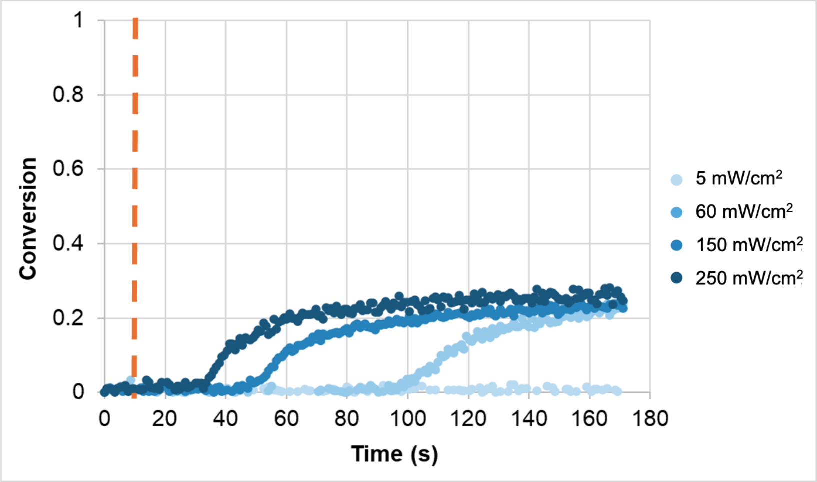


**Figure S31.** Epoxide conversion over time for the original blue light resin system with 50 µm spacers and different light intensities. The peak area at 3915 $cm^{-1}$ was tracked here. The dashed line indicates the time at which the 460 nm LED was turned on.

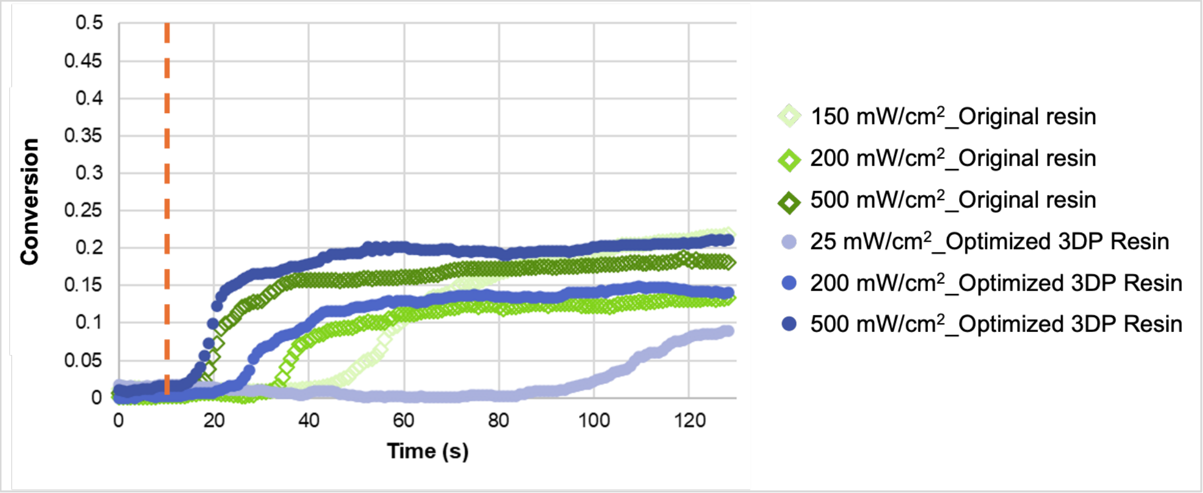


**Figure S32.** Epoxide conversion over time for the original blue light resin system (green shades) relative to the system optimized for 3D printing (blue shades), both with 50 µm spacers and different light intensities. The peak area at 3915 $cm^{-1}$ was tracked here. To calculate epoxide conversion, the FTIR traces were first baseline corrected and smoothed in MATLAB using the *smoothdata* function with a moving median approach and a *window* set to 20. The dashed line indicates the time at which the 460 nm LED was turned on.

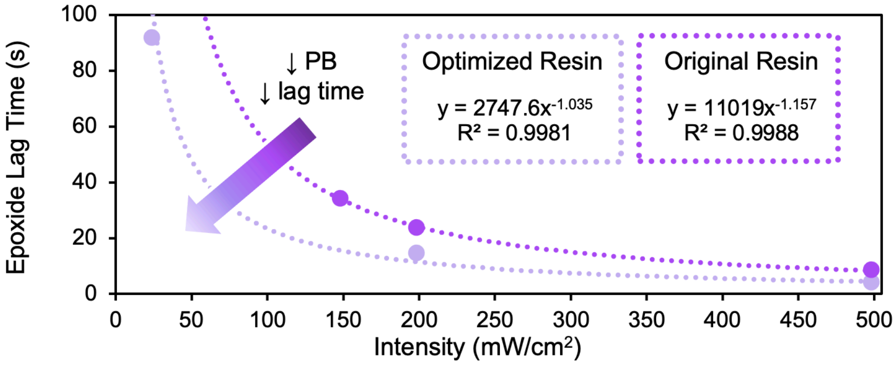


**Figure S33.** Lag time calculated as the time between the 460 nm light turning on and the epoxide conversion starting to increase compared between the original and 3D printing optimized blue light resin formulations. PB = photobuffer.

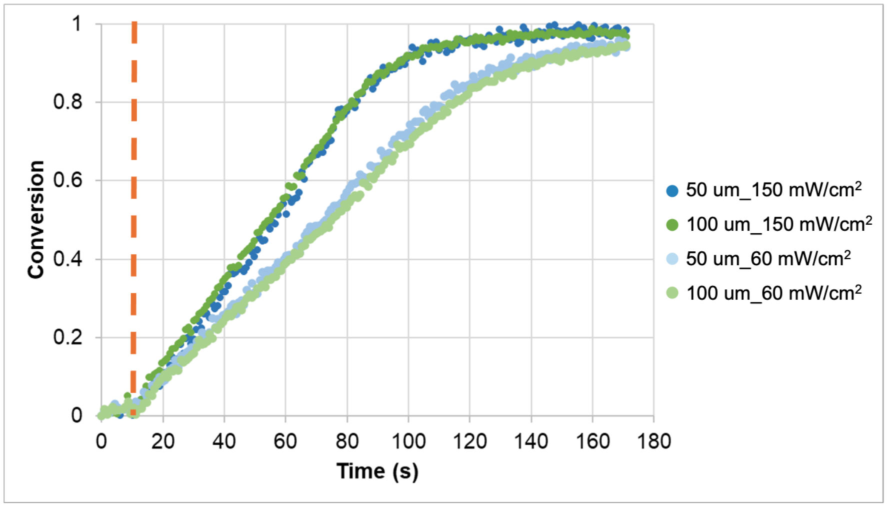


**Figure S34.** Acrylate conversion over time for the original blue light resin system with 50 or 100 μm spacers and different light intensities. The peak area at 3100 cm$^{-1}$ was tracked here. The dashed line indicates the time at which the 460 nm LED was turned on.

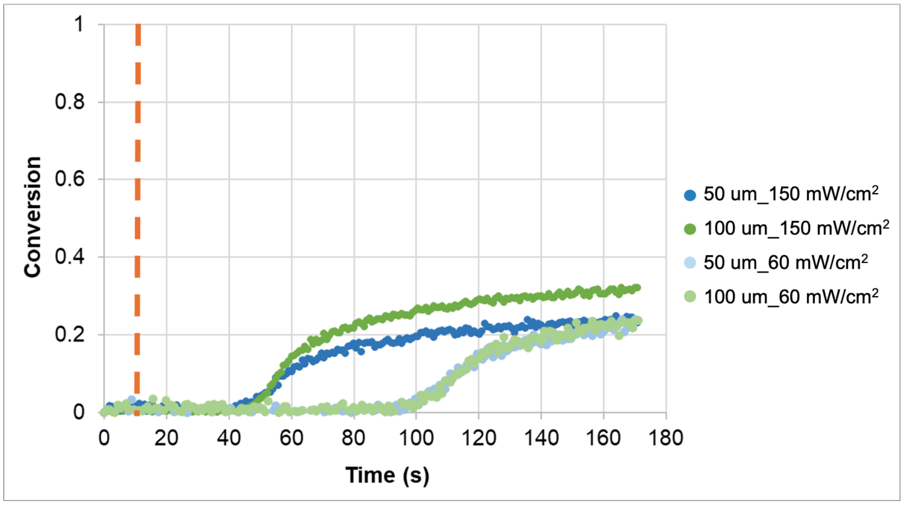


**Figure S35.** Epoxide conversion over time for the original blue light resin system with 50 or 100 μm spacers and different light intensities. The peak area at 3915 cm$^{-1}$ was tracked here. The dashed line indicates the time at which the 460 nm LED was turned on.

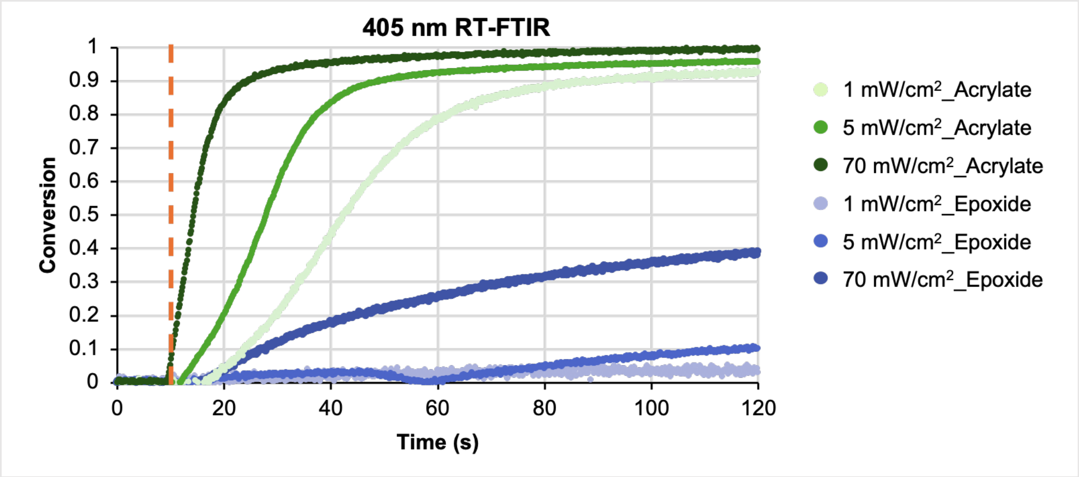


**Figure S36.** Conversion of acrylate (green traces) and epoxide (blue traces) over time calculated by tracking 3100 $cm^{-1}$ in the violet light resin system (containing ITX in place of EDMAB/CQ) and 3915 $cm^{-1}$ in the epoxy only violet light resin system at 1, 5, and 70 $mW/cm^2$ intensities. Dashed line indicates the time at which the 405 nm LED was turned on.

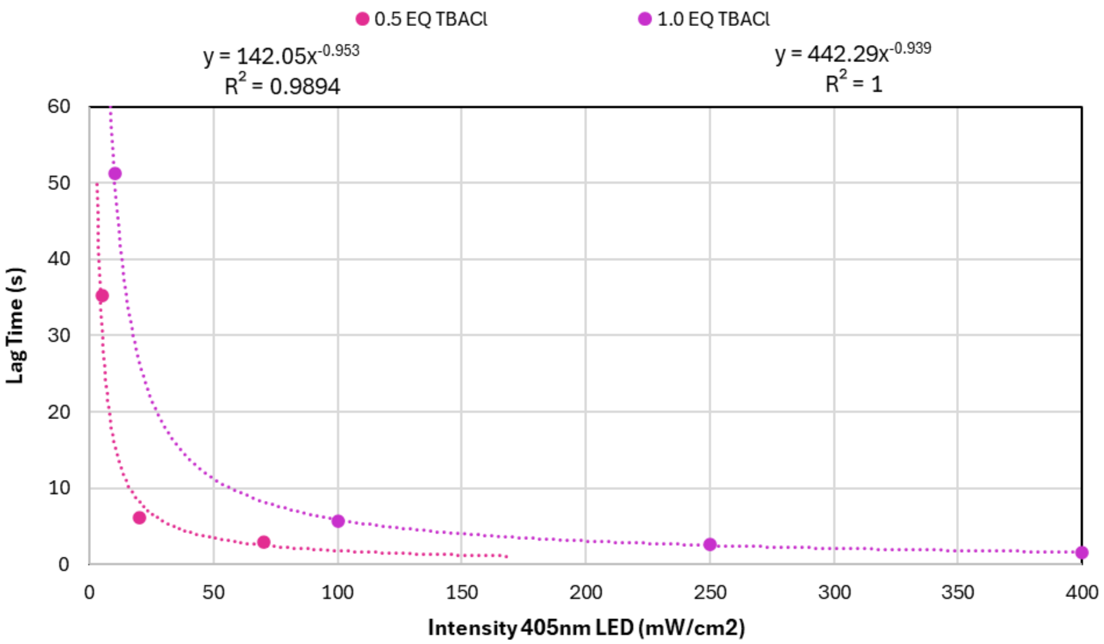


**Figure S37.** Lag time calculated as the time between the 405 nm light turning on and the epoxide conversion starting to increase compared between the violet light resin and that same resin with double the TBACl photobuffer loading (1.0 equiv).

### *Grayscale File Generation*

A range of 8-bit grayscale values (0-255) were displayed on the 3D printer and the corresponding intensity values were measured with a ThorLabs PM100D photometer equipped with a thermal power sensor (S401C). This data was used to select grayscale values that would provide the desired intensity levels. Grayscale values of 12 and 250 were used to provide an intensity of 25 and 500 mW/cm$^2$, respectively for the 460 nm LED, while grayscale values of 4 and 249 were used to provide an intensity of 1 and 75 mW/cm$^2$ respectively for the 405 nm LED.

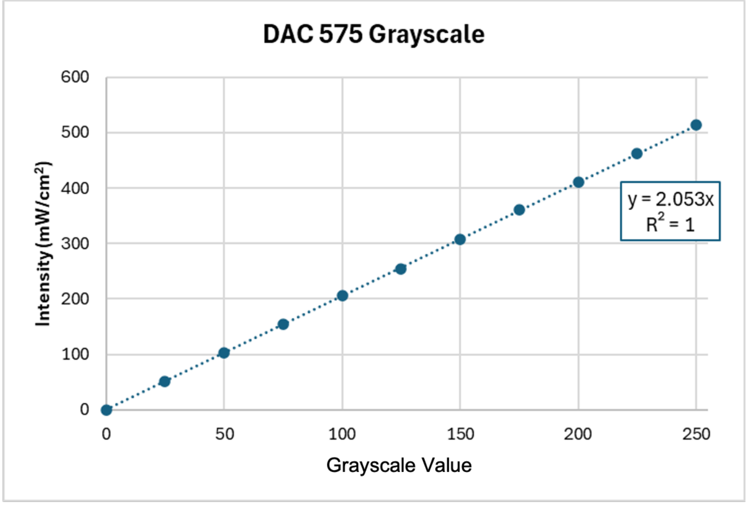


**Figure S38.** Graph of grayscale value versus intensity level of the 460 nm LED equipped within the MONO3-VZ1 3D DLP printer.

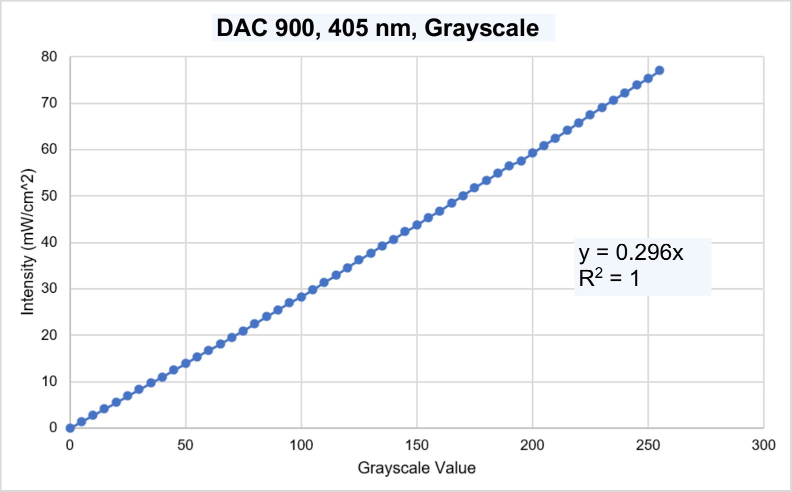


**Figure S39.** Graph of grayscale value versus intensity level of the 405 nm LED equipped within the MONO3-VZ1 3D DLP printer.

***Complex Grayscale Projections for 3D Printing***

*MATLAB Image Processing.* Custom MATLAB scripts were developed to integrate with data derived from 3D models generated via CAD software. These scripts are adept at executing advanced image processing operations, enabling the application of personalized grayscale patterns that correspond directly to gradient intensity levels. Our methodology for achieving grayscale manipulation hinges upon the utilization of our custom Digital Light Processing (DLP) printers and modulation of micromirror and LED activation intervals as detailed in the figure below.

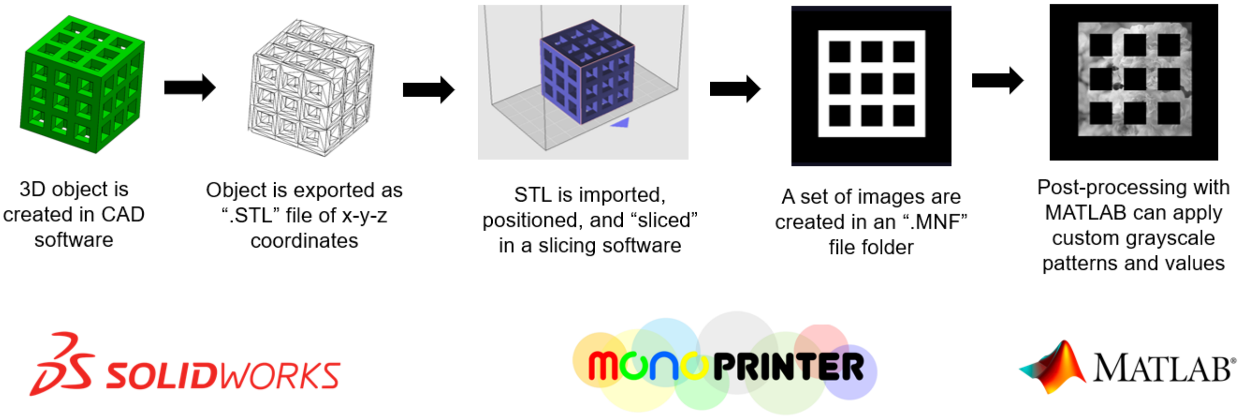


**Figure S40.** Overview of grayscale processing for 3D printing and software programs involved.

*Multi-Material Bar Printing*

The following print file was created to showcase the ability to produce hard and soft segments within a single 3D print. A simple rectangular design was first constructed and nanoindentation was performed to assess mechanical differences. Each square was 4 mm × 4 mm and the entire print was 2 mm in height (50 µm layers). Prints were immediately washed with IPA after printing and then taken to the Nanoindenter. Indents were performed on the soft segment within 2 hours after printing and the hard segment was allowed overnight air drying before indenting to match post-processing conditions used for tensile testing.

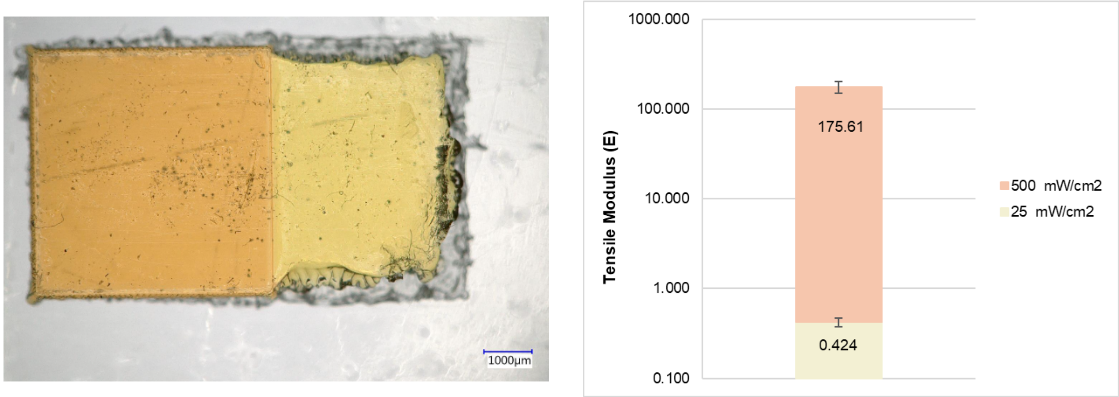


**Figure S41.** Image (left) of a 460 nm multimaterial print for nanoindentation where a 4 mm × 4 mm square was cured with 500 mW/cm$^2$ (darker orange, left) connected to another 4 mm × 4 mm square cured with 25 mW/cm$^2$ (lighter yellow, right). An exposure time of 40 s/50 µm layer was used for each region. Corresponding nanoindentation data (right) showing the calculated tensile modulus for indents performed in triplicate on each side of the print. Error bars represent plus or minus one standard deviation from three indentations.

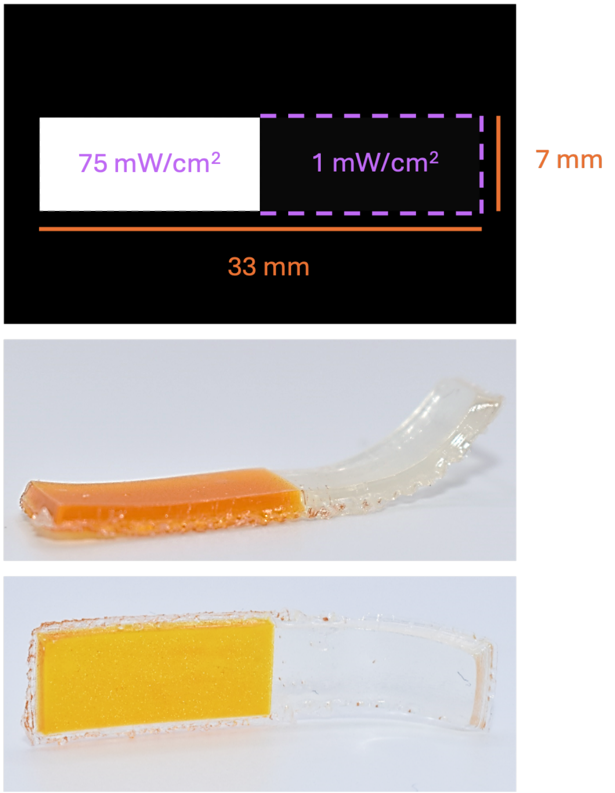


**Figure S42.** Multi-Material printing using the violet light resin and a 405 nm LED with an exposure time of 50 s/50 µm layer. Grayscale value scheme (top) of multi-material print where a 16.5 mm × 7 mm rectangle was cured with 75 mW/cm$^2$ connected to another 16.5 mm × 7 mm rectangle cured with 1 mW/cm$^2$ light. Side view (middle) and front view (bottom) of the corresponding print.

***Resolution Prints***

The following print file was created to determine the minimum lateral resolution achievable using the optimized hard and soft resins. The prints consisted of 10 larger rectangular base layers and 3 layers for the small square features. One pixel has dimensions of 3.78 × 3.78 µm. The Soft system was printed with high intensity on the first 10 layers (larger rectangular base) to achieve good adhesion to the build plate, while the remaining layers were printed with lower intensity to examine soft system resolution. Exposure time was 40 s/50 µm layer

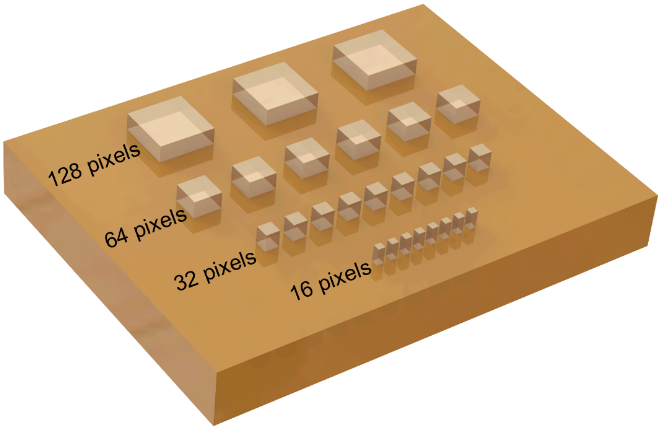


**Figure S43.** STL file rendering created in-house that contains an array of various sized features from 16 × 16 pixel cubes to 128 × 128 pixel cubes, where one pixel has dimensions of 3.78 × 3.78 µm.

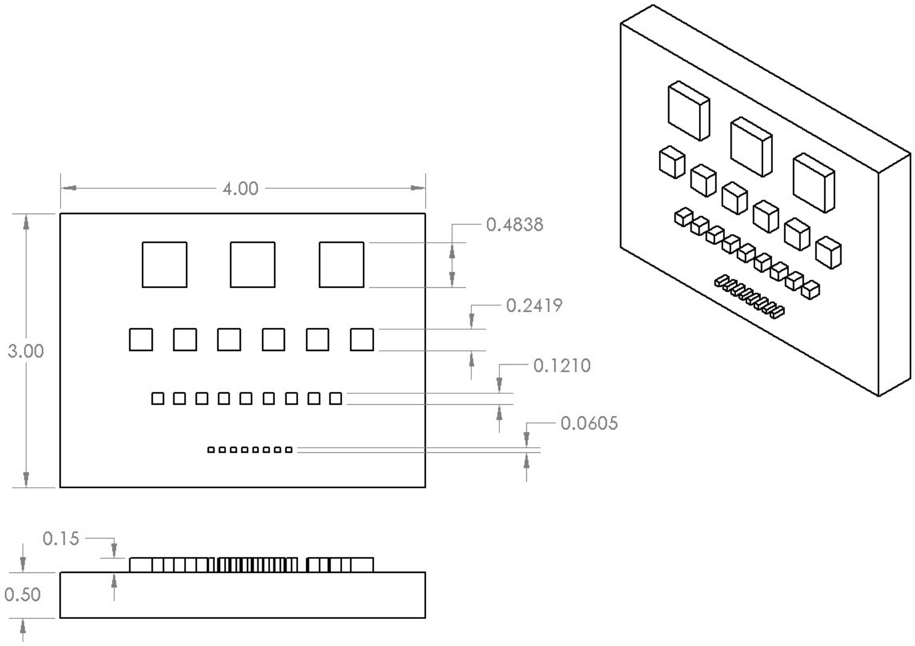


**Figure S44.** Engineering drawing created in-house of the resolution print file; all units are in millimeters.

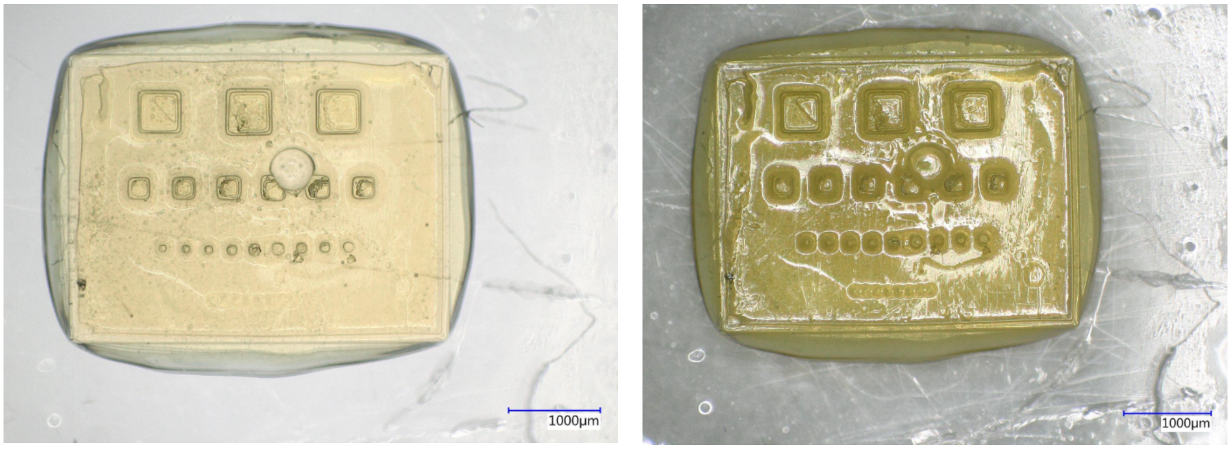


**Figure S45.** Bottom lit (left) and top lit (right) digital microscope images of the resolution print completed at 25 mW/cm$^2$ 460 nm light (soft system).

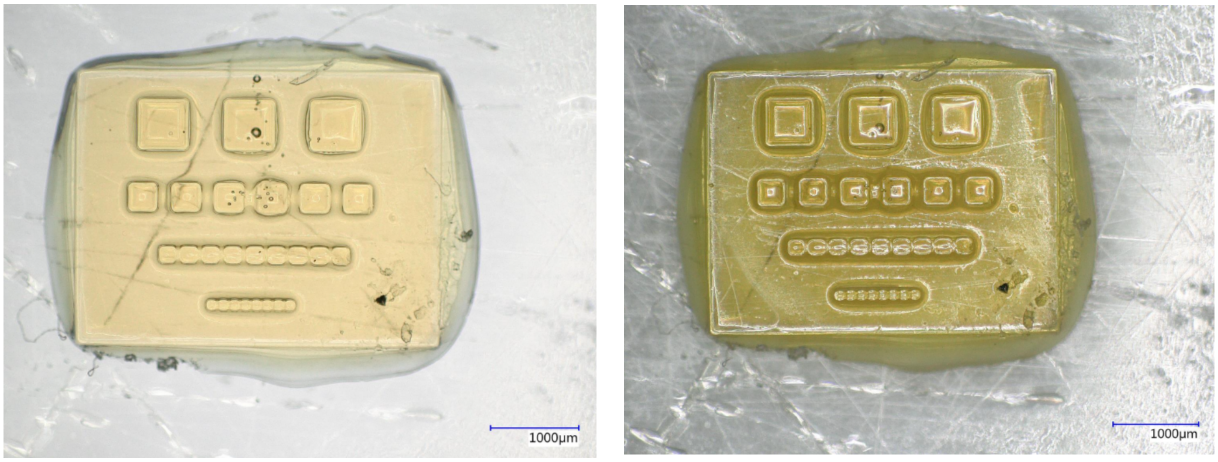


**Figure S46.** Bottom lit (left) and top lit (right) digital microscope images of the resolution print completed at 500 mW/cm$^2$ 460 nm light (hard system).

***Multi-Material Hybrid Longhorn-Bear Print***

The following print file was created to showcase the resolution and complexity achieved through optimization of this resin system and print settings. All STL files to create this print were downloaded through a free STL website.[15-18]

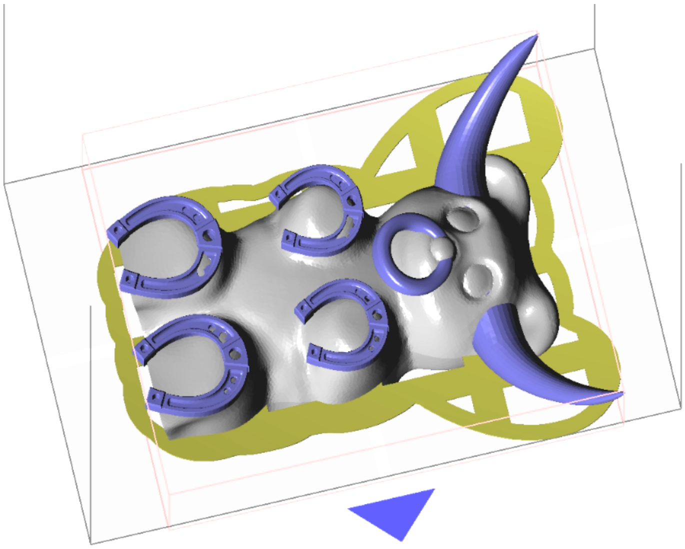

**Figure S47.** STL rendering of the longhorn-bear print as aligned for printing in MonoWare software. The yellow layer was added as a global base to improve adhesion to the build plate. The purple objects are set to print with high intensity and the gray object is set to print with low intensity. This print was completed using 460 nm light exposure for 40 seconds per 50 μm layer.

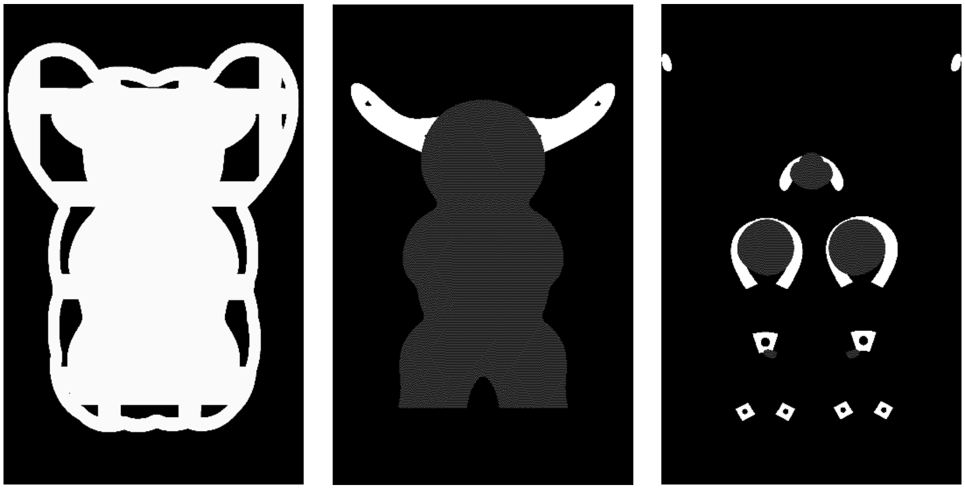

**Figure S48.** Examples of grayscale projection images where the gray areas correspond to 25 $mW/cm^2$ and the white areas correspond to 500 $mW/cm^2$ 460 nm light. The first image (left) shows the global base layer, which was cured at a high intensity to assist with adhesion of the print to the build plate.

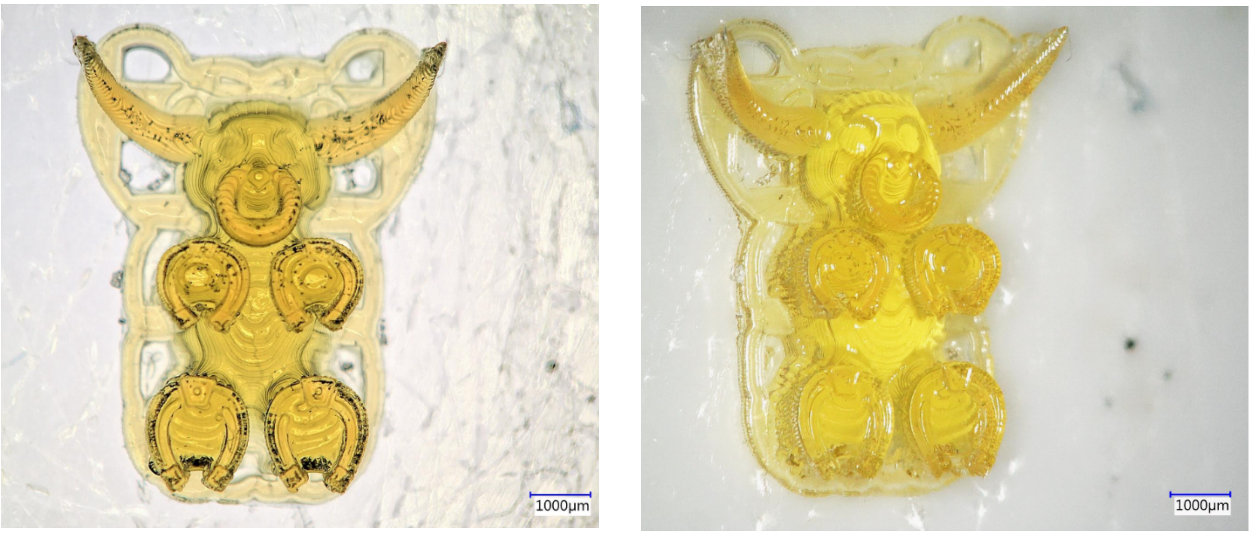


**Figure S49.** Images of the longhorn-bear print taken using a digital microscope with high definition engaged (left) and a 20-degree tilt with a depth of focus correction (right).

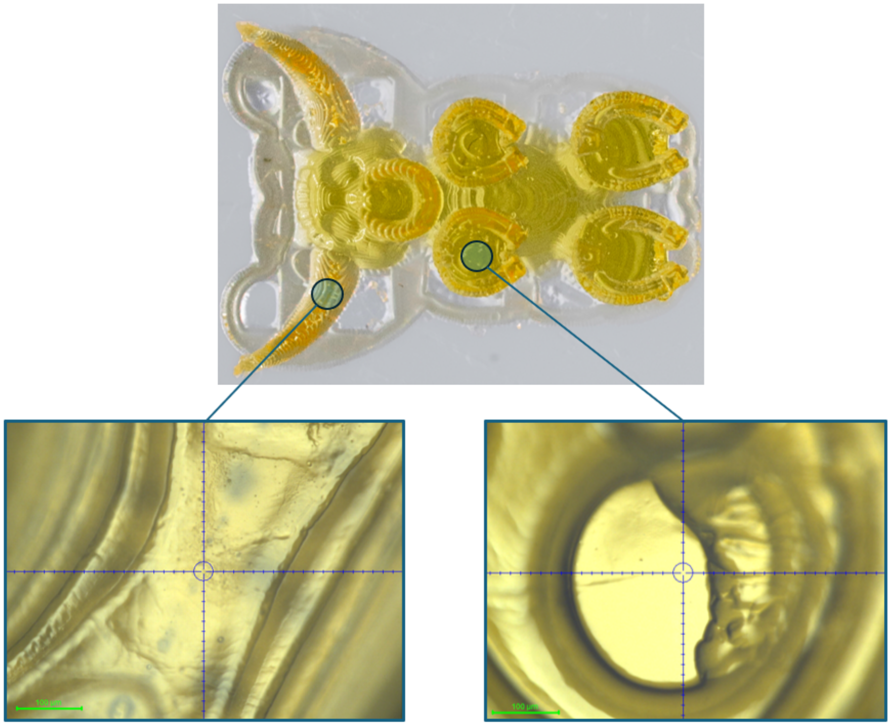

**Figure S50.** Representative image of the longhorn-bear print and corresponding nanoindentation locations for hard (left) and soft (right) systems as viewed using the Hysitron instrument camera and Triboscan software.

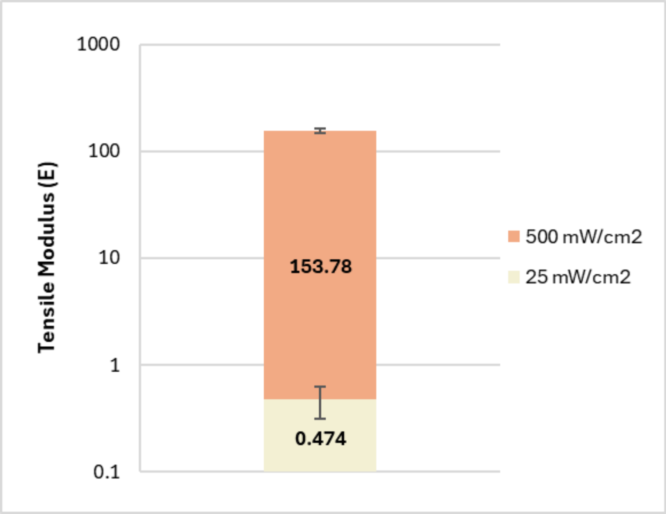


**Figure S51.** Nanoindentation data showing the calculated tensile modulus for indents performed in triplicate on both the hard and soft selected locations on the longhorn-bear print. Error bars represent plus or minus one standard deviation from three indentations.

***References.***


1. Pal, J.; Kankariya, N.; Sanwaria, S.; Nandan, B.; Srivastava, R. K., Control on molecular weight reduction of poly(epsilon-caprolactone) during melt spinning--a way to produce high strength biodegradable fibers. *Mater Sci Eng C Mater Biol Appl* **2013,** *33* (7), 4213-20.
2. Dolinski, N. D.; Page, Z. A.; Callaway, E. B.; Eisenreich, F.; Garcia, R. V.; Chavez, R.; Bothman, D. P.; Hecht, S.; Zok, F. W.; Hawker, C. J., Solution Mask Liquid Lithography (SMaLL) for One-Step, Multimaterial 3D Printing. *Adv. Mater.* **2018,** *30* (31), 1800364.
3. Kottisch, V.; Jermaks, J.; Mak, J. Y.; Woltornist, R. A.; Lambert, T. H.; Fors, B. P., Hydrogen Bond Donor Catalyzed Cationic Polymerization of Vinyl Ethers. *Angew. Chem. Int. Ed. Engl.* **2021,** *60* (9), 4535-4539.
4. Radtke, M. A.; Dudley, C. C.; O'Leary, J. M.; Lambert, T. H., A Scalable, One-Pot Synthesis of 1,2,3,4,5-Pentacarbomethoxycyclopentadiene. *Synthesis (Stuttg)* **2019,** *51* (5), 1135-1138.
5. Bou, S. J. M. C.; Ellis, A. V. Microfluidic Devices using Thiol-Ene Polymers. In *SPIE Micro+Nano Materials, Devices, and Applications, Proceedings Vol. 8923,* Melbourne, Australia; Friend, J.; Hoe, T. H, Eds.; SPIE, 2014; 327-339.
6. Borda, E.; Medagoda, D. I.; Airaghi Leccardi, M. J. I.; Zollinger, E. G.; Ghezzi, D. *Biomaterials* **2023**, *293*, 121979. Conformable Neural Interface Based on Off-Stoichiometry Thiol-Ene-Epoxy Thermosets. *Biomaterials* **2023**, *293*, 121979.
7. Arjunan, V.; Subramanian, S.; Mohan, S. FTIR and FTR Spectral Studies of 2-Amino-6-Bromo-3-Formylchromone. *Spectrochim. Acta A Mol. Biomol. Spectrosc.* **2004**, *60*, 995–1000.
8. Gunasekaran, S.; Sailatha, E.; Seshadri, S.; Kumaresan, S. FTIR, FT Raman Spectra and Molecular Structural Confirmation of Isoniazid. *Indian J. Pure Appl. Phys*. **2009**, *47*, 12–18.
9. Arivazhagan, G.; Elangovan, A.; Shanmugam, R.; Vijayalakshmi, R.; Karthick, N. K. Study of Molecular Interaction in the Mixtures of Benzene + Methyl Acrylate/Butyl Acrylate through Dielectric and Spectroscopic Studies. *J. Mol. Liq*. **2016**, *214*, 357–363.
10. Wang, X.; Nabipour, H.; Kan, Y.-C.; Song, L.; Hu, Y. A Fully Bio-Based, Anti-Flammable and Nontoxic Epoxy Thermosetting Network for Flame-Retardant Coating Applications. *Prog. Org. Coat.* **2022**, *172*, 107095.
11. Zhang, W. et al. Synthesis and Characterization of Gel Polymer Electrolyte Based on Epoxy Group via Cationic Ring-Open Polymerization for Lithium-Ion Battery. *Membranes* **2022**, *12*, 439.
12. Nishikubo, T.; Kameyama, A.; Toya, Y. Synthesis of Photoreactive Imidazole Derivatives and Thermal Curing Reaction of Epoxy Resins Catalyzed by Photo-Generated Imidazole. *Polym. J.* **1997**, *29*, 450–456.
13. Nishikubo, T.; Kameyama, A.; Kashiwagi, K.; Oyama, N. Novel Thermal Curing Reactions of Epoxy Resin and Poly(Glycidyl Methacrylate) Using Photo-generated Difunctional Thiols. *Polym. J.* **1996**, *28*, 795–800.
14. Grochowicz, M.; Pączkowski, P.; & Gawdzik, B. Investigation of the Thermal Properties of Glycidyl Methacrylate–Ethylene Glycol Dimethacrylate Copolymeric Microspheres Modified by Diels–Alder Reaction. *J. Therm. Anal. Calorim*. **2018**, *133*, 499–508.
15. *Gummy Bear* by cerberus333. Thingiverse, 2014. https://www.thingiverse.com/thing:233643 (accessed 2024-07-18).
16. *Costume Cow Horns* by katcoyote. Thingiverse, 2020. https://www.thingiverse.com/thing:4652655 (accessed 2024-07-18).

17. *Horseshoe (подкова С НОВЫМ ГОДОМ)* by shura2000. Thingiverse, 2018. https://www.thingiverse.com/thing:3164661 (accessed 2024-07-18).
18. *Simple Ring* by zagaris123. Thingiverse, 2017. https://www.thingiverse.com/thing:2373931 (accessed 2024-07-18).
